# Synergistic Effect of Small Molecular Organic Matter and Functional Groups in Coal on Methane Adsorption

**DOI:** 10.1021/acsomega.3c07419

**Published:** 2023-12-18

**Authors:** Huan Zhang, Chuang Song, Xiangyang Zhang, Hongbao Zhao, Shuangli Du, Wenfei Tao, Haonan Chai, Yaping Lv

**Affiliations:** † College of Safety and Emergency Management Engineering, Taiyuan University of Technology, Taiyuan 030024, China; ‡ Key Laboratory of Safety and High-efficiency Coal Mining, Ministry of Education Anhui University of Science and Technology, Huainan 232001, China; § School of Energy and Mining Engineering, China University of Mining and Technology (Beijing), Beijing 100083, China; ∥ Huayang New Material Technology Group Co., Ltd., Yangquan 045000, China

## Abstract

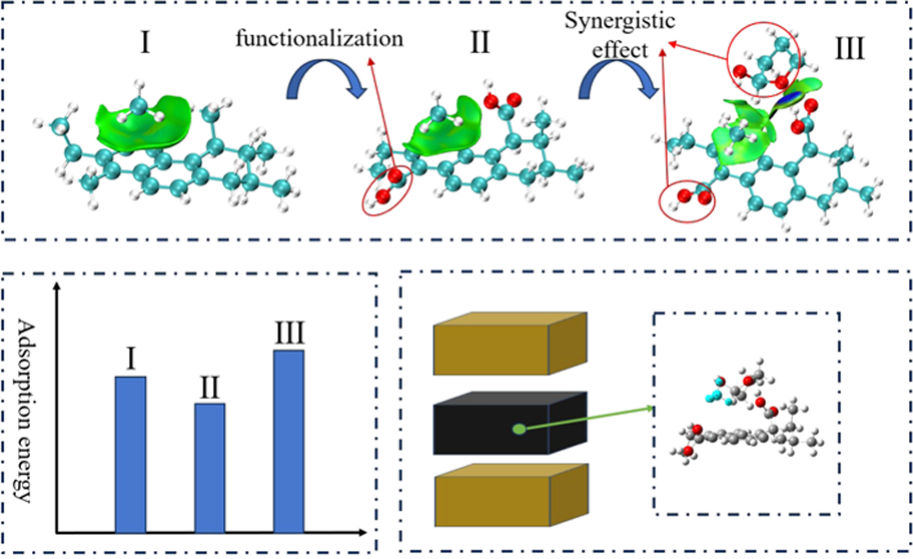

Functional groups and small-molecule organic matter are two key parts of coal. To explore the microscopic mechanism underlying the synergistic effect of both parts on methane adsorption, the oxygen-containing (−OH, −COOH, and –C=O) and nitrogen-containing (−NH_2_) functional groups and two common small molecular organic matter methylbenzene and tetrahydrofuran-2-alcohol in coal are selected. The quantum chemical meta-GGA functional method is used to optimize all structures. The electrostatic potential analyses, weak interaction analyses, and theory of atoms in molecules have been used to delve further into the nature of this synergistic effect. Our results show that functional groups inhibit methane adsorption by coal molecules, and the inhibition effect is enhanced in the presence of methylbenzene. Interestingly, the synergistic effects between functional groups and small molecular organic matter are changed from inhibition to promotion after introducing tetrahydrofuran-2-alcohol, wherein −COOH has the best synergistic effect. This work not only offers a theoretical foundation for exploring the synergistic effect of small molecular organic matter and functional groups on methane adsorption by coal molecules but also lays a foundation for further research on gas prevention and extraction.

## 1 Introduction

China is a developing country dominated by coal resources. The remaining coal proven reserves account for about 13.33% of the world’s total reserves, ranking third in the world.^1^ Coal production and consumption are both 29 × 10^8^ t of standard coal, ranking first in the world.^2^ Methane (CH_4_) is commonly understood as a coal seam gas, a kind of gas associated with coal. It is widely known that CH_4_, a kind of efficient clean energy with great energy value and application prospect under the promotion of sustainable development strategy,^3−5^ becomes one of the most popular research topics in both academia and industry. However, CH_4_ is prone to explosion^6,7^ and outburst accidents^8,9^ in the process of coal seam mining because of its special characteristics, such as rapid diffusion, intense burning, and explosion. Therefore, understanding and mastering the behavior of CH_4_ adsorption in coal seams holds significant guiding importance for the mitigation and management of these disasters.

Functional groups and small molecular organic matter are important components of coal, many efforts^10−15^ have proved that these functional groups have a great influence on CH_4_ sorption in coal seams. Xiang et al.^16^ used density functional theory (DFT) to calculate the adsorption energies of different functional groups on CH_4_, revealing the effect of functional groups on CH_4_ adsorption. Wang et al.^17^ studied the interaction between oxygen-containing functional groups and water molecules on the surface of coal by using DFT. The results show that oxygen-containing functional groups can change the wettability of the coal surface and enhance the interaction between coal and water. Zhang et al.^18^ studied the adsorption behavior of organic sulfur on coal using quantum chemical calculations and concluded that O_2_ is prefered to adsorb on organic sulfur. Yutong et al.^19^ used DFT to study the promoting effect of CO_2_ adsorption on CH_4_ desorption from coal vitrinite. It is pointed out that competitive adsorption will occur when CO_2_ and CH_4_ are simultaneously adsorbed in the vitrinite of coal, thus promoting the desorption of CH_4_. Zhang et al.^20^ found that the oxygen- and nitrogen-containing functional groups, affecting the CH_4_ adsorption of coal through exploring the adsorption mechanisms of CH_4_ on surface functional groups in the coal matrix. Zhou et al.^21^ discussed the influence of functional groups in coal on the adsorption heat of CH_4_ and found that the oxygen-containing functional groups can affect the adsorption heat of CH_4_ by changing the adsorption potential of CH_4_. Jia et al.^22^ modified the functional groups of activated carbon and calculated the adsorption of CH_4_ on the modified activated carbon by DMol^3^ module in Materials Studio. Their results showed that the adsorption of CH_4_ on coal is hindered by oxygen-containing functional groups on activated carbon. Gensterblum et al.^23^ investigated the adsorption capacities of different maturity coals for CH_4_ and CO_2_ with the change of water content and reached the conclusion that the main adsorption site of competitive adsorption between CH_4_ and H_2_O is oxygen-containing functional groups. Nishino^24^ studied the adsorption of water vapor and carbon dioxide by −COOH on the surface of various types of coal at room temperature and found that the adsorption capacities of these molecules were directly proportional to the square root of the carboxyl concentration and had no relation with partial pressure or gas type. It is worth to note that small molecular organic matter is also an important component of coal, which is closely correlated with the physical properties of coal (porosity, adsorption, etc.)^25^ and affects the adsorption properties of coal. Many domestic and international scholars have made significant efforts to investigate the influence of small-molecule organic matter in coal. Yao et al.^26^ found that small molecular organic matter in coal affects the CH_4_ adsorption of coal by changing the surface functional groups and pore structure of coal according to the experimental observation. Theoretical calculation by Mao et al.^27^ demonstrated that there existed interactions between small molecular organic matter and CH_4_ molecule, leading to the charge changes of CH_4_. Thus, the van der Waals interactions between CH_4_ and the surface of coal have been changed, resulting in different adsorption properties of coal to CH_4_. Ji et al.^28^ analyzed the anthracite and bituminous coal after tetrahydrofuran extraction and found that the CH_4_ adsorption characteristics of coal after extraction were determined by the expansion of pore structures and the reduction of small molecular organic matter. Yao et al.^29^ found that the hydrophobic properties of coking coal increased as the hydrophilic functional groups decreased after tetrahydrofuran extraction. Peng et al.^30^ studied the impact of small molecular organic matter on CH_4_ adsorption in different coal ranks. For low-rank coal containing a significant amount of small molecular organic matter, the presence of these matters leads to the transformation of macropores into numerous micropores, enhancing the adsorption of CH_4_. Conversely, in medium-rank coal with an equivalent concentration of small molecular organic matter, these matters tend to close the micropores, resulting in a decrease in the CH_4_ adsorption capacity.

The studies described above analyzed the influence of functional groups and small molecular organic matter on gas adsorption. Do some synergistic effects exist between the functional groups and small molecular organic matter? And how does it influences the adsorption of CH_4_? To validate the above hypothesis, we selected a simplified coal molecular fragment for functionalization transformation and two common small molecular organic matter species (tetrahydrofuran-2-alcohol and methylbenzene) in coal for research in this study. The DFT method was employed to study the adsorption characteristic of CH_4_ in coal under the synergistic influence of functional groups and small molecular organic matter. In addition, the nature of the interaction was further investigated by a variety of analytical methods, such as adsorption energies and structural characteristics analyses, the theory of atoms in molecules, and the independent gradient model analyses based on the Hirschfeld partition. By studying the influences of synergistic effects of small molecular organic matter and functional groups in coal on CH_4_ adsorption, the adsorption mechanism of coal can be deeply understood. It not only provides a scientific basis for coalbed methane mining and coal utilization but also contributes to reducing greenhouse gas emissions, achieving clean energy transformation and sustainable development.

## 2 Calculation Models and Methods

### 2.1 Models

**Figure 1:**
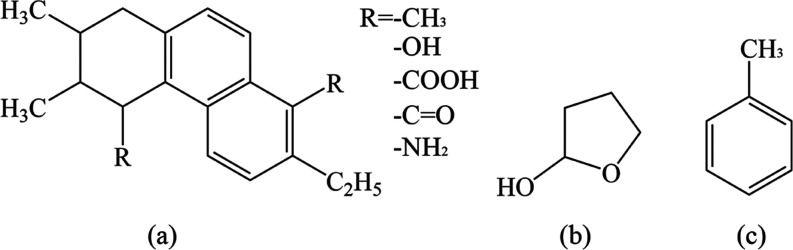
Structures of coal molecular fragments and small molecular organic matter: (a) coal molecule, (b) tetrahydrofuran-2-alcohol, and (c) methylbenzene.

A simplified model of coal molecule (shown in Figure 1a) has been invested in this work due to the complexity of the coal macromolecular structure, wherein *R* represents different functional groups. The model is obtained by functionalization modification on the basis of high-volatile bituminous coal.^31^Figure 1b,c shows the small molecular organic matter tetrahydrofuran-2-alcohol and methylbenzene in coal. Tetrahydrofuran-2-alcohol and methylbenzene are represented by Tet and Met in the following text. The coal molecules containing −CH_3_, −OH, −COOH, –C=O, and –NH_2_ are represented by coal–CH_3_, coal–OH, coal–COOH, coal–C=O, and coal–NH_2_, respectively.

### 2.2 Theoretical Methods

GaussView is used to construct the molecular structure model, and the theoretical calculations are carried out by Gaussian 16 software. The Meta-GGA functional M062X method,^32^ widely used in exploring noncovalent interactions, has been employed to optimize the structures of coal molecules, small molecular organic matter, CH_4_, and all possible adsorption configurations. Frequency calculations were performed at the M062*X*/6-311++G (d, p) level to confirm that all optimized structures represent local minima on their respective potential energy surfaces. The calculation method takes into account the effect of dispersion on the adsorption energy. The distribution of molecular electrostatic potential holds great significance in predicting reaction sites and analyzing weak interactions between molecules.^33^ In addition, the theory of atoms in molecules^34^ and the independent gradient model analyses based on the Hirschfeld partition (IGMH)^35^ are used to analyze the nature of these interactions among various coal molecules, small molecular organic matter, and CH_4_. The theory of atoms in molecule analyses proposed by Bader^36^ is widely used to analyze the covalent and noncovalent bond interactions. By studying the characteristics of the electron density at the bond critical point (BCP), we can understand some important information about intermolecular interactions. In addition to visually representing the weak interaction region, the IGMH technique utilizes various colors to indicate the type and strength of interaction by projecting the sign(λ_2_)ρ function onto the isosurface diagram. In order to achieve the above content, the following software was used for analysis. Multiwfn^37^ is a powerful program for realizing electronic wave function analysis. VMD^38^ is a software application that can visualize and analyze data in 3D mode. Gnuplot can gradually set up or modify the drawing environment and graphically describe data or functions so that we can do further analysis using graphics. The optimized wave function files of the surface structure of coal molecules, CH_4_, and small molecular organic matter are imported into the Multiwfn program as input files for postprocessing. The calculation results are presented by using the visualization program VMD and IGMH isosurface diagrams drawn by Gnuplot software. Finally, the electrostatic potential diagrams marked with extreme values, topological path diagrams, IGMH isosurface diagrams, and scatter plots are obtained.

Adsorption energy (Δ*E*_interaction_, kJ/mol) can be used to determine the strength of interaction among coal molecules, small molecular organic matter, and CH_4_. BFEE2^39^ is widely used to calculate the binding energy. In this paper, the adsorption energy is calculated by Formula 1
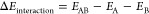
1where *E*_AB_ (kJ/mol) is the energy of stable structure after adsorption between A and B molecules; *E*_A_ and *E*_B_ (kJ/mol) represent the energies of A and B molecules, respectively.

Formula 2 can be used to approximately calculate the adsorption energy Δ*E*′ (kJ/mol) for a coadsorption system of two substances on the surface of coal molecules
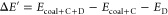
2where *E*_coal+C+D_ (kJ/mol) is the energy of complex formed by coal molecules with C and D; *E*_coal+C_ (kJ/mol) is the energy when the adsorption of coal molecules and C reaches equilibrium; and *E*_D_ (kJ/mol) is the energy of the D molecule.

**Figure 2:**
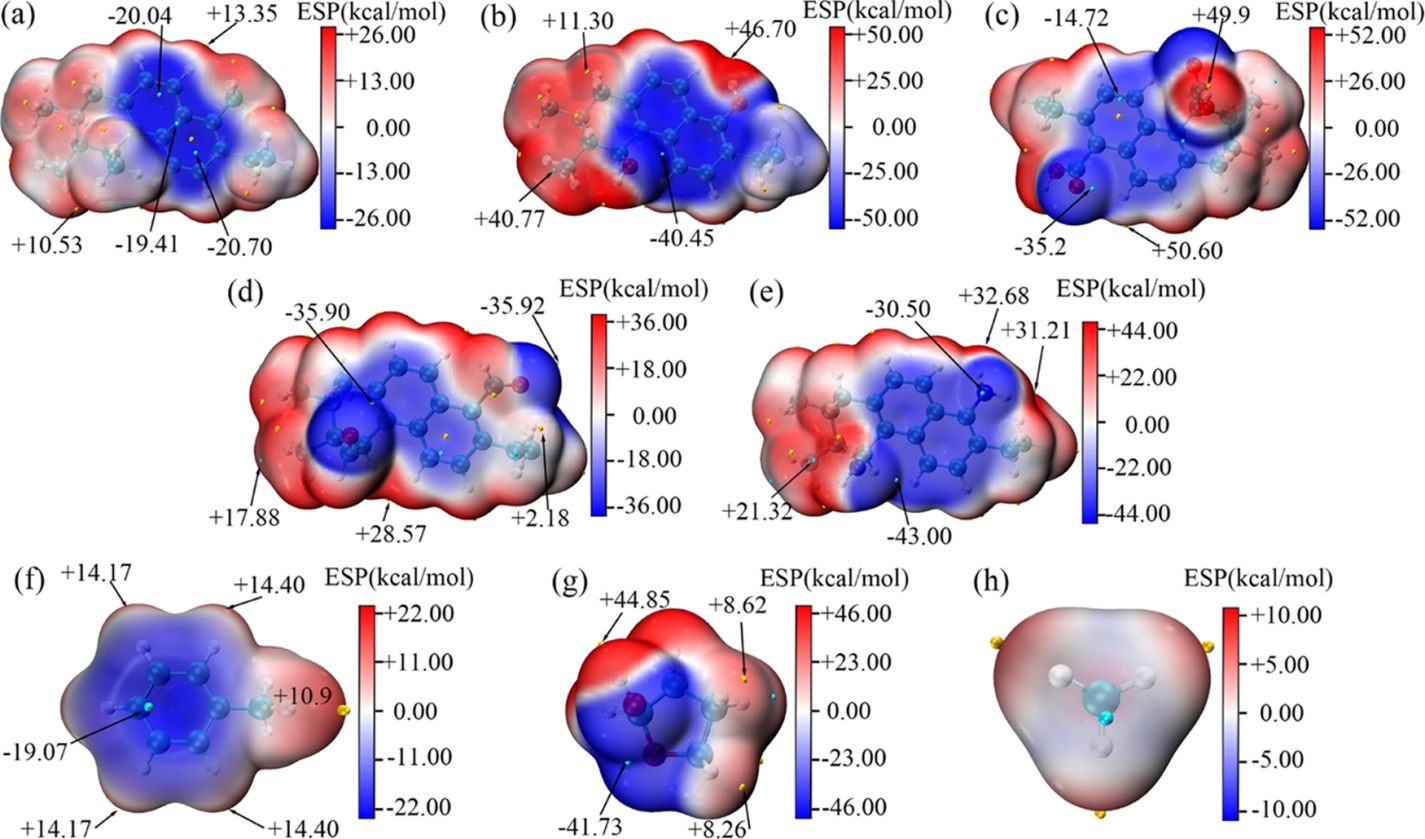
Electrostatic potential diagrams of coal–CH_3_ (a), coal–OH (b), coal–COOH (c), coal–C=O (d), and coal–NH_2_ (e) and the small molecular organic matter of Met (f), Tet (g), and CH_4_ (h), respectively.

## 3 Results and Discussion

### 3.1 Electrostatic Potential Analyses

Electrostatic potential is of great importance in the investigation of the prediction of reaction sites. By utilizing the diagrams of electrostatic potential, a visual understanding of the interactions between molecules, elucidating the strength and mode of binding can be obtained. In order to predict the adsorption sites of CH_4_ over coal molecules, Tet and Met, the electrostatic potential diagrams have been plotted as shown in Figure 2. It is clear that the electrostatic potential distribution range is −20.07 to +13.35 kcal/mol when there is no functional group (coal–CH_3_) on the coal surface. However, large differences are presented between positive and negative electrostatic regions in the coal surface with functional groups, which indicates that the presence of functional groups on the coal surface enlarges the disparity in electrostatic potential. In addition, it is obvious that the π electron clouds on the surface of an aromatic ring make the electrostatic potential negative, and the H atom is positive due to the electronegativity of C and N. In the nonaromatic region, the maximum surface electrostatic potentials of coal molecules are in order of −COOH > −OH > –NH_2_ > –C=O, which may be caused by the different polarity of each functional group. Among them, the H atom of coal–COOH exhibited the most prominently positive electrostatic potential (+50.60 kcal/mol), while the N atom on the coal–NH_2_ molecular surface displayed the most negative electrostatic potential (−43.00 kcal/mol). Taking adsorption between coal–NH_2_ and Tet as an example, on the surface of Tet, the largest positive electrostatic potential was measured at +44.85 kcal/mol. Therefore, the optimal adsorption site is between the N of the coal–NH_2_ surface and the −OH of the Tet surface according to the electrostatic complementation. Other adsorption configurations can be obtained by the same method. Based on the observations from Figure 2, we speculate that the favorable adsorption sites of CH_4_ on coal molecules are the positions close to the functional groups on the benzene ring.

### 3.2 Adsorption of Single Small Molecule with the Coal Molecule

**Figure 3:**
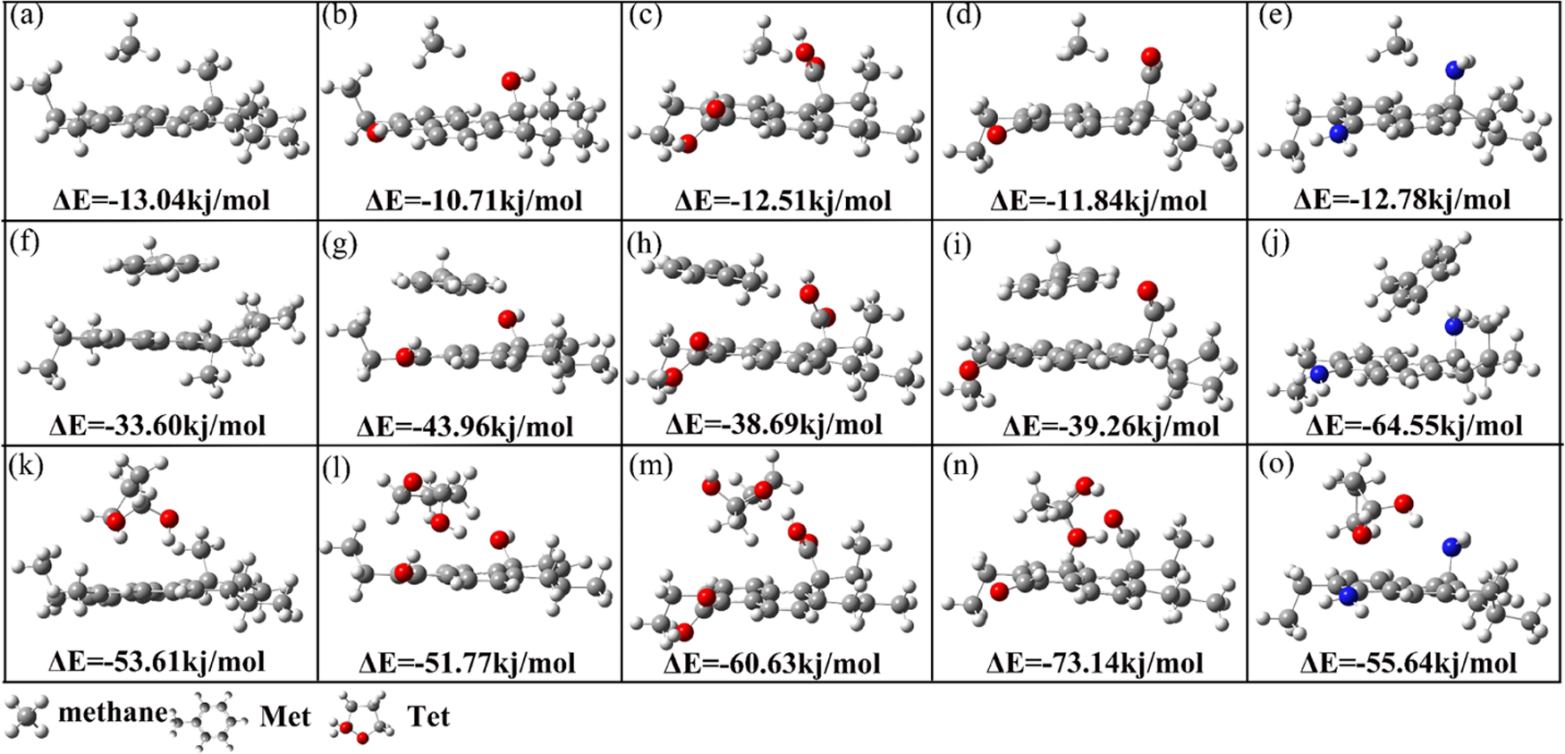
Stable adsorption configurations of different complexes. (a–e), (f–j), (k–o) are the CH_4_, Met, and Tet adsorbed on coal–CH_3_, coal–OH, coal–COOH, coal–C=O, and coal–NH_2_, respectively. Δ*E* is the adsorption energy of small molecules on the coal molecule.

Figure 3 shows the equilibrium configurations of CH_4_, Met, and Tet adsorbed on various coal molecules. It can be seen that CH_4_ and the two kinds of small molecular organic matter can be well adsorbed on coal molecules. CH_4_ is adsorbed above the benzene ring near the functional groups, which is in agreement with the prediction of electrostatic potential analyses. Met and Tet are adsorbed above the benzene ring. Met mainly exists in π–π interaction mode, and Tet forms a hydrogen bond with functional groups.

#### 3.2.1 CH_4_ Adsorption on the Coal Molecule

Table 1 provides the structural parameters for the adsorption equilibrium of CH_4_ on coal molecule surfaces. The adsorption energies of CH_4_ on coal–CH_3_, coal–OH, coal–COOH, coal–C=O, and coal–NH_2_ are −13.04, – 10.71, – 12.51, – 11.84, and −12.78 kJ/mol, respectively. The calculated adsorption energies are consistent with the range of physical adsorption energies demonstrated by previous experimental and theoretical works.^40−43^ From the comparison of adsorption energies, it can be seen that the order of adsorption capacities of CH_4_ on coal molecules is –NH_2_> −COOH> –C=O> −OH, which aligns well with the order of the CH_4_ C–H bond length changes (−NH_2_> −COOH> –C=O> −OH) after being adsorbed by various coal molecules. In simpler terms, as the adsorption energies between coal molecules and CH_4_ increase, there is a corresponding increase in the alteration of the CH_4_ C–H bond. Moreover, the adsorption energies of coal molecules with functional groups are all weaker than those of coal–CH_3_ (−13.04 kJ/mol), indicating that the functional groups hinder the adsorption of CH_4_ by coal molecules.

**Table 1:** Equilibrium Parameters of CH_4_ Adsorption on Different Coal Molecules

adsorption configuration	C–H bond (Å) before adsorption	C–H bond (Å) after adsorption	adsorption energy (kJ/mol)
coal–CH_3_·CH_4_	1.08927	1.09058	–13.04
coal–OH·CH_4_	1.08927	1.09033	–10.71
coal–COOH·CH_4_	1.08927	1.09078	–12.51
coal–C=O·CH_4_	1.08927	1.09061	–11.84
coal–NH_2_·CH_4_	1.08927	1.09080	–12.78

**Figure 4:**
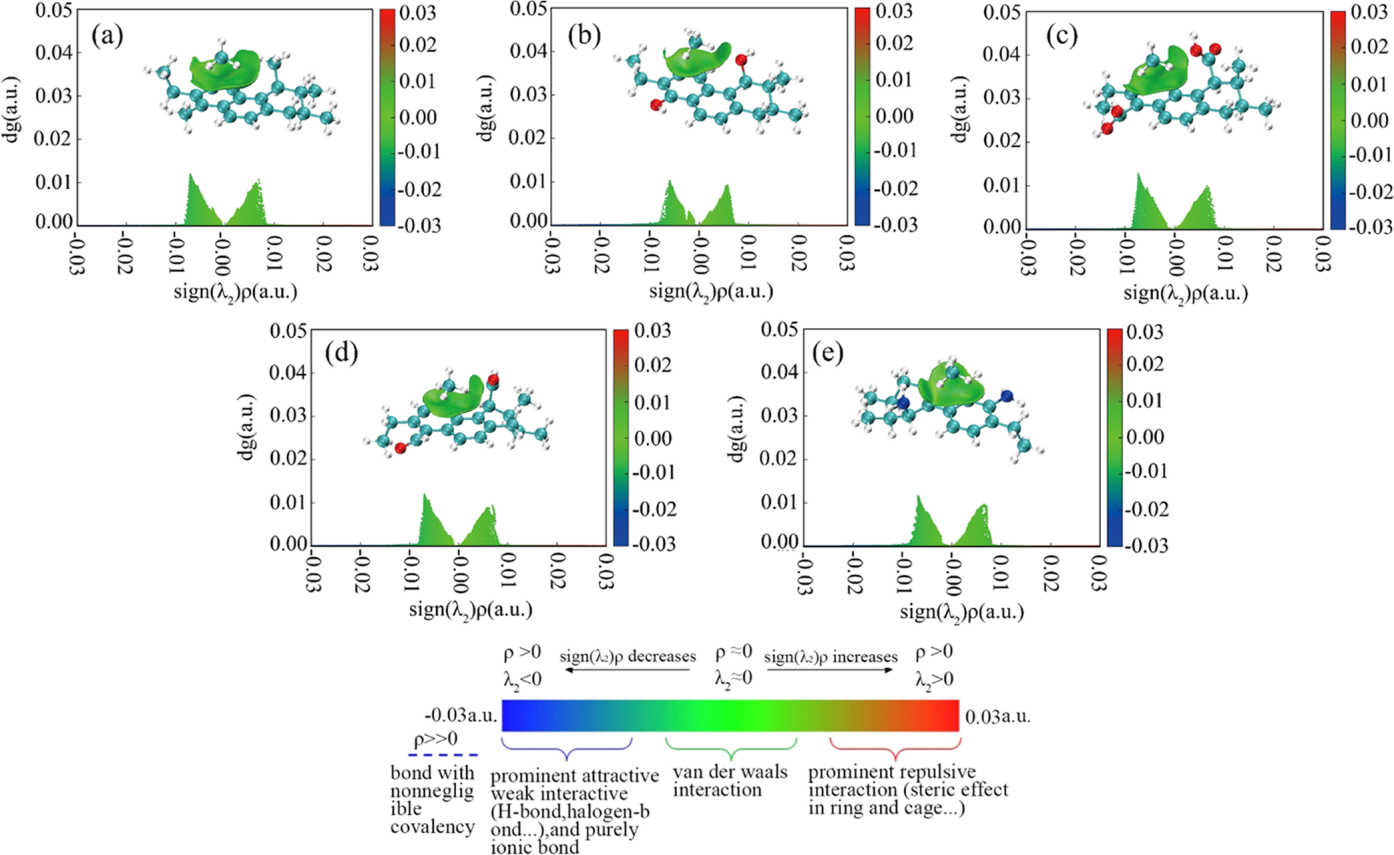
Isosurface diagrams and scatter plots of CH_4_ adsorbed by different coal molecules: (a–e) coal–CH_3_, coal–OH, coal–COOH, coal–C=O, and coal–NH_2_, respectively.

Isosurface diagrams and scatter plots of CH_4_ adsorption by different coal molecules are shown in Figure 4. In IGMH analyses, the values of different sign (λ_2_)ρ are presented by different colors on the isosurface. And these colors can be used to distinguish the types and intensities of the interaction. The intensity of the interaction increases as the color of the isosurface deepens. It is apparent that there is a green region between CH_4_ and the benzene ring and the functional groups in coal molecules, demonstrating that CH_4_ interacts with the benzene ring and functional groups. The corresponding values sign(λ_2_)ρ in the scatter plots are between −0.02 and 0.01 au, indicating that the interactions are dominated by van der Waals interactions.

**Figure 5:**
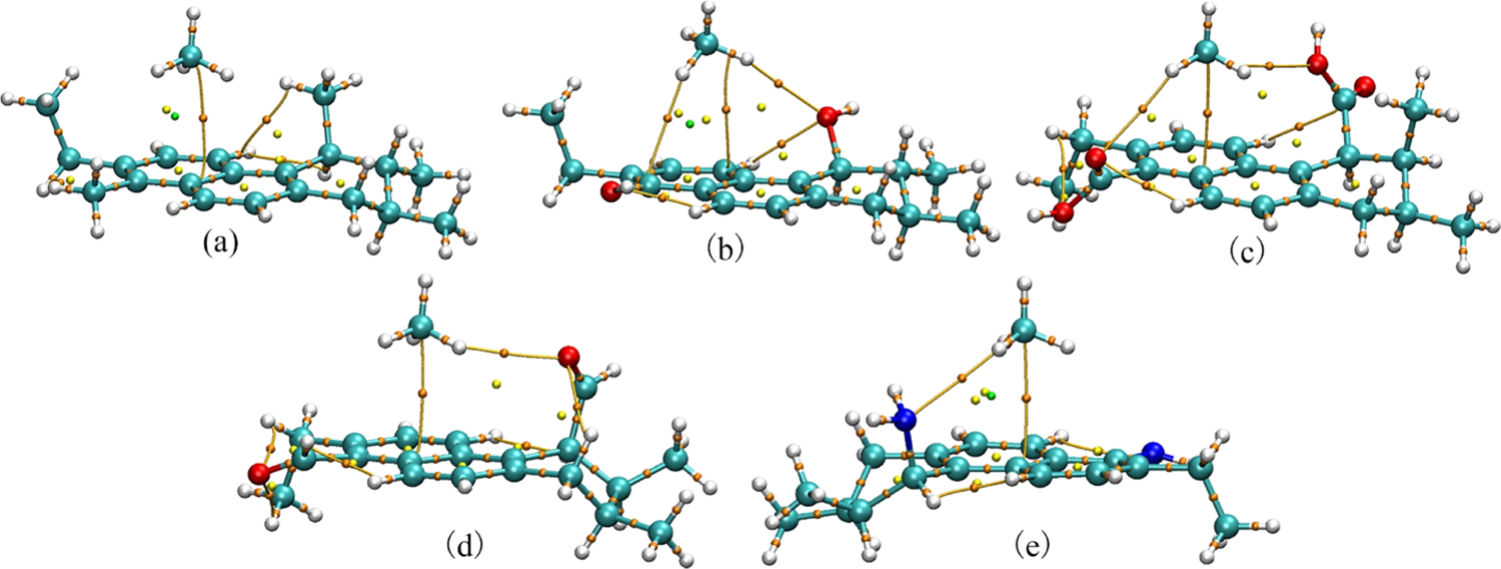
AIM topological paths of CH4 adsorption on coal–CH_3_ (a), coal–OH (b), coal–COOH (c), coal–C=O (d), and coal–NH_2_ (e).

Figure 5 and Table 2 are the AIM topology path diagrams and topology parameter table, respectively. AIM is based on the critical point and electron density distribution in the electron density gradient field and identifies the interaction between two bonds by analyzing the electron density ρ_r_ and Laplacian value ∇^2^ρ_r_ at the BCP. The electron density (ρ_r_) and its Laplacian value (∇^2^ρ_r_), the kinetic energy density (*G*_r_), the potential energy density (*V*_r_), the total electron energy density (*H*_r_), and the ratio (|*V*_r_|/*G*_r_) at BCP are shown in Table 2. The interaction paths between atoms with mutual attractions can be obtained through the AIM topological analyses of the adsorption configuration.^44,45^ The types and relative strengths of the interactions between atoms can be more accurately judged according to the topological parameters of the BCP.

**Table 2:** Topological Parameters at BCP in Adsorption Configurations

configuration	BCP	ρ_r_	∇^2^ρ_r_	*G*_r_	*V*_r_	*H*_r_	|*V*_r_|/*G*_r_
coal–CH_3_·CH_4_	(1)C(coal)·C(CH_4_)	0.0070	0.0249	0.0050	–0.0037	0.0012	0.7400
coal–OH·CH_4_	(2)C(coal)·H (CH_4_ right)	0.0059	0.0164	0.0036	–0.0030	0.0005	0.8333
	(3)O(coal)·H (CH_4_ left)	0.0060	0.0197	0.0043	–0.0036	0.0007	0.8372
coal–C=O·CH_4_	(4)O(coal)·H(CH_4_)	0.0061	0.0200	0.0043	–0.0037	0.0007	0.8605
coal–COOH·CH_4_	(5)O(coal left) H(CH_4_)	0.0045	0.0143	0.0031	–0.0027	0.0005	0.8710
	(6)O(coal right) H(CH_4_)	0.0056	0.0212	0.0044	–0.0035	0.0009	0.7955
coal–NH_2_·CH_4_	(7)N(coal)·H(CH_4_)	0.0067	0.0183	0.0040	–0.0034	0.0006	0.8500

It can be concluded from Table 2 that the values of ∇^2^ρ_r_ are all in the range of 0.0143–0.0269 au in each adsorption configuration. Meanwhile, the potential energy density *V*_r_ is less than 0 and the total energy density *H*_r_ is greater than 0, indicating that weak hydrogen bonds are formed in these adsorption configurations, and these interactions between molecules are mainly noncovalent bonds. The order of ρ_r_ values of these adsorption configurations is (1) > (4) > (6) > (5) > (3) > (2). Of the four CH_4_ adsorption configurations on coal molecules containing functional groups, the coal–NH_2_ and CH_4_ interaction exhibits the highest level of strength.

Based on the above analyses, it can be inferred that the primary governing forces in the interactions between CH_4_ and coal molecules are attributed to van der Waals interactions. In addition, these interactions are weakened after functional groups are introduced into coal molecules.

#### 3.2.2 Adsorption of Small Molecular Organic Matter on the Coal Molecular Surface

The content presented above explores the impact of various functional groups on the adsorption of CH_4_ by coal molecules. In order to find out how small molecular organic matter affects the adsorption of CH_4_ by coal, the adsorption properties of small molecular organic matter over the coal molecular surface are investigated in this part. Figure 3f–o shows the adsorption equilibrium configurations of small molecular organic matter over the surface of various coal molecules. Clearly, the most stable adsorption site for Met is above the benzene ring of the coal molecule, whereas for Tet, the functional group side chains are the most stable adsorption site. In addition, the adsorption energies of Met on coal–OH, coal–COOH, coal–C=O, and coal–NH_2_ are −43.96, – 38.69, – 39.26, and −64.55 kJ/mol, respectively. Met has the largest adsorption capacity on coal–NH_2_, and the smallest adsorption capacity appears between coal–COOH and Met. Moreover, one can find that the adsorption energy of Met over coal–CH_3_ is −33.60 kJ/mol. Therefore, it is reasonable to deduce that the adsorption of Met by coal molecules is enhanced due to the presence of functional groups. The adsorption energies of Tet adsorbed on coal–OH, coal–COOH, coal–C=O, and coal–NH_2_ are −51.77, – 60.63, – 73.14, and −55.64 kJ/mol, respectively. The coal–C=O has the largest adsorption capacity for Tet, and coal–OH shows the weakest adsorption capacity for Tet. The adsorption energy of coal–CH_3_ for adsorbing Tet is −53.61 kJ/mol. Therefore, the adsorption of Tet on coal molecules is inhibited by −OH while promoted by introducing −COOH, –C=O, and –NH_2_. Through the above analysis, it can be deduced that the adsorption strength of the two types of small molecular organic matter on coal molecules is considerably greater than that of CH_4_. The adsorption energies sequence is Tet > Met > CH_4_.

**Figure 6:**
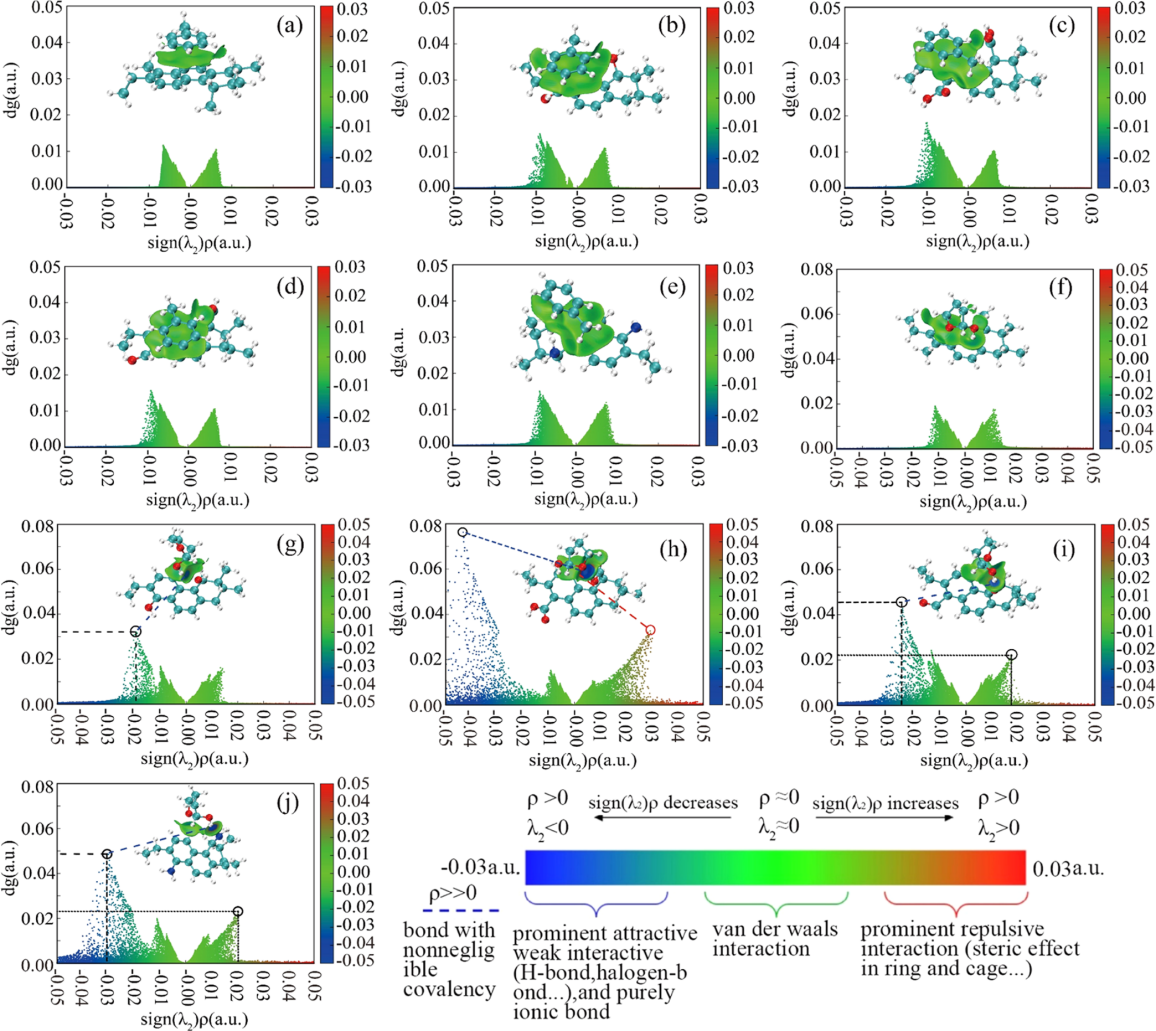
Isosurface diagrams and scatter plots of adsorption configurations formed by various coal molecules and small molecular organic matter, Met and Tet ((a–e) and (f–j) coal–CH_3_, coal–OH, coal–COOH, coal–C=O, and coal–NH_2_, respectively).

IGMH analyses are used to further analyze the nature of the interactions between coal molecules and small molecular organic matter. The corresponding subsurface diagrams and scatter plots of the adsorption complexes are shown in Figure 6. It is apparent that the adsorptions between Met and coal molecules are mainly dominated by π–π interaction, which is reflected in a light green color in the isosurface diagrams. For the interactions between Tet and coal molecules, not only van der Waals interaction but also hydrogen bonds are observed in the isosurface diagrams and scatter plots. This is consistent with the results predicted by electrostatic potential analyses. Figure 6f,g shows the isosurface diagrams and scatter plots of Tet adsorbed by coal–CH_3_ and coal–OH. In Figure 6f, only a green area is presented, indicating that there is only a van der Waals effect between Tet and coal–CH_3_. Interestingly, in Figure 6g, a light blue region is observed, indicating the interaction between the hydrogen in the Tet hydroxyl group and the oxygen in the hydroxyl group of coal–OH. This corresponds to the presence of a weak hydrogen bond in the scatter plot. After a comprehensive comparison, it is reasonable to deduce that van der Waals interactions are the dominant forces in Figure 6f,g, and the interaction area in Figure 6f is larger than that in Figure 6g. Figure 6h–j shows the isosurface diagrams and scatter plots of Tet adsorbed by coal–COOH, coal–C=O, and coal–NH_2_, respectively. Clearly, two different types of hydrogen bonds are formed between Tet and the three coal molecules. The hydrogen bond between coal–COOH and Tet is formed by the hydrogen in the O–H bond in –COOH and oxygen in Tet. However, the coal–C=O is established through the hydrogen in the O–H bond of COOH and the oxygen in the functional groups. Meanwhile, coal–NH_2_ involves hydrogen in the O–H bond of Tet and nitrogen in the functional groups. The different hydrogen bond formation modes lead to a different hydrogen bond strength. It can be seen from the scatter plots that the blue peaks of coal–C=O and coal–NH_2_ are in the range of −0.03 to −0.02 au, while the blue peak exists between −0.04 and −0.02 au for −COOH. In addition, the blue peak corresponding to coal–COOH is the highest. This indicates that the hydrogen bond formed between coal–COOH and Tet is the strongest. However, some red scatter plots are found in the 0.02–0.03 au interval on the right side of the scatter plot in Figure 6h, which is due to the repulsion of oxygen in Tet and oxygen in coal molecules. This repulsion weakens the adsorption capacity of coal–COOH to Tet. In conclusion, −COOH, –C=O, and –NH_2_ functional groups promote the adsorption of small molecular organic matter on coal. However, the presence of −OH exhibits distinct effects. It promotes the adsorption of Met by coal while inhibiting the adsorption of Tet by coal.

After the analyses of the adsorption behaviors of small molecular organic matter on the coal molecule, it can be found that the adsorption energies of Met and Tet on the coal molecule are greater than that of CH_4_. In addition, the adsorption sites of Met on coal molecules are the same as those of CH_4_. Therefore, we speculate that the adsorption of small molecular organic matter over coal molecules has a certain influence on the adsorption of CH_4_ by coal molecules.

#### 3.2.3 Adsorption of CH_4_ on Coal–Small Molecular Organic Matter

In order to explore the synergistic effect of small molecular organic matter and functional groups within coal molecules on CH_4_ adsorption, the adsorption of CH_4_ on complexes formed by coal and small molecular organic matter has been investigated. The adsorption energies of CH_4_ adsorbed by Met and Tet are calculated to be −7.30 and −19.62 kJ/mol. In comparison to the adsorption energies of CH_4_ onto coal molecules, the order of the adsorption energy for CH_4_ is Tet > coal molecule > Met. It is evident that Tet exhibits stronger adsorption capacities for both coal and CH_4_ compared to Met, indicating that small molecular organic matter in coal can affect the adsorption of CH_4_.

**Figure 7:**
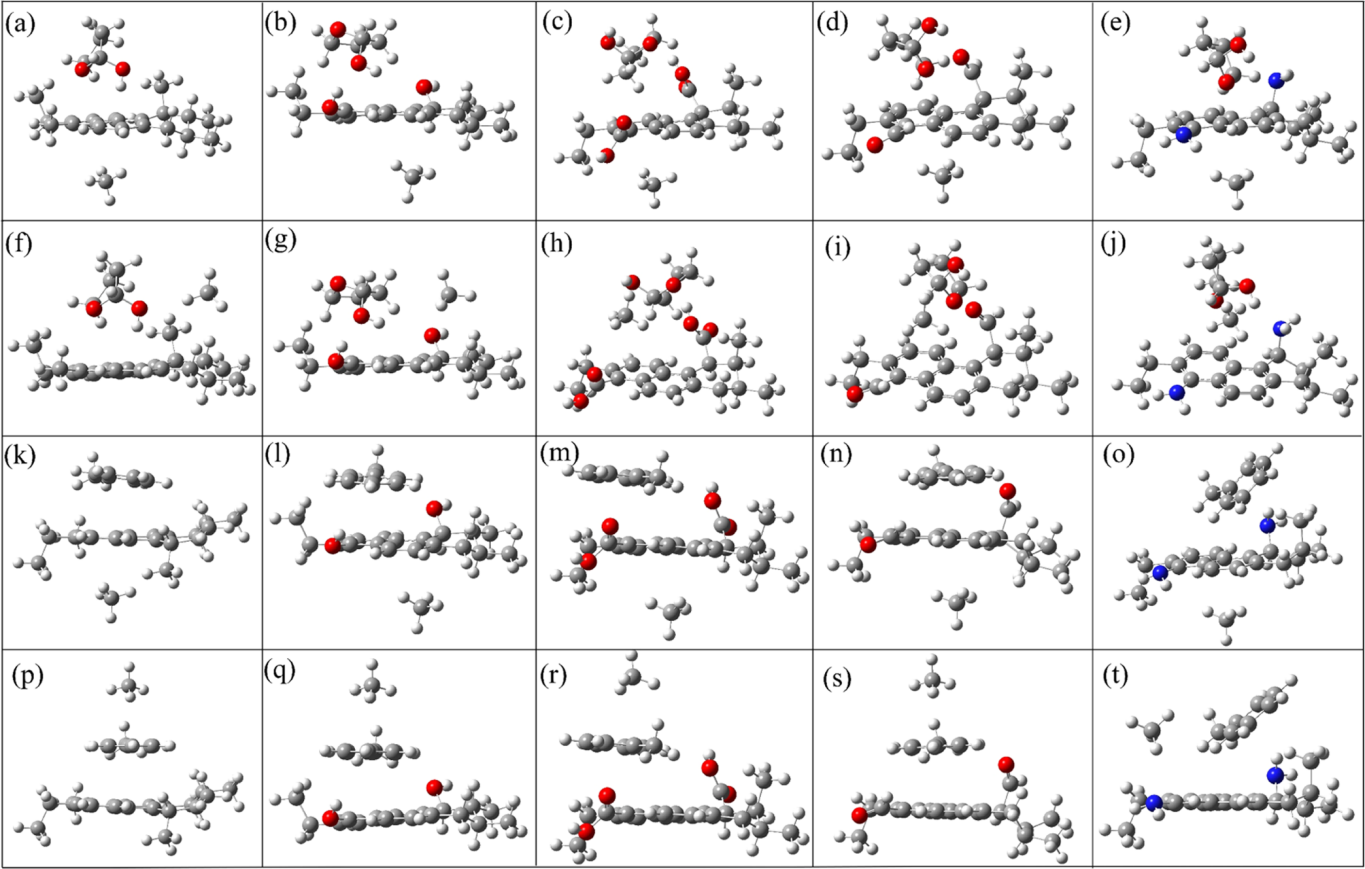
Different adsorption configurations of CH_4_ on coal–Tet and coal–Met mixed systems. (a–e) and (f–j) CH_4_ adsorbed on coal–CH_3_–Tet, coal–OH–Tet, coal–COOH–Tet, coal–C=O–Tet, and coal–NH_2_–Tet mixed systems, respectively. (k–o) and (p–t) CH_4_ adsorbed on coal–CH_3_–Met, coal–OH–Met, coal–COOH–Met, coal–C=O–Met, and coal–NH_2_–Met mixed systems, respectively. (a–e) and (k–o) The cases that small molecular organic matter and CH_4_ are adsorbed on the opposite sides of coal molecule. (f–j) and (p–t) Small molecular organic matter and CH_4_ are on the same side of the coal molecule.

**Figure 8:**
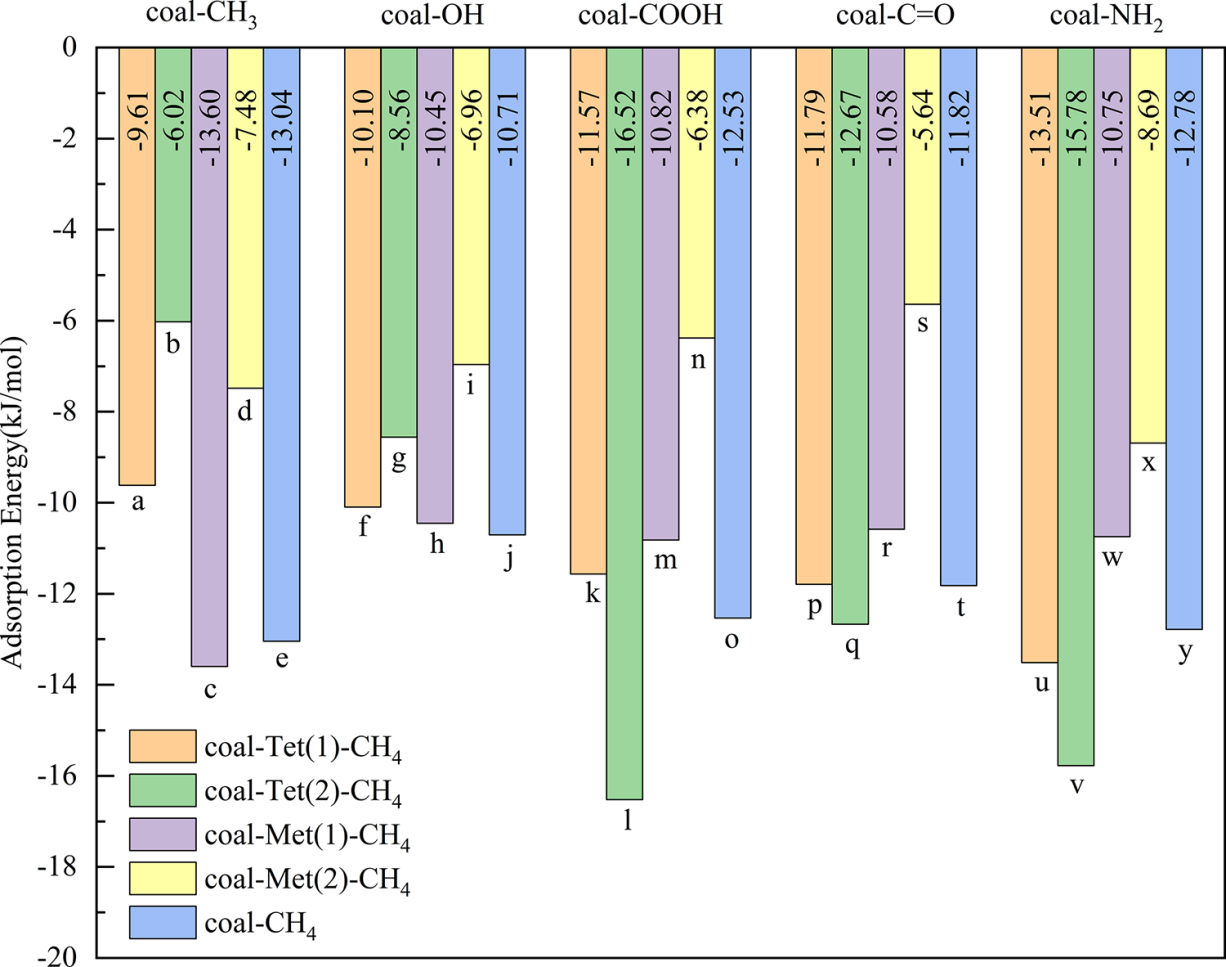
Adsorption energies of CH_4_ on coal–Tet, coal–Met, and coal single molecules. (1) represents CH_4_ and small molecular organic matter on the two sides of coal and (2) represents them on the same side of coal.

Using the electrostatic potential analysis method, the electrostatic potential diagrams of coal–small molecule complexes are analyzed. The optimal adsorption configurations and the corresponding adsorption energies of CH_4_ adsorbed on the complexes coal–small molecular organic matter are shown in Figures 7 and 8. Analyzing the adsorption configurations in Figure 7 and comparing the orange and green parts in Figure 8, it is clear that upon the occurrence of the adsorption reaction, Tet occupies the most favorable adsorption site of coal–CH_3_. Hence, CH_4_ is adsorbed to another relatively weak site of coal molecules, resulting in a weak adsorption energy. The phenomenon indicates that the synergistic effect between −CH_3_ and Tet inhibits the adsorption of CH_4_ by coal molecules. In addition, coal–OH, coal–COOH, coal–C=O, and coal–NH_2_ can form hydrogen bonds after adsorbing Tet due to the presence of functional groups. However, the adsorption sites of Tet are different in the optimal adsorption configuration, resulting in different optimal adsorption positions of CH_4_. For coal–OH, the adsorption site of Tet is above the benzene ring, occupying the optimal site for coal molecules adsorbing CH_4_. This leads to the adsorption of CH_4_ on the other side of the coal molecule, and the adsorption strength is weakened. For the optimal adsorption configurations of Tet adsorbed by coal–COOH, coal–C=O, and coal–NH_2_, Tet is adsorbed on the same side of coal molecules. This structure increases the adsorption between the coal molecules and CH_4_. As can be seen from Figure 7, CH_4_ is not only adsorbed by coal molecules but also by Tet, which increases the adsorption energy of coal–COOH, coal–C=O, and coal–NH_2_ to −16.52, – 12.67, and −15.78 kJ/mol, respectively. Compared with coal–CH_4_ (Figure 8 blue parts), it can be seen that the adsorption energies of coal–COOH, coal–C=O, and coal–NH_2_ on CH_4_ becomes stronger, while coal–OH weakens the adsorption energy of CH_4_. Hence, our conclusion is that the synergistic effect of Tet and −OH hampers the adsorption of CH_4_ by coal molecules, whereas it enhances CH_4_ adsorption when working together with −COOH, –C=O, and –NH_2_.

For Met, the compound formed by coal–CH_3_ and Met produces a more favorable action site for CH_4_, enhancing the adsorption of CH_4_. Compared with Figure 8c,d, it can be found that the adsorption energy of CH_4_ on coal–Met(1) (−13.60 kJ/mol) is stronger than that on coal–Met(2) (−7.48 kJ/mol), indicating that CH_4_ prefers to adsorb on the two sides of Met. For coal–OH, coal–COOH, coal–C=O, and coal–NH_2_, the adsorptions of Met and CH_4_ on the same side of coal molecules can lead to a competitive adsorption. Due to the stronger adsorption of Met by coal molecules to CH_4_, Met occupies the optimal adsorption site of the molecule, leading to a reduced adsorption capacity for CH_4_. Interestingly, as can be seen from the purple and yellow parts in Figure 8, there exists a strong adsorption capacity of CH_4_ when Met and CH_4_ occupy the adsorption site on two sides of coal. Upon comparison with coal–CH_4_ (Figure 8e, – 13.04 kJ/mol), it can be found that the adsorption energy of coal–CH_3_–Met (Figure 8c, – 13.60 kJ/mol) is enhanced after introducing Met. The addition of Met weakens the adsorption energies of CH_4_ by coal–OH, coal–COOH, coal–C=O, and coal–NH_2_ to −10.45, −10.82, −10.58, and −10.74 kJ/mol. Therefore, Met can promote the adsorption of CH_4_ by coal–CH_3_, while it can be inhibited by coal–OH, coal–COOH, coal–C=O, and coal–NH_2_.

In addition, compared with the case that CH_4_ adsorbed by coal without functional groups and small molecular organic matter (Figure 8e), the synergistic effect between functional groups −COOH, –NH_2_, and Tet shows promoting effects, while the synergistic effects between −OH and Tet and –C=O and Tet are inhibitory effects. And the synergistic effects among Met and all functional groups also show inhibitory effects.

## 4 Conclusions

The synergistic effects of different functional groups and two small molecular organic matter groups on CH_4_ adsorption by coal molecules have been investigated in the present work. Using different analysis methods, the following conclusions are obtained.

(1) The presence of functional groups in coal molecules hinders the adsorption of CH_4_ onto coal molecules, with the inhibitory effects following this order: −OH> –C=O> −COOH> –NH_2_. The existence of functional groups promotes the adsorption of coal molecules to small molecular organic matter.
(2) Because of competitive adsorption, small molecular organic matter can preferentially occupy the adsorption sites of coal molecules, forming a coal–small molecular organic mixture. The mixture of Tet and coal–COOH, coal–C=O, coal–NH_2_ is beneficial to the adsorption of CH_4_.
(3) The synergistic effects of small molecular organic matter and functional groups on the adsorption of CH_4_ by coal molecules are as follows. Tet, coal–COOH, and coal–NH_2_ can promote the adsorption of CH_4_, while Tet, coal–OH, and coal–C=O show inhibitory effects. In addition, the synergistic effects of Met and all of the functional groups have inhibitory effects on the adsorption of CH_4_.

## Supporting Information Available

The electrostatic potential diagrams of different coal–small molecule organic matter complexes, the adsorption energies of different adsorption sites, the optimized geometries and atom coordinates of all compounds (PDF)
