# Supplementary material for: Synergistic Effect of Small Molecular Organic Matter and Functional Groups in Coal on Methane Adsorption
Source: ACS Omega. 2023 Dec 18;9(1):1156–65. doi: 10.1021/acsomega.3c07419 (PMC10785673; doi:10.1021/acsomega.3c07419)
Supplement: Supplementary file 1 — ao3c07419_si_001.pdf [file ao3c07419_si_001.pdf]

Supporting Information

Synergistic Effect of Small Molecular Organic Matter and Functional Groups in Coal on Methane Adsorption

Huan Zhang,[1,2] Chuang Song,[1] Xiangyang Zhang,[2] Hongbao Zhao,[3] Shuangli Du,[1,4] Wenfei Tao,[1] Haonan Chai,[1] Yaping Lv[1]*

[1]*College of Safety and Emergency Management Engineering, Taiyuan University of Technology, Taiyuan, 030024, China.*

[2]*Key Laboratory of Safety and High-efficiency Coal Mining, Ministry of Education Anhui University of Science and Technology, Huainan, 232001, China.*

[3]*School of Energy and Mining Engineering, China University of Mining and Technology (Beijing), Beijing 100083, China.*

[4]*Huayang New Material Technology Group Co., Ltd., Yangquan, 045000, China.*

Content

Figure S1. The overall electrostatic potential diagrams of coal molecules containing different functional groups and small molecular organic matter

Table S1. Adsorption energy of methane adsorbed at different positions of coal molecules

Table S2. Adsorption energy of small molecular organic matters at different adsorption sites of coal molecules

Optimized geometries and atom coordinates of all compounds

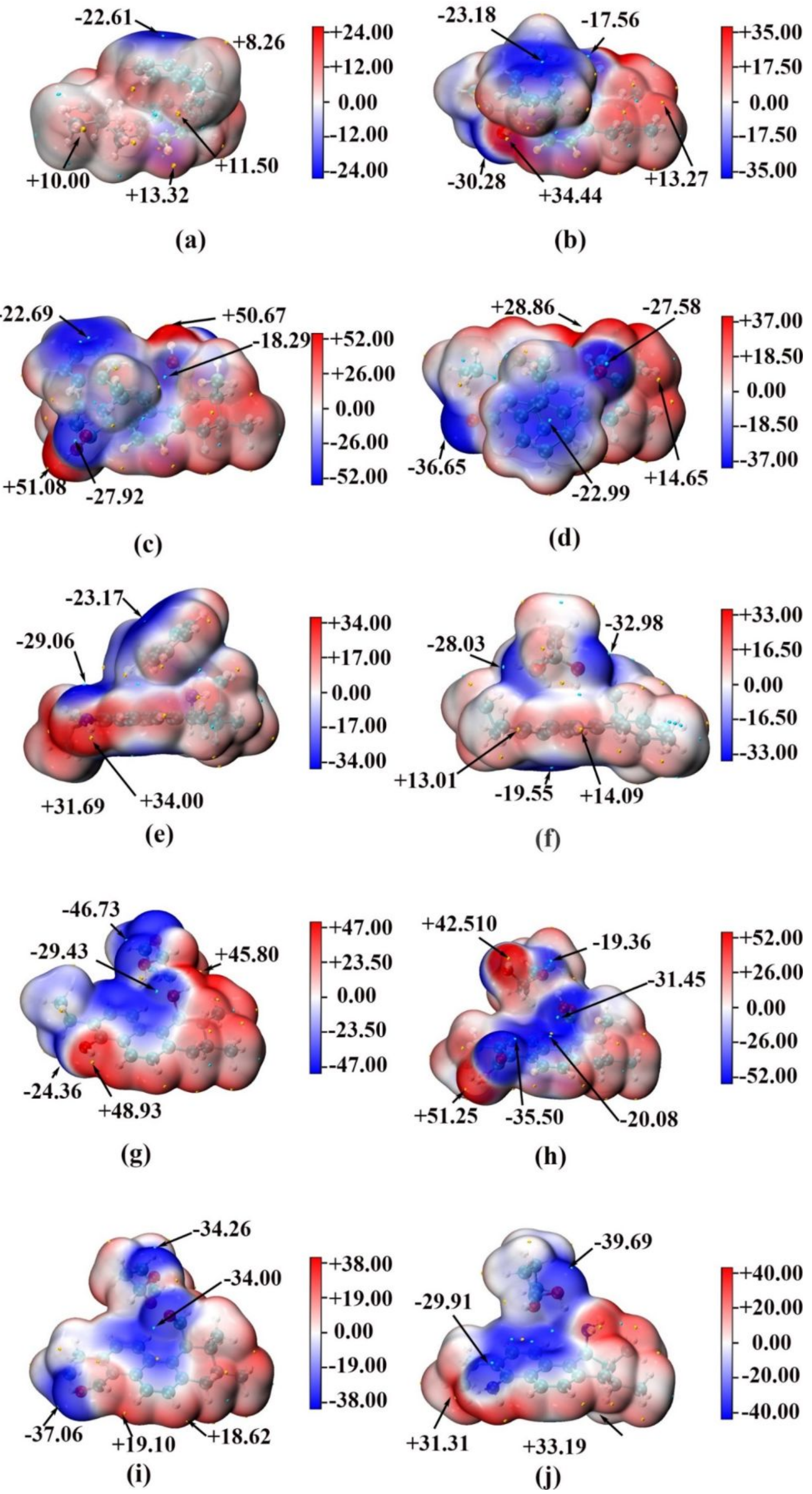

Figure S1. The overall electrostatic potential diagrams of coal molecules containing different functional groups and small molecular organic matter. (a)~(e) are the electrostatic potential diagrams of Met adsorbed by coal molecules containing $-CH_3$, -OH, -COOH, -C=O and-NH_2, respectively. (f)~(j) are the electrostatic potential diagrams of Tet adsorbed by coal molecules containing $-CH_3$, -OH, -COOH, -C=O and-NH_2, respectively

Table S1. Adsorption energy of methane adsorbed at different positions of coal molecules

Adsorption site	Adsorption conformation	Adsorption energy
Coal-CH_3& CH_4 (1)	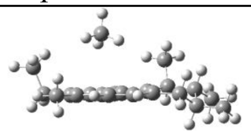	-13.04 kJ/mol
Coal-CH_3& CH_4 (2)	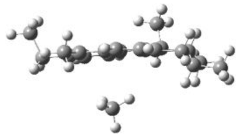	-10.79 kJ/mol
Coal-OH&CH_4 (1)	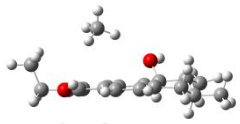	-10.71 kJ/mol
Coal-OH&CH_4 (2)	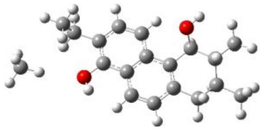	-3.92 kJ/mol
Coal-COOH&CH_4 (1)	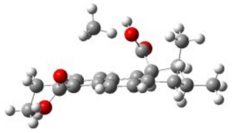	-12.51 kJ/mol
Coal-COOH&CH_4 (2)	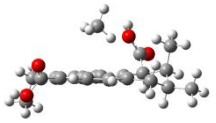	-6.70 kJ/mol
Coal-COOH&CH_4 (3)	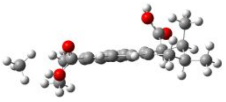	-4.58 kJ/mol
Coal-C=O&CH_4 (1)	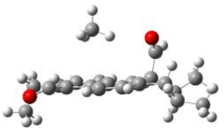	-11.84 kJ/mol
Coal-C=O&CH_4 (2)	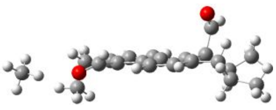	-3.89 kJ/mol
Coal-NH_2&CH_4(1)	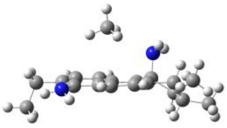	-12.78 kJ/mol
Coal-NH_2&CH_4(2)	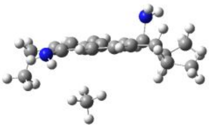	-12.33 kJ/mol
Coal-NH_2&CH_4(3)	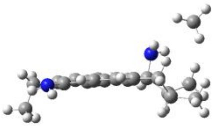	-4.06 kJ/mol

Table S2. Adsorption energy of small molecular organic matters at different adsorption sites of coal molecules

Adsorption site	Adsorption conformation	Adsorption energy
Coal-CH3&Met(1)	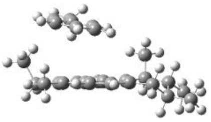	-31.20 kJ/mol
Coal-CH3&Met(2)	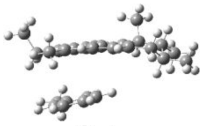	-33.60 kJ/mol
Coal-CH3&Tet(1)	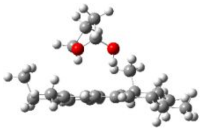	-53.61 kJ/mol
Coal-CH3&Tet(2)	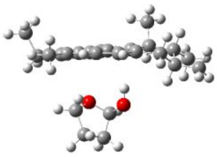	-43.52 kJ/mol
Coal-OH&Met(1)	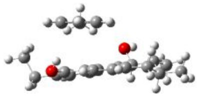	-43.96 kJ/mol
Coal-OH&Met(2)	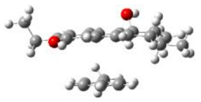	-35.97 kJ/mol
Coal-OH&Tet(1)	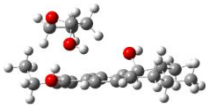	-51.77 kJ/mol
Coal-OH&Tet(2)	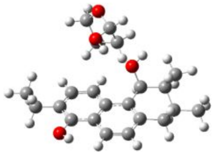	-27.40 kJ/mol
Coal-COOH&Met(1)	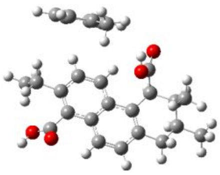	-28.64 kJ/mol
Coal-COOH&Met(2)	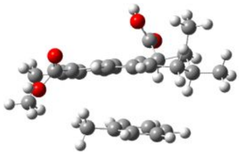	-36.08 kJ/mol
Coal-COOH&Met(3)	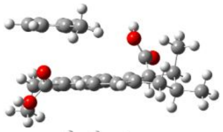	-38.69 kJ/mol
Coal-COOH&Tet(1)	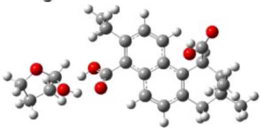	-28.64 kJ/mol

Coal-COOH&Tet(2)	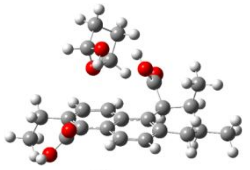	-60.63 kJ/mol
Coal-COOH&Tet(3)	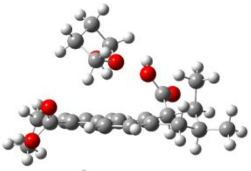	-50.45 kJ/mol
Coal-C=O&Met(1)	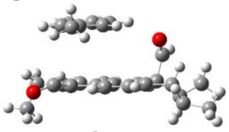	-38.04 kJ/mol
Coal-C=O&Met(2)	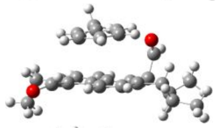	-39.26 kJ/mol
Coal-C=O&Met(3)	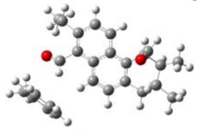	-22.97 kJ/mol
Coal-C=O&Tet(1)	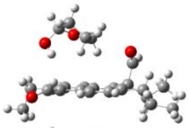	-39.12 kJ/mol
Coal-C=O&Tet(2)	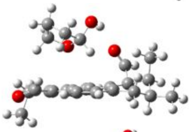	-73.14 kJ/mol
Coal-C=O&Tet(3)	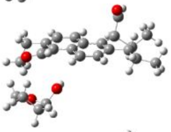	-40.36 kJ/mol
Coal-NH_2 &Met(1)	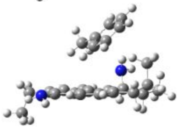	-64.55 kJ/mol
Coal-NH_2 &Met(2)	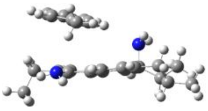	-35.70 kJ/mol
Coal-NH_2&Met(3)	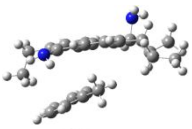	-34.25 kJ/mol
Coal-NH_2&Tet(1)	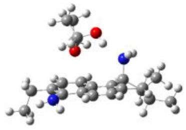	-55.64 kJ/mol
Coal-NH_2&Tet(2)	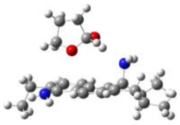	-54.87 kJ/mol
Coal-NH_2&Tet(3)	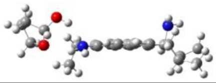	-54.49 kJ/mol

Optimized geometries and atom coordinates of all compounds

CH_4

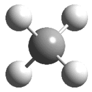

C	-1.57679739	1.094771185	0
H	-1.2137189063	0.0677905618	-0.0000009191
H	-1.2136994922	1.6082543673	0.8893874992
H	-1.2137009932	1.6082554287	-0.8893874992
H	-2.6660701683	1.0947843822	0.0000009191

Met

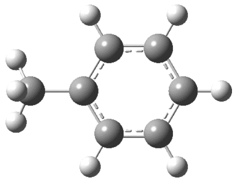

C	-1.19654431	1.20124460	0.00215371
C	0.19439193	1.19850791	-0.00902710
C	0.90962733	0.00034289	-0.01152135
C	0.19479739	-1.19824608	-0.00902667
C	-1.19598079	-1.20156513	0.00215128
C	-1.89729053	-0.00023925	0.00827674
H	-1.73333915	2.14279679	0.00196724
H	0.73444552	2.13975823	-0.01787706
H	0.73532557	-2.13925111	-0.01788799
H	-1.73244764	-2.14330456	0.00196504
H	-2.98068852	-0.00049381	0.01393687
C	2.41658226	0.00010494	0.00951285
H	2.78775850	-0.01314943	1.03817090
H	2.81768341	-0.87813236	-0.49893901
H	2.81776257	0.89087701	-0.47645274

Tet

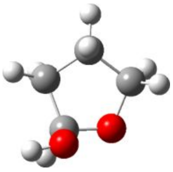

C	0.83202502	-0.09166968	0.47922756
O	-0.07094341	-1.14614318	0.44733925
C	-1.27871860	-0.73335827	-0.20598803
C	-1.14643261	0.77748087	-0.45347130
C	-0.03132047	1.16720688	0.52193478
H	1.47628776	-0.23073627	1.35179492
H	-1.39624625	-1.29913344	-1.13145227
H	-2.10858814	-0.97132983	0.46337113
H	-0.82619986	0.96857604	-1.47777748
H	-2.07964303	1.30983511	-0.27312506
H	0.51440428	2.06477448	0.22964729
H	-0.42025000	1.30142871	1.53444310
O	1.60215274	-0.14512245	-0.71153901
H	2.33724054	0.46875150	-0.63352166

Coal-CH_3

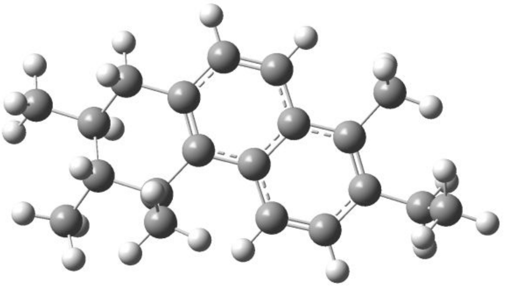

C	-3.35127835	-0.85117004	0.14203328
C	-1.83645267	-1.18138295	0.18475006
C	0.50143187	-0.18182789	-0.08056479
C	1.40060342	0.92007349	-0.02116881
C	0.85905878	2.21423461	0.20471832
C	-0.48977236	2.39350056	0.31717909
C	-1.39062513	1.30203964	0.26355578
C	-0.90743217	0.02051048	0.11363820
C	-3.64644055	0.51415118	-0.48440348
C	-2.87384385	1.57048319	0.30262239
C	-4.10648331	-1.99095812	-0.54444309
C	-5.13922991	0.83523128	-0.50781099
C	2.80935880	0.72937709	-0.20393732
C	3.30013646	-0.53195298	-0.47680471
C	2.39281398	-1.61733769	-0.56538296
C	1.04952633	-1.45960859	-0.37203282
C	4.77350238	-0.82764295	-0.64790844
C	5.49166041	-0.95757430	0.70065146
H	-0.89257952	3.39295277	0.44871794
H	1.51486928	3.07302318	0.26210373
H	-1.62471160	-1.81943877	-0.68215238
H	-3.70322195	-0.78857014	1.18198615
H	-3.26492210	0.51348898	-1.51488837
H	-3.24208178	1.58268290	1.33768827
H	-3.06552677	2.56700967	-0.10691924
H	-3.78558741	-2.96202270	-0.15956758
H	-3.91242087	-1.97799028	-1.62196435
H	-5.18439432	-1.91654920	-0.39209817
H	-5.56774435	0.73868355	0.49552412
H	-5.30805596	1.86027408	-0.84675812
H	-5.68966780	0.17037425	-1.17536554
H	2.78547679	-2.60189787	-0.79965970
H	0.40276157	-2.32183163	-0.46370882
H	4.87853796	-1.76399805	-1.20165943
H	5.25592197	-0.05974981	-1.25662817
H	6.55068474	-1.18741085	0.56471601

H	5.41156367	-0.03288577	1.27672726
H	5.04052244	-1.75518833	1.29507846
C	-1.55154977	-1.98524628	1.46725513
H	-0.52414138	-2.34458902	1.51975929
H	-1.72364213	-1.34619290	2.33751417
H	-2.22218186	-2.84544363	1.54094334
C	3.71827345	1.93038138	-0.09805624
H	3.62059769	2.40763411	0.88084551
H	4.76430636	1.66550891	-0.23077113
H	3.46609037	2.68043996	-0.85280357

Coal-OH

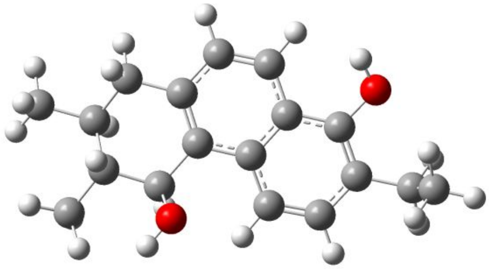

C	-3.30658469	-0.82334138	0.25124032
C	-1.82352600	-1.15738727	0.03075788
C	0.52161500	-0.18088901	-0.09924463
C	1.40959400	0.93041969	-0.03378874
C	0.86699851	2.23142377	0.13541333
C	-0.48148802	2.41460153	0.22423882
C	-1.38011168	1.31844124	0.17873594
C	-0.88926357	0.03864256	0.05421657
C	-3.68643863	0.49681504	-0.42453065
C	-2.86051476	1.60702368	0.21758568
C	-4.18019987	-1.99315415	-0.20593915
C	-5.17816479	0.80752324	-0.32385514
C	2.81029619	0.71021042	-0.17032727
C	3.32525445	-0.53653675	-0.44078522
C	2.42054368	-1.61481181	-0.54291415
C	1.07104544	-1.46198644	-0.36218821
C	4.81211465	-0.74382653	-0.56795580
C	5.49185737	-0.83607641	0.80349569
O	3.70382700	1.74119336	-0.06898496
O	-1.37671833	-2.05991263	1.04284673
H	-0.88671595	3.41677759	0.31713371
H	1.50573854	3.10837840	0.14780690
H	-1.72179346	-1.64442651	-0.95343446
H	-3.43090426	-0.68592815	1.33412402
H	-3.40862439	0.43229750	-1.48624899
H	-3.18978465	1.73872475	1.25720639
H	-3.04885666	2.55848398	-0.28936235
H	-3.82849846	-2.95049222	0.18994698
H	-4.17786748	-2.06655093	-1.29820320
H	-5.21303264	-1.87756223	0.12466997
H	-5.50914808	0.76257518	0.71902197
H	-5.38547375	1.81299279	-0.69793450
H	-5.78307107	0.10734534	-0.90202452
H	2.82279183	-2.60020586	-0.75666649
H	0.42398344	-2.32482635	-0.40684369
H	4.99320512	-1.66122277	-1.13406344

H	5.24986713	0.08071610	-1.13484804
H	6.56736891	-0.99397369	0.69811116
H	5.33314812	0.08599325	1.36526102
H	5.07754534	-1.66402802	1.38311397
H	3.29000043	2.49938050	0.34819287
H	-1.95491117	-2.82724306	1.03921502

Coal-COOH

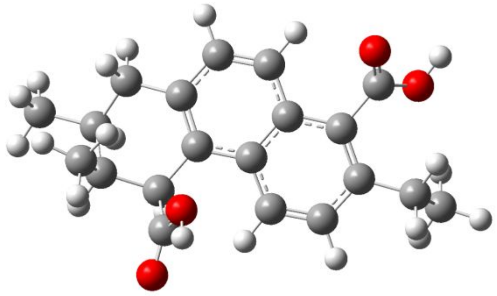

C	3.08524158	0.96955908	0.06619529
C	2.69402713	-0.33406244	-0.15856700
C	1.31272767	-0.71129100	-0.17889779
C	0.32163319	0.27321769	0.09634186
C	0.75071006	1.61167587	0.31205775
C	2.07655510	1.93897776	0.29011132
C	0.89065168	-2.04019831	-0.44966734
C	-1.06006354	-0.09974149	0.11671630
C	-0.43811324	-2.35857509	-0.44999825
H	1.62444478	-2.80118633	-0.67553789
H	0.02541295	2.40052355	0.46302106
H	2.37608265	2.97178762	0.43604918
H	-0.74686642	-3.37686518	-0.66190599
C	-4.93997078	-1.65672187	1.33627349
H	-5.58235340	-1.55135575	0.45843780
H	-4.88178324	-2.71927726	1.58149972
H	-5.42343227	-1.14520039	2.17321999
C	-4.71845570	0.96867310	0.15241608
H	-4.82067465	0.49038936	-0.82437500
H	-5.63436327	0.79013347	0.71571437
H	-4.63649512	2.04610256	-0.00527509
C	-2.29106490	1.76625123	-0.90027463
C	-3.50548354	0.43074096	0.92822528
H	-3.58649310	0.83748059	1.94035826
C	-3.53977830	-1.10484548	1.07733405
H	-2.91538072	-1.36041645	1.94152202
C	-2.88728071	-1.76140539	-0.13979720
H	-3.38516970	-1.42317637	-1.05829490
H	-2.99723030	-2.84817268	-0.09275894
C	-1.43125612	-1.39134310	-0.17426812
C	-2.12946943	0.95344646	0.37384334
H	-1.75327994	1.67464797	1.09716162
C	3.72970205	-1.37891314	-0.42827556
O	3.75270457	-2.13083010	-1.36383256
O	4.68223800	-1.41457457	0.52856543

H	5.31659318	-2.09723033	0.26811891
C	4.51938959	1.44672405	0.07105028
H	5.16368937	0.75784400	-0.47541403
H	4.55436473	2.40084582	-0.46256809
C	5.05202740	1.63955372	1.49554417
H	6.07389435	2.02378159	1.47727621
H	4.43195135	2.35027696	2.04690034
H	5.04481985	0.69067849	2.03250087
O	-2.16546303	2.95792177	-0.97783570
O	-2.58818497	1.00344149	-1.96925246
H	-2.64846266	1.59038422	-2.73498797

Coal-C=O

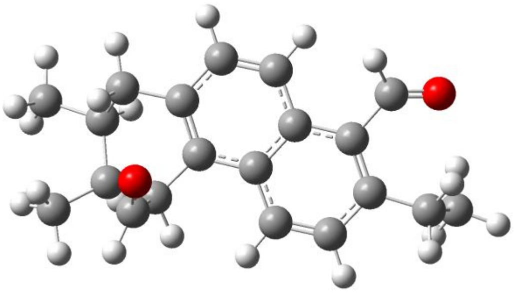

C	-1.44018812	-1.23288669	-0.21763127
C	-0.97933831	0.05457073	-0.06044869
C	-0.52014324	-2.29565966	-0.33489660
C	0.42365396	0.32130895	-0.06662351
C	1.34799088	-0.75369922	-0.21312561
C	0.82946000	-2.07104392	-0.32511484
C	2.76004826	-0.48676128	-0.20629250
C	3.24465678	0.80161652	-0.04505768
C	2.30481802	1.85359699	0.08582213
C	0.95724129	1.63098227	0.06790962
H	-0.89657330	-3.30929264	-0.42330581
H	1.49598318	-2.92072368	-0.38270155
H	2.67829060	2.86707893	0.18991524
H	0.29475227	2.48209214	0.15451322
C	-4.92718645	-1.11090732	1.35405744
H	-5.58760363	-0.91700190	0.50529882
H	-4.97465922	-2.17927800	1.57578918
H	-5.32081490	-0.57443768	2.22103783
C	-4.51934122	1.54739004	0.34779633
H	-4.83711354	1.16431719	-0.62714467
H	-5.35643615	1.43654662	1.03666665
H	-4.32639234	2.61781279	0.24163683
C	-3.48470985	-0.71093829	1.05044731
H	-2.87366492	-1.01400167	1.90715487
C	-2.92291336	-1.47380931	-0.15634031
H	-3.40592967	-1.13397286	-1.08027264
H	-3.12861194	-2.54257036	-0.05672887
C	-3.27828278	0.81671919	0.87498763
H	-3.06907568	1.22322157	1.86830044
C	4.70732759	1.17028434	0.00780010
H	5.29970687	0.51635802	-0.63079848
H	4.81194670	2.18983938	-0.37337536
C	5.24965276	1.10245471	1.44107864
H	5.19045092	0.07977534	1.81391887
H	6.29345417	1.42101594	1.47065029

H	4.67421469	1.75226249	2.10507796
C	3.69790694	-1.60999703	-0.46917513
O	4.82565475	-1.68534531	-0.04621299
H	3.31495155	-2.40490384	-1.13298058
C	-2.41864827	1.53843557	-1.42950367
O	-2.32258794	0.82816520	-2.39019790
H	-2.82599661	2.56275740	-1.53770328
C	-2.01200508	1.15365660	-0.00188562
H	-1.59541226	2.06866671	0.42351559

Coal-NH_2

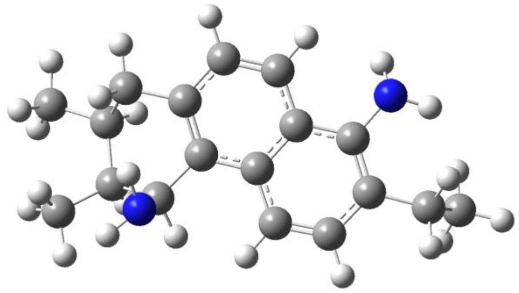

C	1.38044207	1.20049740	-0.35039250
C	0.86008164	-0.06572250	-0.19223345
C	0.51421239	2.31077785	-0.46359312
C	-0.55371409	-0.27042549	-0.18286316
C	-1.41544840	0.86203684	-0.23849939
C	-0.84336492	2.14936082	-0.39868243
C	-2.83536884	0.68098016	-0.16233363
C	-3.37464645	-0.59195559	-0.13956350
C	-2.49869914	-1.70109162	-0.15112133
C	-1.13794848	-1.56386222	-0.15611405
H	0.93801116	3.29976993	-0.60319362
H	-1.48016213	3.01764355	-0.51510574
H	-2.93488268	-2.69550131	-0.15413341
H	-0.51151533	-2.44386649	-0.18903668
C	4.92001342	0.89383330	1.08684955
H	5.53294492	0.63396964	0.21990000
H	5.04876853	1.96191868	1.27800242
H	5.31149831	0.35214358	1.95143702
C	4.30332020	-1.75802981	0.17806907
H	4.65434372	-1.40912403	-0.79709191
H	5.14542734	-1.71545504	0.86941627
H	4.02031009	-2.80996142	0.07780363
N	2.02794963	-1.62673492	-1.56184241
H	2.29892088	-0.83621005	-2.13620012
H	2.73615771	-2.34450508	-1.65994429
N	-3.66023016	1.81389999	-0.18218911
H	-3.28382899	2.61461354	0.30358995
H	-4.61029713	1.64015148	0.10819020
C	2.87711280	1.37604632	-0.33050851
H	3.33673851	1.02962094	-1.26592946
H	3.12905794	2.43545793	-0.23172386
C	1.83391999	-1.22759392	-0.16054282
H	1.36254411	-2.08266328	0.32710819
C	3.12306106	-0.93031166	0.69265682
H	2.89658813	-1.30323804	1.69699749

C	3.44372371	0.58111561	0.84998267
H	2.89212180	0.93084588	1.72936416
C	-4.86505352	-0.81871251	-0.06345398
H	-5.39390052	-0.11435341	-0.71384891
H	-5.08870785	-1.81219371	-0.45949649
C	-5.39777691	-0.72061690	1.37318018
H	-5.17065219	0.25116293	1.81951435
H	-6.47944341	-0.86782410	1.40725987
H	-4.92727858	-1.48051399	2.00032296

Coal-CH_3&CH_4

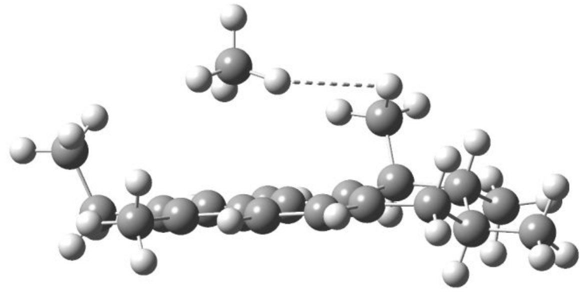

C	-3.44981632	-0.85617259	0.07572778
C	-1.93338528	-1.17860341	0.02515871
C	0.37955087	-0.16437679	-0.36245498
C	1.27991071	0.93644010	-0.31334417
C	0.74893322	2.22361387	-0.03232800
C	-0.59278720	2.39686649	0.15404928
C	-1.49318566	1.30429160	0.12245071
C	-1.01622959	0.02863658	-0.08780239
C	-3.78661853	0.51947557	-0.50562857
C	-2.97254674	1.56437937	0.25477424
C	-4.23831327	-1.98753272	-0.58655460
C	-5.27936072	0.83346744	-0.43380929
C	2.68491570	0.74461125	-0.51720452
C	3.17026761	-0.51672616	-0.79956683
C	2.25823158	-1.59732975	-0.89864112
C	0.91743180	-1.43775833	-0.68786796
C	4.64385944	-0.82081477	-0.94817003
C	5.33141649	-0.94792870	0.41672869
H	-0.98944147	3.39143494	0.33295210
H	1.40637446	3.08156354	0.01884426
H	-1.76982104	-1.80922834	-0.85728151
H	-3.74121089	-0.81485613	1.13522444
H	-3.46726161	0.54059549	-1.55680065
H	-3.27837483	1.55473349	1.30997828
H	-3.19184798	2.56775753	-0.12296219
H	-3.89279743	-2.96311881	-0.23569540
H	-4.10457871	-1.95647924	-1.67284023
H	-5.30629957	-1.92042315	-0.37310426
H	-5.64707188	0.71436145	0.59097792
H	-5.47207016	1.86428155	-0.74101107
H	-5.86595690	0.17947811	-1.08098216
H	2.64558849	-2.58032897	-1.14779958
H	0.26658694	-2.29710629	-0.77890021
H	4.75394778	-1.76036626	-1.49542462
H	5.14246171	-0.05813954	-1.55030211

H	6.39207307	-1.18419129	0.30688266
H	5.24199194	-0.01988830	0.98637897
H	4.86133184	-1.73947183	1.00495911
C	-1.56703140	-1.98855152	1.28328730
H	-0.53284103	-2.33311647	1.27676095
H	-1.69891087	-1.35860475	2.16736944
H	-2.22128587	-2.85841550	1.38671218
C	3.59924249	1.93902301	-0.39130239
H	3.53055336	2.37619613	0.60940427
H	4.64041240	1.67904414	-0.56548555
H	3.32676271	2.71948089	-1.10673774
C	1.68623804	-0.09968271	2.75747717
H	2.38664792	0.72678518	2.63217320
H	1.75386313	-0.48656662	3.77425451
H	0.67478001	0.25816781	2.56084178
H	1.93205517	-0.88554556	2.04173891

Coal-CH_3&Met

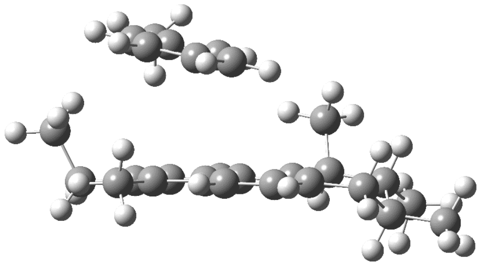

C	-4.05060855	-0.80494891	0.40594006
C	-2.55702416	-1.19172106	0.23825844
C	-0.32662218	-0.42623027	-0.76015813
C	0.54753247	0.59771789	-1.22218315
C	0.05352813	1.92874952	-1.27300721
C	-1.23449078	2.20682775	-0.91875254
C	-2.11100485	1.19427985	-0.45570067
C	-1.66212446	-0.10341637	-0.33729461
C	-4.47147474	0.36546784	-0.48593973
C	-3.55130821	1.54468785	-0.17864911
C	-4.92653074	-2.04321333	0.20493153
C	-5.93643717	0.75025044	-0.29054927
C	1.88402870	0.29329706	-1.63666340
C	2.32444289	-1.01641051	-1.62449046
C	1.44013108	-2.03084839	-1.18299198
C	0.16752766	-1.75710872	-0.76251680
C	3.71258531	-1.42403508	-2.06854888
C	4.78499712	-1.13540887	-1.01330294
H	-1.60940461	3.22282301	-0.99525366
H	0.69827299	2.73169187	-1.60555204
H	-2.52425455	-2.04441130	-0.45143788
H	-4.18512116	-0.45800525	1.44089921
H	-4.31447606	0.07817700	-1.53479980
H	-3.68925455	1.83853276	0.87114302
H	-3.82897658	2.41327084	-0.78330371
H	-4.53179893	-2.89826804	0.75913383
H	-4.95571972	-2.31688439	-0.85477933
H	-5.95105193	-1.87771457	0.54192035
H	-6.14456507	0.94175526	0.76756278
H	-6.17389429	1.65936990	-0.84836767
H	-6.61397974	-0.03424771	-0.63137233
H	1.78573513	-3.06036077	-1.19587586
H	-0.46867462	-2.57698362	-0.45691294
H	3.70509717	-2.49492116	-2.28745162
H	3.97235310	-0.92610968	-3.00625430
H	5.77169191	-1.45084958	-1.36036414

H	4.82383669	-0.07038408	-0.77604552
H	4.55973206	-1.66760676	-0.08646710
C	-2.02789770	-1.65387026	1.60820063
H	-1.03071709	-2.08920613	1.54922046
H	-1.98138560	-0.79584647	2.28544912
H	-2.69856988	-2.39565769	2.04983845
C	2.76909844	1.42373313	-2.10163572
H	2.92046426	2.14534285	-1.29346486
H	3.74780575	1.07320748	-2.42021853
H	2.31679589	1.95584206	-2.94286736
C	3.16749462	2.01804957	1.56306336
C	3.55123169	0.69447579	1.76515541
C	2.60608817	-0.28618527	2.06362384
C	1.26634265	0.09360314	2.16657009
C	0.87602414	1.40988653	1.96037908
C	1.82752719	2.37869066	1.65335814
H	3.91829082	2.76703998	1.33581074
H	4.59910730	0.42081411	1.69642699
H	0.51955069	-0.65732835	2.39988737
H	-0.17472028	1.67507841	2.01279845
H	1.52374145	3.40533232	1.48402427
C	2.99906666	-1.72748074	2.25546128
H	4.07846076	-1.83391409	2.37571150
H	2.51309879	-2.15201502	3.13662111
H	2.68978983	-2.32059864	1.38899745

Coal-CH_3&Tet

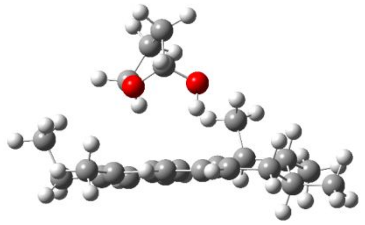

C	-3.73191692	-0.20847494	0.89911346
C	-2.24549309	-0.54412680	1.17752738
C	-0.01481616	-1.11612468	0.07257841
C	0.88751280	-1.01114783	-1.02077005
C	0.38639005	-0.52277066	-2.25816663
C	-0.93800568	-0.21321100	-2.39749913
C	-1.83461067	-0.27612395	-1.29950348
C	-1.37191291	-0.66300874	-0.06042382
C	-4.18286111	-0.63897876	-0.49938301
C	-3.28766244	0.05873868	-1.51986007
C	-4.60823407	-0.79740590	2.00618510
C	-5.65364331	-0.32442978	-0.76417381
C	2.26167800	-1.39075879	-0.87856962
C	2.69465945	-1.95393088	0.30514517
C	1.77100639	-2.11574703	1.36905381
C	0.47331173	-1.69827493	1.27385679
C	4.12902635	-2.36663228	0.54809416
C	5.00595829	-1.17698370	0.95559771
H	-1.31983661	0.10901030	-3.36150671
H	1.04940618	-0.41977719	-3.10677042
H	-2.22641817	-1.50657110	1.70445506
H	-3.83125537	0.88619093	0.92869596
H	-4.02679890	-1.72274474	-0.59464749
H	-3.44666078	1.14386321	-1.45192641
H	-3.56776660	-0.23419356	-2.53668023
H	-4.19039355	-0.58549469	2.99317344
H	-4.67381693	-1.88510658	1.89775700
H	-5.62114479	-0.39228852	1.98362973
H	-5.86055919	0.73272198	-0.56645391
H	-5.90971504	-0.52643950	-1.80716750
H	-6.31805461	-0.92153656	-0.13760664
H	2.11587838	-2.57919055	2.28828283
H	-0.18895908	-1.83848925	2.11780546
H	4.14590438	-3.11462029	1.34497543
H	4.54686998	-2.85354628	-0.33552059
H	6.03481540	-1.49027287	1.14590449
H	5.01655148	-0.41516864	0.17232620

H	4.61513539	-0.71189350	1.86408792
C	-1.66325734	0.54474590	2.10097628
H	-0.66526650	0.29560448	2.46314084
H	-1.58443422	1.48254384	1.54419663
H	-2.31227744	0.70320661	2.96620393
C	3.19828627	-1.12974227	-2.03113882
H	3.19773514	-0.06135354	-2.26608838
H	4.22056805	-1.42210976	-1.80344425
H	2.88949796	-1.67356380	-2.92807910
C	1.16691010	2.90782642	-0.71473126
O	2.12466599	1.88628953	-0.61836483
C	2.37331952	1.63709256	0.77287029
C	1.85864457	2.87424701	1.53947490
C	1.50751869	3.86294609	0.41694517
H	1.25505106	3.32575688	-1.72122838
H	1.85459575	0.72495957	1.07854118
H	3.44813070	1.48425970	0.88205105
H	0.96322340	2.62052332	2.10700426
H	2.60333711	3.27189905	2.22849411
H	0.67511868	4.52167011	0.65956973
H	2.37350391	4.46229333	0.13008967
O	-0.13332934	2.42561879	-0.47951794
H	-0.28350935	1.66824863	-1.05939629

Coal-OH&CH_4

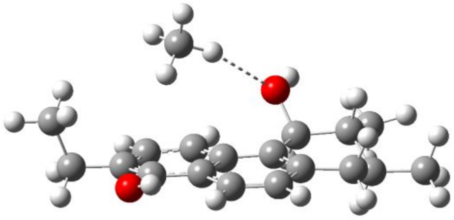

C	-3.38851475	-0.83112515	0.06524030
C	-1.91130971	-1.07834925	-0.27483561
C	0.39901838	-0.02074446	-0.35133748
C	1.26513413	1.08588164	-0.12347386
C	0.70187932	2.32513364	0.27944551
C	-0.64613617	2.45428137	0.44187808
C	-1.52131064	1.35531614	0.24874590
C	-1.00749727	0.12883185	-0.10625012
C	-3.83335432	0.56547224	-0.37719045
C	-3.00484816	1.58825534	0.39425485
C	-4.25559944	-1.94357136	-0.52710416
C	-5.32564174	0.80891007	-0.16355777
C	2.66647276	0.92351986	-0.32154213
C	3.19884859	-0.24943233	-0.80481131
C	2.31228078	-1.31340363	-1.07617049
C	0.96491818	-1.22357842	-0.84606356
C	4.68798781	-0.41132112	-0.96277462
C	5.35669661	-0.74468456	0.37652996
O	3.54187576	1.94133479	-0.05741712
O	-1.38843541	-2.10915910	0.56497542
H	-1.06930064	3.41566896	0.71369564
H	1.32301396	3.20411682	0.41644657
H	-1.84780454	-1.40612226	-1.32563158
H	-3.45992236	-0.86715800	1.16091399
H	-3.60586321	0.67587148	-1.44706718
H	-3.28822462	1.54803207	1.45474562
H	-3.23917792	2.60034026	0.04981815
H	-3.85929147	-2.93849291	-0.30338150
H	-4.30916127	-1.84636924	-1.61618171
H	-5.27207058	-1.91483849	-0.13295647
H	-5.60433918	0.59372244	0.87335742
H	-5.57587495	1.85272530	-0.36772846
H	-5.94027180	0.18669552	-0.81588203
H	2.72864337	-2.24011271	-1.45884223
H	0.33446392	-2.08205819	-1.02257157
H	4.88287063	-1.20914045	-1.68414577
H	5.11872818	0.50711454	-1.36686108

H	6.43476095	-0.87228750	0.25698101
H	5.18261781	0.05912301	1.09431112
H	4.94363238	-1.66622580	0.79329899
H	3.12624221	2.59729821	0.50601515
H	-1.93807460	-2.89096773	0.46474869
C	1.77416443	-1.64784342	2.44882672
H	0.73781965	-1.72013787	2.11917697
H	2.23829016	-0.77608187	1.98556991
H	1.81799747	-1.55419939	3.53411849
H	2.31364094	-2.54321238	2.13872692

Coal-OH&Met

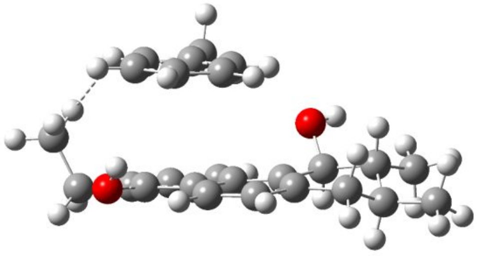

C	3.79089793	0.68183103	0.44459708
C	2.37528207	1.22476610	0.20030268
C	0.07499719	0.65934961	-0.72175716
C	-0.83312429	-0.27580319	-1.28814979
C	-0.34919518	-1.53901069	-1.71534034
C	0.97010454	-1.85406335	-1.57583149
C	1.87981668	-0.95923338	-0.95583460
C	1.43765211	0.26430801	-0.50519752
C	4.26736700	-0.17243670	-0.73358424
C	3.32868369	-1.36863834	-0.85665109
C	4.74723043	1.83159723	0.76678331
C	5.71493125	-0.63513583	-0.58382209
C	-2.20055951	0.08478429	-1.44247828
C	-2.64406464	1.36325985	-1.19855114
C	-1.70966515	2.29619745	-0.69594543
C	-0.40701119	1.96131599	-0.42979339
C	-4.08733199	1.73853576	-1.41963842
C	-4.98911446	1.29677609	-0.26280683
O	-3.10966791	-0.84164439	-1.88446568
O	1.76760677	1.56103622	1.45330909
H	1.34347766	-2.80497460	-1.94208995
H	-1.01959890	-2.24060322	-2.19822500
H	2.45401285	2.13903330	-0.41021774
H	3.71840324	0.02144460	1.32010610
H	4.18004758	0.42775126	-1.65042803
H	3.47414596	-2.02457030	0.01294865
H	3.58924010	-1.96316581	-1.73781272
H	4.34488137	2.50214108	1.53209619
H	4.93910376	2.43019642	-0.12942421
H	5.70370375	1.46606414	1.14216264
H	5.85961191	-1.13570942	0.37937635
H	5.97212684	-1.34631206	-1.37240006
H	6.41988546	0.19552410	-0.64344348
H	-2.05361153	3.30594158	-0.49212033
H	0.25448543	2.69567338	0.00691632
H	-4.15256445	2.82257597	-1.54603811

H	-4.44291735	1.28209205	-2.34588447
H	-6.02545129	1.59330060	-0.43910924
H	-4.95437391	0.21103642	-0.15430965
H	-4.65898070	1.74726380	0.67702625
H	-2.91434875	-1.68297857	-1.45685080
H	2.34162204	2.18309541	1.90889670
C	-2.90938700	-2.11727884	1.25440136
C	-2.80728102	-0.82318986	1.76176858
C	-1.57482941	-0.29642919	2.14383617
C	-0.43733070	-1.09805091	2.00724196
C	-0.53284135	-2.39000965	1.50666845
C	-1.77039439	-2.90611155	1.12541929
H	-3.88147214	-2.50940939	0.97260295
H	-3.69766652	-0.21206944	1.85993442
H	0.53105198	-0.68350023	2.26953466
H	0.36252973	-2.99084297	1.39300983
H	-1.84382433	-3.91529727	0.73550938
C	-1.46654393	1.09623277	2.70744948
H	-1.55938122	1.07696544	3.79775805
H	-0.50114681	1.53491058	2.44997128
H	-2.25623465	1.73791228	2.31123737

Coal-OH&Tet

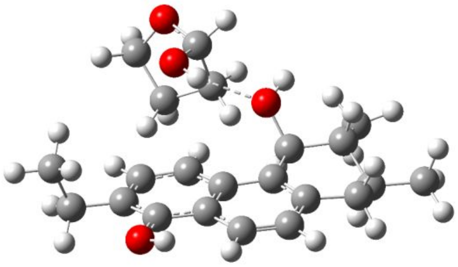

C	3.33464150	-0.21924111	-0.22184217
C	1.81876355	-0.20117739	0.01580607
C	-0.23792872	1.27483231	0.17861848
C	-0.86460370	2.54712259	0.05912996
C	-0.05049189	3.69046560	-0.15661545
C	1.30550304	3.57198824	-0.24792603
C	1.94220606	2.30656895	-0.17286078
C	1.18138892	1.17342001	-0.00209551
C	4.01181898	0.99419066	0.42075653
C	3.44854982	2.25189055	-0.23534604
C	3.91884102	-1.55063457	0.25828703
C	5.53304392	0.95284942	0.29706752
C	-2.28020860	2.63598050	0.18718970
C	-3.05233987	1.53729895	0.49225133
C	-2.39990134	0.29774817	0.65869937
C	-1.04564481	0.15748759	0.50357453
C	-4.55099507	1.65502108	0.59108277
C	-5.20988705	1.65460840	-0.79373641
O	-2.93186836	3.82797187	0.03550078
O	1.17085038	-0.98974921	-0.99663067
H	1.91913579	4.45774563	-0.37405417
H	-0.48294893	4.68442635	-0.20370537
H	1.61908191	-0.66426569	0.99580520
H	3.48019774	-0.13044650	-1.30798126
H	3.74230400	1.01224350	1.48618822
H	3.78353171	2.28891790	-1.28073778
H	3.85550286	3.14374830	0.25099458
H	3.32894642	-2.40920719	-0.07941466
H	3.93700861	-1.58231747	1.35215630
H	4.93658783	-1.69925373	-0.10392748
H	5.82961925	0.82177525	-0.74882532
H	5.97065213	1.88795650	0.65414518
H	5.96944635	0.13899003	0.87811540
H	-3.00014174	-0.57428580	0.89827842
H	-0.60031590	-0.81804074	0.63369295
H	-4.93206121	0.81950175	1.18367161

H	-4.81247313	2.57532407	1.11789499
H	-6.29596829	1.73154665	-0.71005739
H	-4.84971069	2.49998436	-1.38261414
H	-4.97001633	0.73568001	-1.33294547
H	-2.36409656	4.46443056	-0.40374217
H	1.74882485	-1.72867087	-1.21128672
C	-0.40625597	-3.77756921	-0.85857503
O	-1.19579540	-4.92441254	-0.87657680
C	-1.85451092	-5.04289584	0.38837462
C	-1.07910317	-4.14418098	1.37525267
C	0.15864190	-3.73963496	0.56323250
H	0.34935125	-3.89541563	-1.64345824
H	-2.89517251	-4.72824251	0.28724304
H	-1.82721009	-6.09760027	0.66636504
H	-1.66617283	-3.26044640	1.62696191
H	-0.82552572	-4.66385433	2.29860267
H	0.55895927	-2.76273460	0.83754688
H	0.95145436	-4.48700370	0.65252112
O	-1.23223438	-2.66845711	-1.11905443
H	-0.67557403	-1.88014327	-1.18529840

Coal-COOH&CH_4

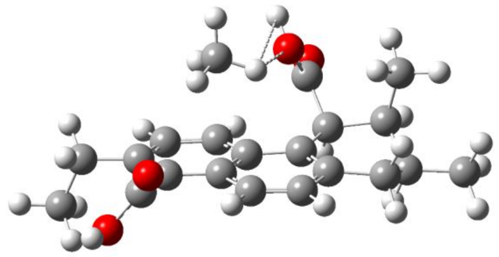

C	-2.95005614	0.90587593	-0.30851626
C	-2.55665236	-0.39196265	-0.05944072
C	-1.18095646	-0.78029640	-0.08950986
C	-0.18746933	0.19595385	-0.36682677
C	-0.62106032	1.51741262	-0.65299450
C	-1.94557202	1.85298937	-0.62275556
C	-0.76899371	-2.12273634	0.12905490
C	1.20161206	-0.16624691	-0.36467191
C	0.54890762	-2.45725505	0.03570864
H	-1.50281578	-2.88222229	0.36194143
H	0.09559859	2.28600765	-0.91610458
H	-2.24547699	2.87393702	-0.83655156
H	0.85081963	-3.49179947	0.16502936
C	5.29826592	-1.42196899	-1.21728980
H	5.72492910	-1.66623404	-0.24169475
H	5.35166144	-2.32352497	-1.83224954
H	5.92780333	-0.65921339	-1.68213181
C	4.12793569	0.33764396	1.09488210
H	3.39089245	-0.19917217	1.69671985
H	5.09482374	-0.15569109	1.20456879
H	4.22797747	1.33975814	1.51653164
C	2.09624387	2.12529585	0.32496595
C	3.71564966	0.40530427	-0.38109807
H	4.36210215	1.13368306	-0.88086905
C	3.85329908	-0.94484744	-1.09749674
H	3.45636858	-0.81043669	-2.11213781
C	2.97576932	-1.97213204	-0.39002917
H	3.40830502	-2.22100533	0.58708386
H	2.96060665	-2.90600333	-0.96095188
C	1.55427125	-1.49030091	-0.22333311
C	2.25734345	0.90972865	-0.57116126
H	2.17896740	1.30172334	-1.59066435
C	-3.57471667	-1.42289790	0.31005090
O	-3.54299920	-2.11166906	1.29414679
O	-4.56835844	-1.52411131	-0.59647129
H	-5.18352787	-2.19477134	-0.26741990

C	-4.38166817	1.38697351	-0.27077580
H	-4.99598880	0.73580386	0.35192828
H	-4.39232455	2.37384290	0.20075673
C	-4.98620224	1.48575432	-1.67604558
H	-6.00533896	1.87491891	-1.63214297
H	-4.39396277	2.15417065	-2.30539248
H	-5.00764234	0.50230981	-2.14663087
O	2.46388464	3.23042178	0.02537783
O	1.55696616	1.84312278	1.52081919
H	1.52277502	2.66656352	2.02702185
C	-0.89629503	0.10454222	2.97468552
H	-1.66676265	-0.66357522	2.89996856
H	0.05013353	-0.28906933	2.60197498
H	-1.18036958	0.96210016	2.36207439
H	-0.78343205	0.41480653	4.01394423

Coal-COOH&Met

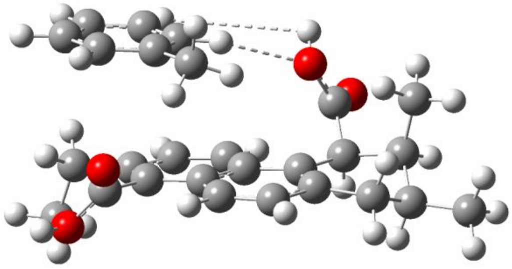

C	-2.27660657	-0.10172018	-1.40889644
C	-1.83572338	-1.09670526	-0.56022447
C	-0.45292910	-1.22261487	-0.20578054
C	0.48957355	-0.29060923	-0.72002280
C	0.01171358	0.70518796	-1.61336236
C	-1.31157510	0.78793309	-1.94021692
C	0.02903501	-2.29112924	0.59935032
C	1.88142152	-0.40386608	-0.38078906
C	1.36353536	-2.41988688	0.84389214
H	-0.66335251	-3.01247704	1.01022739
H	0.69595646	1.40958497	-2.07061049
H	-1.64843912	1.56695269	-2.61693267
H	1.72273713	-3.26179916	1.42721283
C	6.15296888	-1.31455457	-0.12993031
H	6.43093309	-1.06462882	0.89657611
H	6.37678789	-2.37334864	-0.28019301
H	6.78920086	-0.73660240	-0.80480105
C	4.50221857	1.08513378	1.00806778
H	3.72500302	0.76501240	1.70585352
H	5.47569302	0.82521576	1.42642013
H	4.45938101	2.17433884	0.94637016
C	2.49580260	2.06771056	-0.70473581
C	4.31914713	0.45112794	-0.37658809
H	4.97503847	0.97553898	-1.07882964
C	4.67516140	-1.04107876	-0.39705790
H	4.43919012	-1.41232921	-1.40281950
C	3.77597356	-1.77762044	0.58991246
H	4.05366049	-1.50944898	1.61696975
H	3.93527297	-2.85710310	0.50530278
C	2.31289381	-1.48904194	0.35074098
C	2.87284408	0.61522139	-0.92515904
H	2.92700195	0.50726927	-2.01369485
C	-2.80638087	-2.06007158	0.04270929
O	-2.86693008	-2.36089228	1.20640036
O	-3.63613473	-2.60929829	-0.86736851
H	-4.23305855	-3.19690408	-0.38321075

C	-3.71422498	0.11376421	-1.82065053
H	-4.39827038	-0.29299523	-1.07592207
H	-3.89013003	1.19273516	-1.84383814
C	-4.00836056	-0.48588730	-3.20033267
H	-5.03830934	-0.27937194	-3.49855917
H	-3.34459358	-0.05920503	-3.95633606
H	-3.85965976	-1.56588180	-3.18547898
O	2.82770688	2.96277842	-1.43530458
O	1.81057262	2.28938527	0.43123037
H	1.67814700	3.24584557	0.49819137
C	-4.21570531	1.43388875	1.53793889
C	-3.33556498	0.60904773	2.22802772
C	-1.95457001	0.81607685	2.15755297
C	-1.48003100	1.85968397	1.36598417
C	-2.35785033	2.69020249	0.67362069
C	-3.72940063	2.48392082	0.76079964
H	-5.28384100	1.25870139	1.60454782
H	-3.71570111	-0.21864982	2.81742921
H	-0.40877979	1.99705677	1.27063545
H	-1.96715741	3.49117032	0.05504962
H	-4.41572980	3.12857212	0.22346928
C	-1.00466277	-0.06884216	2.92052663
H	0.00712373	0.00283972	2.51414140
H	-0.97119782	0.22314730	3.97443538
H	-1.32684519	-1.11064720	2.86647252

Coal-COOH&Tet

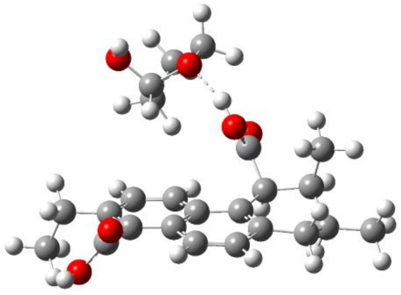

C	2.98410018	0.30737764	-0.51386556
C	2.59208338	1.45459463	0.14454996
C	1.21771562	1.84385418	0.22733395
C	0.22648882	1.02370563	-0.37354692
C	0.65960795	-0.13008846	-1.07846725
C	1.98162352	-0.47192013	-1.14049135
C	0.80472667	3.05402784	0.85039125
C	-1.16125667	1.37945626	-0.28359970
C	-0.50812985	3.42019070	0.82859986
H	1.53506020	3.69087004	1.33048862
H	-0.05902358	-0.75105744	-1.59997141
H	2.28086327	-1.36665987	-1.67861710
H	-0.80834753	4.36589265	1.26889035
C	-5.24286195	2.84522862	-0.75388492
H	-5.67837341	2.78702808	0.24633547
H	-5.28945970	3.89005111	-1.07038527
H	-5.86899289	2.25950490	-1.43155962
C	-4.10896744	0.45773152	0.92193718
H	-3.38983656	0.79363159	1.67212500
H	-5.08535533	0.88600223	1.15424431
H	-4.19771660	-0.62573103	1.02078863
C	-2.02279137	-1.00858097	-0.30694166
C	-3.67147310	0.84190324	-0.49665882
H	-4.31121359	0.30538785	-1.20490361
C	-3.79986124	2.34764708	-0.76956426
H	-3.39150733	2.52615145	-1.77290798
C	-2.93031694	3.11102491	0.22503888
H	-3.37476661	3.05557716	1.22663436
H	-2.90818557	4.17361074	-0.03796116
C	-1.51161769	2.59528950	0.25663557
C	-2.21167158	0.41476316	-0.81251438
H	-2.12277450	0.34341973	-1.90156576
C	3.61245642	2.31461409	0.82033385
O	3.57931584	2.66948497	1.96731305
O	4.61028547	2.68502721	-0.00933863

H	5.22518533	3.22071500	0.51152495
C	4.41283468	-0.17343762	-0.61832599
H	5.02366445	0.24070834	0.18443340
H	4.41200222	-1.25927304	-0.48411096
C	5.03356453	0.17280052	-1.97641803
H	6.05057107	-0.21789486	-2.04743439
H	4.44660223	-0.25780754	-2.79108838
H	5.06430826	1.25437006	-2.11138273
O	-2.31289540	-1.98204306	-0.96510439
O	-1.54659201	-1.07956765	0.92874187
H	-1.37690413	-2.02673055	1.15559285
C	0.63871198	-3.46289676	0.71923372
O	-0.65750396	-3.57124110	1.28920280
C	-1.38921638	-4.63241157	0.63274771
C	-0.53084960	-5.07006142	-0.56292313
C	0.46585941	-3.91389438	-0.71814792
H	0.94398399	-2.41945929	0.84165433
H	-1.55289963	-5.43561075	1.35160638
H	-2.34395366	-4.21090174	0.31802268
H	0.00090662	-5.99428053	-0.33643245
H	-1.13526255	-5.21852304	-1.45660504
H	1.41482841	-4.21036607	-1.16271044
H	0.02025154	-3.10167434	-1.29478161
O	1.53538189	-4.33563365	1.35002648
H	1.56785493	-4.11067397	2.28463503

Coal-C=O&CH_4

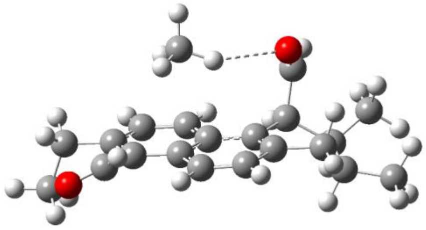

C	1.50333042	-1.22693368	0.00954904
C	1.04613074	0.06231843	-0.14521588
C	0.58160822	-2.29331387	0.07180752
C	-0.35640051	0.32759952	-0.17778385
C	-1.28269554	-0.75082589	-0.08224404
C	-0.76707583	-2.06996867	0.01507677
C	-2.69321982	-0.48245015	-0.09020907
C	-3.17605800	0.81165490	-0.20389663
C	-2.23433735	1.86660089	-0.29013277
C	-0.88671977	1.64165385	-0.27156289
H	0.95667713	-3.30780080	0.15617096
H	-1.43451028	-2.92082261	0.03363730
H	-2.60600898	2.88391535	-0.35692400
H	-0.22276833	2.49492764	-0.31748870
C	5.04970194	-1.07367507	-1.41923352
H	5.67570313	-0.90138610	-0.54028310
H	5.10521681	-2.13647174	-1.66457146
H	5.47799674	-0.51695525	-2.25655597
C	4.59982778	1.55764499	-0.35827090
H	4.86563450	1.15045541	0.62246213
H	5.47058529	1.45866231	-1.00597179
H	4.40596140	2.62633074	-0.23744733
C	3.59655381	-0.67857990	-1.16409482
H	3.01934038	-0.96295929	-2.05016244
C	2.98793063	-1.46431236	0.00448730
H	3.43205268	-1.13959163	0.95321888
H	3.20025369	-2.53078093	-0.10506267
C	3.38481808	0.84486490	-0.96475973
H	3.22764860	1.27450014	-1.95790737
C	-4.63831437	1.18318581	-0.24458215
H	-5.23120350	0.50669812	0.36973279
H	-4.74285547	2.18809143	0.17365728
C	-5.18016152	1.16770404	-1.67956940
H	-5.12265705	0.15883864	-2.08861310
H	-6.22336120	1.48905292	-1.69796709
H	-4.60335628	1.83974711	-2.31982109
C	-3.63370098	-1.61031419	0.14245783

O	-4.75745480	-1.67730877	-0.29193518
H	-3.25679675	-2.41616108	0.79712343
C	2.41206064	1.53639598	1.30343901
O	2.28506174	0.80884938	2.24806294
H	2.79770467	2.56463714	1.44623339
C	2.07584329	1.16604193	-0.14573574
H	1.67356383	2.08244632	-0.58172806
C	-1.04160717	-0.20289640	3.11284189
H	-1.52237893	0.61810577	2.57831110
H	-1.63991648	-1.10699879	2.99349569
H	-0.04483444	-0.36788132	2.70198005
H	-0.96363491	0.04470529	4.17125114

Coal-C=O&Met

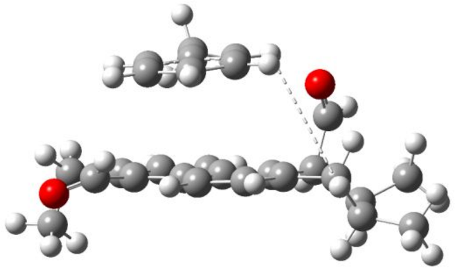

C	1.65157985	-0.31218021	-0.96485387
C	1.29770509	0.75156610	-0.16807241
C	0.66089698	-0.99811143	-1.69981695
C	-0.07795586	1.10877202	-0.02031902
C	-1.07726359	0.39248422	-0.74170648
C	-0.65794173	-0.65276832	-1.60773670
C	-2.46095639	0.74798913	-0.58508258
C	-2.84389960	1.78234828	0.25628356
C	-1.83134205	2.47304114	0.96667281
C	-0.50896275	2.15274348	0.84053158
H	0.95699899	-1.81940469	-2.34374763
H	-1.37629827	-1.20198701	-2.19996511
H	-2.12750523	3.27036967	1.64059667
H	0.21214062	2.70569990	1.42881451
C	5.31153652	0.38704032	-1.82893823
H	5.87012348	-0.14571595	-1.05543184
H	5.31871714	-0.23033442	-2.72983205
H	5.84512811	1.31295145	-2.05759239
C	4.95207028	1.65508639	0.71851539
H	5.12654222	0.67229092	1.16801530
H	5.85574362	1.93803771	0.17939299
H	4.81956079	2.37703922	1.52811230
C	3.86984233	0.65623692	-1.40188817
H	3.35647227	1.11249818	-2.25461737
C	3.11073223	-0.64328386	-1.10403658
H	3.49040414	-1.10271773	-0.18268419
H	3.26136994	-1.36332437	-1.91222369
C	3.73717893	1.65128267	-0.21778073
H	3.67095046	2.65180605	-0.65344563
C	-4.26746105	2.23683874	0.47163649
H	-4.96142639	1.40090640	0.39579934
H	-4.34171423	2.63961413	1.48572991
C	-4.66974890	3.31983618	-0.53734424
H	-4.63954501	2.91617472	-1.54944516
H	-5.68323575	3.67247813	-0.33626886
H	-3.99179862	4.17503036	-0.47967966

C	-3.48947552	-0.06668916	-1.28731290
O	-4.57712749	0.33617034	-1.62422785
H	-3.22002484	-1.11970965	-1.47218126
C	2.66358217	0.72223088	1.91331609
O	2.40892149	-0.43029403	2.13279743
H	3.12353607	1.34254395	2.70679070
C	2.39849421	1.45041769	0.59487698
H	2.08889631	2.45435475	0.89383924
C	-2.18799415	-3.57857036	-0.12815584
C	-2.22734082	-2.55794180	0.81521190
C	-1.06421540	-2.12625847	1.45761658
C	0.14278443	-2.74671299	1.13645700
C	0.18551820	-3.77471960	0.19800233
C	-0.97663255	-4.19191174	-0.44116154
H	-3.10198558	-3.89903809	-0.61656530
H	-3.17344804	-2.08066580	1.05681849
H	1.05586494	-2.40045899	1.60845752
H	1.13381690	-4.24376963	-0.04096716
H	-0.94296621	-4.98987808	-1.17383186
C	-1.11951859	-1.01893594	2.47725266
H	-1.49242208	-1.39462970	3.43467717
H	-0.12700832	-0.59700917	2.64099954
H	-1.78851309	-0.21914446	2.14887573

Coal-C=O&Tet

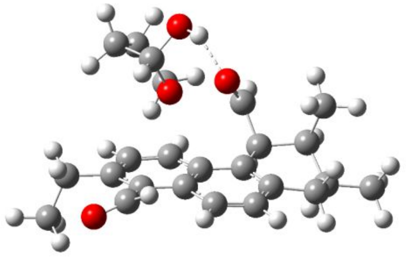

C	-1.99382382	-2.27537996	-0.02349086
C	-1.31081701	-1.09098300	0.14785947
C	-1.27288846	-3.45407213	-0.33992779
C	0.12448296	-1.09543122	0.12002708
C	0.83572430	-2.27240213	-0.23446914
C	0.08513000	-3.45791468	-0.46780105
C	2.27147323	-2.25895495	-0.27379089
C	2.98370785	-1.12897606	0.10123283
C	2.25056293	0.01069617	0.50971202
C	0.88366169	0.03932774	0.50639067
H	-1.82648852	-4.37865459	-0.46982309
H	0.58406169	-4.39102365	-0.69234579
H	2.79735972	0.89519438	0.82325946
H	0.39757939	0.95284673	0.82513998
C	-5.56449515	-1.30610948	1.12759772
H	-6.07247150	-1.38343970	0.16357403
H	-5.82552569	-2.19547326	1.70606882
H	-5.96311622	-0.43453038	1.65272317
C	-4.03737716	0.17236224	-1.18809907
H	-3.43158217	-0.48157529	-1.82047927
H	-5.08535874	-0.10919072	-1.29988741
H	-3.93147792	1.18553323	-1.58051624
C	-4.05036859	-1.20579506	0.96287540
H	-3.59868395	-1.21747141	1.96313358
C	-3.48545865	-2.38976480	0.18487930
H	-3.99931734	-2.47816442	-0.78050300
H	-3.69104269	-3.32015148	0.72353375
C	-3.59649974	0.09274485	0.28104847
H	-4.03839432	0.93713601	0.82156240
C	4.48956884	-1.03211294	0.12620587
H	4.93235450	-1.62453978	-0.67344172
H	4.76248021	0.01315497	-0.04337813
C	5.05842956	-1.49628458	1.47302950
H	4.82781040	-2.54977225	1.63226276
H	6.14312666	-1.37372717	1.49299108
H	4.63252006	-0.91535082	2.29484577

C	2.97608185	-3.45442600	-0.80249763
O	4.10123116	-3.78578131	-0.51614563
H	2.41052927	-4.04889616	-1.54192222
C	-1.64544993	1.35921208	-0.46550316
O	-1.03920558	1.26381052	-1.50259154
H	-2.00610473	2.34660195	-0.11971045
C	-2.04960204	0.20613526	0.42664800
H	-1.84200597	0.53255619	1.45511243
C	1.00774837	3.44111734	-0.66546919
O	0.35787423	3.21613206	0.57880843
C	0.24891225	4.45736263	1.28982239
C	1.08222481	5.48535484	0.50667563
C	1.95570500	4.59477763	-0.38448424
H	1.48812275	2.49615407	-0.93174917
H	-0.80259679	4.74555116	1.35965888
H	0.63549876	4.29146956	2.29807326
H	0.43637688	6.10602874	-0.11447697
H	1.66152415	6.13255597	1.16443403
H	2.28395148	5.07913686	-1.30209542
H	2.82468567	4.22325004	0.16329989
O	0.10595358	3.83943521	-1.65462176
H	-0.39044065	3.05311641	-1.92376836

Coal-NH_2&CH_4

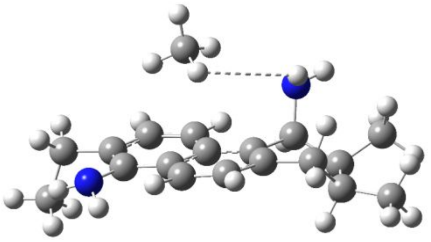

C	-1.43236430	1.19860506	0.19769186
C	-0.91341144	-0.05522030	-0.04249046
C	-0.56641719	2.30647869	0.33529911
C	0.49968714	-0.25321045	-0.09971087
C	1.35980333	0.87814378	-0.01188640
C	0.78920309	2.15474688	0.21970043
C	2.77949683	0.70183789	-0.09940225
C	3.32018027	-0.56968141	-0.15763567
C	2.44535402	-1.67963881	-0.18216421
C	1.08436126	-1.54412297	-0.17525335
H	-0.98865918	3.28557664	0.53609090
H	1.42635934	3.01929265	0.35953588
H	2.88280831	-2.67315809	-0.20789124
H	0.45871172	-2.42549349	-0.16638143
C	-5.02266025	0.94287560	-1.11795172
H	-5.60164969	0.64331312	-0.24055505
H	-5.16048830	2.01783140	-1.25711912
H	-5.44561104	0.43801265	-1.99000716
C	-4.36505871	-1.74218652	-0.34385568
H	-4.66968397	-1.43780891	0.66138585
H	-5.23841656	-1.66927156	-0.99252296
H	-4.07956327	-2.79744450	-0.30462946
N	-2.00931324	-1.70044501	1.28625116
H	-2.24911439	-0.94325733	1.91746003
H	-2.71406295	-2.42251239	1.37777538
N	3.60351652	1.83359008	-0.04384381
H	3.22345283	2.65195778	-0.49606724
H	4.55226049	1.67184368	-0.34523233
C	-2.92919804	1.36628566	0.24322303
H	-3.34963649	0.97255947	1.17843923
H	-3.18911773	2.42768776	0.20545868
C	-1.88119246	-1.22163274	-0.09791532
H	-1.42450815	-2.04294676	-0.65328764
C	-3.20955131	-0.89068671	-0.87561613
H	-3.03070084	-1.21822329	-1.90505189
C	-3.53795421	0.62514482	-0.95101378
H	-3.02024923	1.01686616	-1.83332189

C	4.81114076	-0.79204522	-0.23484832
H	5.33612384	-0.10471886	0.43653794
H	5.03511646	-1.79570784	0.13438977
C	5.34881547	-0.65319902	-1.66630066
H	5.12189972	0.33030410	-2.08631423
H	6.43081795	-0.79777576	-1.70039507
H	4.88191720	-1.39597666	-2.31621680
C	0.81668269	-0.23276468	3.13766480
H	0.31599218	0.68150351	2.81331266
H	0.25100855	-1.09479788	2.78378236
H	1.81713509	-0.25958969	2.70312026
H	0.88693330	-0.24845888	4.22564670

Coal-NH$_2$&Met

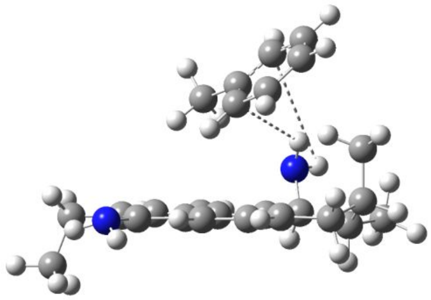

C	-0.86480037	1.01243451	1.54618725
C	-0.33714153	1.24910969	0.29458167
C	-0.07319512	0.36009225	2.52626553
C	1.01373557	0.85447322	-0.00058850
C	1.79757138	0.21645273	1.00072576
C	1.20919340	-0.02847200	2.26976891
C	3.14804227	-0.16910463	0.71801788
C	3.68075384	0.03606615	-0.54251135
C	2.88646982	0.68003268	-1.51628122
C	1.60467315	1.09063370	-1.26912510
H	-0.51406484	0.15838511	3.49766594
H	1.75730730	-0.56064752	3.03772906
H	3.31898206	0.84613546	-2.49857093
H	1.02058930	1.54082256	-2.05806405
C	-3.69684056	0.40816494	0.18290979
H	-2.88762588	-0.31392475	0.04946623
H	-4.41027962	-0.05066965	0.87245681
H	-4.20308757	0.54417157	-0.77552623
C	-3.30310961	3.21489199	-1.29728670
H	-3.79705820	2.43906623	-1.88687690
H	-4.08556716	3.83621432	-0.85550385
H	-2.72555353	3.84229025	-1.98301413
N	-1.36912007	1.02716553	-1.91186518
H	-1.83199114	0.17722422	-1.60735351
H	-1.95277106	1.45878118	-2.62013043
N	3.89474929	-0.80827030	1.71820776
H	3.70605071	-0.48521232	2.65537247
H	4.88856375	-0.82149757	1.54625838
C	-2.25789122	1.43903631	1.95777465
H	-2.69000787	0.66015571	2.59523711
H	-2.16850443	2.33763820	2.58086969
C	-1.15274250	1.94765323	-0.78631412
H	-0.52980641	2.75996572	-1.18365693
C	-2.41157221	2.62753275	-0.20609422
H	-2.03706198	3.46953234	0.39260009

C	-3.17753367	1.72403648	0.77193217
H	-4.03926478	2.29677845	1.13405935
C	5.10107041	-0.35249175	-0.87477804
H	5.34737727	-1.32248690	-0.43088354
H	5.18219099	-0.49175939	-1.95544252
C	6.11530252	0.71080294	-0.42952122
H	6.04924823	0.90334812	0.64459173
H	7.13835576	0.40577627	-0.65985931
H	5.91529950	1.65583487	-0.93846638
C	-1.88705410	-2.68321850	1.18220926
C	-0.79074128	-2.34417224	0.39268882
C	-0.87607873	-2.35625469	-0.99952334
C	-2.10092848	-2.69974088	-1.58274680
C	-3.19763375	-3.04372356	-0.79928043
C	-3.09229192	-3.04333680	0.58959498
H	-1.79347912	-2.66674948	2.26267317
H	0.14715402	-2.06636989	0.86102474
H	-2.18692381	-2.71095745	-2.66537486
H	-4.13429962	-3.31576480	-1.27256891
H	-3.94500080	-3.31348061	1.20150867
C	0.31588510	-2.01405305	-1.85507037
H	1.21413898	-1.89080401	-1.24635593
H	0.49901056	-2.80088408	-2.59128466
H	0.14982042	-1.07425145	-2.38818993

Coal-NH_2&Tet

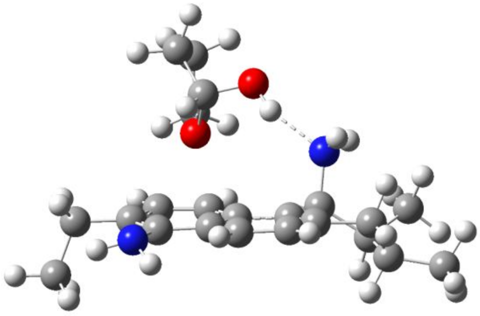

C	2.50679968	1.17908571	-0.70109732
C	1.52038939	0.50041704	-0.01931290
C	2.23018130	2.44525473	-1.26450198
C	0.20610886	1.04870011	0.09263618
C	-0.04729164	2.34633596	-0.43849360
C	0.99320237	3.01595911	-1.13106511
C	-1.34705881	2.93154761	-0.29299128
C	-2.38146928	2.20058732	0.26437101
C	-2.11695322	0.89303119	0.72916694
C	-0.87019255	0.33165787	0.67383063
H	3.01192429	2.96310777	-1.81020061
H	0.80571512	3.97352724	-1.60124607
H	-2.93323554	0.30647687	1.14051642
H	-0.74415621	-0.67989831	1.03364168
C	5.74006738	-0.33027504	0.69912995
H	5.97228274	-1.14253435	0.00551584
H	6.37433177	0.52073701	0.43997444
H	6.01613862	-0.65594849	1.70508504
C	3.81372534	-2.44539543	0.94264423
H	4.06812845	-2.67550895	-0.09603665
H	4.71136540	-2.59585654	1.54269505
H	3.07398208	-3.17674987	1.28125156
N	1.52275811	-1.80416062	-0.64204266
H	1.96324357	-1.48618706	-1.49997204
H	1.85011403	-2.74837607	-0.46341496
N	-1.56389853	4.22317399	-0.79253881
H	-0.77784548	4.84843415	-0.69251843
H	-2.40714142	4.66140607	-0.45467953
C	3.88576552	0.57464236	-0.77156271
H	3.92728476	-0.25705614	-1.48829712
H	4.60464221	1.32139065	-1.11937890
C	1.84074246	-0.89478564	0.47923865
H	1.15111856	-1.16741587	1.27898033
C	3.27776917	-1.02014919	1.10289561
H	3.13723893	-0.86113561	2.17684478

C	4.26929077	0.07492995	0.62478979
H	4.13406129	0.93430680	1.29026042
C	-3.76491907	2.78353016	0.42210916
H	-4.04312909	3.35882262	-0.46704527
H	-4.48432621	1.96355659	0.48323063
C	-3.89007857	3.65392594	1.68077099
H	-3.16535336	4.47246905	1.67623773
H	-4.89025881	4.08357486	1.76931377
H	-3.69291117	3.05436230	2.57163250
C	-2.26096489	-2.35554562	-0.82010916
O	-2.40411902	-2.38333937	0.59122050
C	-2.92926802	-3.65509191	0.98590699
C	-3.37267233	-4.37075150	-0.30296068
C	-3.39988464	-3.22817227	-1.32503847
H	-2.32293948	-1.30389710	-1.10959104
H	-2.16265195	-4.21719704	1.52426382
H	-3.76549253	-3.46782618	1.66409158
H	-2.63191864	-5.11281752	-0.60098629
H	-4.33663440	-4.86645163	-0.18927153
H	-3.23547255	-3.55320537	-2.35081673
H	-4.33651372	-2.66955745	-1.26454853
O	-1.05825880	-2.92636101	-1.23267813
H	-0.30432085	-2.35667379	-0.98394211

Met&CH_4

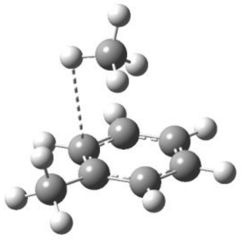

C	-1.37358675	-0.07399459	-1.20363104
C	-0.07340386	-0.56771577	-1.20157517
C	0.59450299	-0.82499082	-0.00371798
C	-0.07891963	-0.58873557	1.19528425
C	-1.37923465	-0.09509443	1.19988676
C	-2.03074229	0.16760769	-0.00115828
H	-1.87412050	0.12192480	-2.14479695
H	0.43469174	-0.75012140	-2.14317309
H	0.42439531	-0.78828040	2.13595128
H	-1.88424043	0.08403994	2.14200698
H	-3.04366266	0.55211302	-0.00015625
C	2.02416143	-1.29888023	-0.00495402
H	2.70700272	-0.44356881	-0.00442977
H	2.24330908	-1.89954153	0.87916792
H	2.24240957	-1.89748716	-0.89073124
C	1.26531472	2.47264765	0.01455270
H	1.39438616	3.55460907	0.02567653
H	1.72464912	2.05919467	-0.88335389
H	1.73834582	2.03934440	0.89583337
H	0.20428234	2.22270973	0.01988176

Tet&CH_4

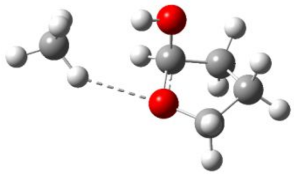

C	0.03308864	0.50892208	0.41019004
O	-0.00406015	-0.89286456	0.30779148
C	-1.27558367	-1.30550463	-0.21490606
C	-2.13246818	-0.03607746	-0.33115403
C	-1.41099877	0.93474839	0.61078762
H	0.70515005	0.74586267	1.23926877
H	-1.12638334	-1.79969344	-1.17641481
H	-1.70005806	-2.02569912	0.48865047
H	-2.10586296	0.35206013	-1.34942533
H	-3.17036701	-0.21225175	-0.05043617
H	-1.56248826	1.98394995	0.36352889
H	-1.69571019	0.76019144	1.65051328
O	0.49009659	1.09887672	-0.78282791
H	1.35150130	0.71922017	-0.98363144
C	3.41070594	-0.27828995	0.07667875
H	3.59827169	-0.21036112	-0.99592171
H	3.25514090	0.72117830	0.48540156
H	2.53391524	-0.89988870	0.26259179
H	4.28013526	-0.72545632	0.55658815

Methane and small molecular organic matters are on both sides of coal molecules.

Coal-CH_3&Met&CH_4

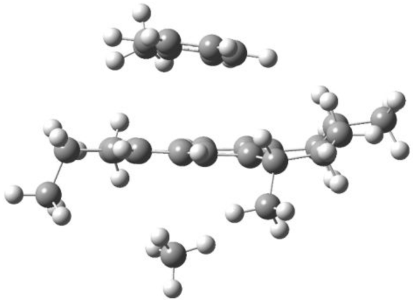

C	3.68086800	0.42473600	-0.84509200
C	2.17558500	0.49013100	-1.21551200
C	-0.17943100	0.61790000	-0.24503200
C	-1.08643400	0.99592100	0.78290400
C	-0.55061200	1.51489200	1.99204900
C	0.80083400	1.61161000	2.16945900
C	1.70977200	1.24775100	1.14571500
C	1.23297100	0.79328800	-0.06449700
C	3.92588100	0.19948300	0.65001600
C	3.19127300	1.29442100	1.42143000
C	4.37688000	-0.61817100	-1.72177800
C	5.41215000	0.18625300	1.00081900
C	-2.49957000	0.84192300	0.60791300
C	-2.98536100	0.26423100	-0.54769600
C	-2.06954400	-0.14978600	-1.54730700
C	-0.72167100	0.02530700	-1.41553300
C	-4.46066200	0.08697300	-0.82597100
C	-5.09850100	1.38118100	-1.34569000
H	1.19757900	1.97523700	3.11232000
H	-1.21308000	1.81807300	2.79271000
H	1.90071900	-0.49558200	-1.61103400
H	4.11909800	1.40675700	-1.07473400
H	3.48453000	-0.76537400	0.93671200
H	3.61111000	2.26982800	1.13970200
H	3.35941100	1.18229300	2.49685700
H	4.09683300	-0.49507200	-2.77095100
H	4.08282300	-1.62788600	-1.41514500
H	5.46396800	-0.54697000	-1.66003800
H	5.89957000	1.09641400	0.63550800
H	5.55293400	0.14255600	2.08357300
H	5.92761400	-0.67096800	0.56480600

H	-2.45907100	-0.63215600	-2.43853800
H	-0.06914300	-0.32743200	-2.20272700
H	-4.58179700	-0.70015500	-1.57484200
H	-4.98780000	-0.25854500	0.06680400
H	-6.16082500	1.24363200	-1.55890800
H	-4.99423400	2.18738800	-0.61568400
H	-4.60187500	1.70474300	-2.26334700
C	1.98929000	1.53591500	-2.33031400
H	0.96488000	1.57260500	-2.70179300
H	2.23556500	2.52749500	-1.94037100
H	2.65618200	1.32731600	-3.17144500
C	-3.41762100	1.31897300	1.70689100
H	-3.26489400	2.38414900	1.90401600
H	-4.46637300	1.17944200	1.45551900
H	-3.22588600	0.78460400	2.64234500
C	0.98621800	-2.69479300	0.09093000
C	0.15943300	-2.24554700	1.11602000
C	-1.21058800	-2.50529200	1.09517400
C	-1.73453200	-3.21979000	0.01570300
C	-0.91382600	-3.66862800	-1.01255600
C	0.45338100	-3.40772000	-0.97774700
H	2.04824200	-2.47606400	0.12210900
H	0.57832200	-1.65795600	1.92850500
H	-2.80209400	-3.41531400	-0.02192000
H	-1.34118700	-4.21751300	-1.84402600
H	1.09668900	-3.75464900	-1.77844300
C	-2.10321500	-2.04151300	2.21685600
H	-1.65496000	-1.19580000	2.74267500
H	-3.07822900	-1.72919900	1.83541500
H	-2.26453000	-2.84559700	2.94086200
C	-1.34855300	3.73486200	-1.02762400
H	-1.37582300	4.61046400	-1.67620000
H	-0.34232400	3.59598700	-0.63084500
H	-2.04279500	3.87160400	-0.19782600
H	-1.63563700	2.84458000	-1.58878800

Coal-CH_3&Tet&CH_4

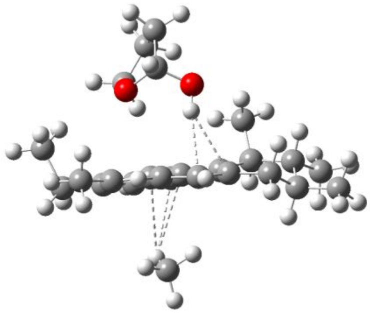

C	-3.73806413	0.21659127	0.96358504
C	-2.25301037	-0.06255287	1.30313945
C	-0.02277283	-0.83750668	0.32904676
C	0.87756727	-0.95468862	-0.76408049
C	0.38007445	-0.70576761	-2.07191631
C	-0.94310796	-0.42377463	-2.26887950
C	-1.84024889	-0.27295116	-1.17962947
C	-1.37777611	-0.41248696	0.11126072
C	-4.18838803	-0.47727924	-0.32502115
C	-3.29371164	0.00991730	-1.46144230
C	-4.61685771	-0.14471548	2.16260314
C	-5.65933753	-0.22167367	-0.64611881
C	2.23984859	-1.34400435	-0.55617369
C	2.66230354	-1.69858208	0.70957015
C	1.74139916	-1.63309224	1.78598382
C	0.45586908	-1.20007909	1.61684358
C	4.08348901	-2.10981783	1.02155231
C	5.00212797	-0.89610085	1.20448283
H	-1.32433371	-0.29309521	-3.27705850
H	1.04223690	-0.77496589	-2.92464687
H	-2.23711000	-0.90998266	2.00026756
H	-3.83500068	1.29642064	0.77991123
H	-4.03091758	-1.55896346	-0.20814847
H	-3.45455744	1.08709584	-1.60653227
H	-3.57249200	-0.47618761	-2.40174610
H	-4.20044271	0.25434886	3.09045241
H	-4.68420390	-1.23264870	2.26750052
H	-5.62902588	0.24964205	2.05967990
H	-5.86742447	0.85353861	-0.65634465
H	-5.91444837	-0.62147773	-1.63063369
H	-6.32370009	-0.68722596	0.08353686
H	2.07753457	-1.93218276	2.77407734
H	-0.20560943	-1.16645318	2.47227918
H	4.08120915	-2.70051728	1.94123664
H	4.47769123	-2.76340825	0.24064392

H	6.02040135	-1.20378496	1.45211993
H	5.03596610	-0.29277826	0.29406197
H	4.63061408	-0.25795331	2.01019581
C	-1.66852508	1.17782426	2.00773782
H	-0.67105453	0.99772306	2.40998639
H	-1.58691856	1.99527016	1.28619512
H	-2.31699822	1.49655890	2.82786080
C	3.17316979	-1.33870435	-1.74010911
H	3.19447879	-0.33929671	-2.18398744
H	4.19047390	-1.60380811	-1.46235378
H	2.84404582	-2.04398562	-2.50878115
C	1.24490051	2.92385066	-1.20013138
O	2.19433729	1.92705805	-0.92518260
C	2.45286903	1.93487886	0.48632070
C	1.96053260	3.29899862	1.01559238
C	1.60666271	4.06738946	-0.26715757
H	1.32634060	3.14815394	-2.26713190
H	1.92518674	1.10212469	0.95812540
H	3.52674962	1.79003931	0.61475031
H	1.06930928	3.16588716	1.62895961
H	2.71754052	3.80799017	1.61165352
H	0.78340098	4.76911932	-0.14283387
H	2.47522478	4.59348899	-0.66745457
O	-0.05726313	2.50818692	-0.86863932
H	-0.22245926	1.66068382	-1.30078451
C	-0.21214736	-4.02854819	-0.86313021
H	0.34419030	-3.80283166	0.04731066
H	-0.61509039	-5.03999555	-0.81225402
H	-1.02448020	-3.30868719	-0.97262044
H	0.45588312	-3.94256236	-1.72048971

Coal-OH&Met&CH_4

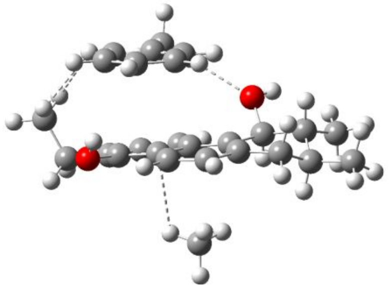

C	3.75074790	-0.22307067	0.88786276
C	2.33945168	0.31617619	1.16074130
C	0.05567063	0.74282724	0.13711080
C	-0.84439916	0.65663873	-0.95894687
C	-0.36372773	0.21037831	-2.21629505
C	0.94821520	-0.13013013	-2.37041896
C	1.85066529	-0.09959618	-1.27632004
C	1.40737508	0.29531532	-0.03404077
C	4.23518677	0.18419637	-0.50666489
C	3.29693063	-0.44563297	-1.53172477
C	4.70640886	0.21677562	1.99854656
C	5.68177602	-0.22386192	-0.77592295
C	-2.19539689	1.06720849	-0.78652490
C	-2.62171698	1.68707470	0.36466903
C	-1.69312617	1.81618057	1.42204755
C	-0.41296889	1.33255238	1.33895130
C	-4.04537649	2.16141770	0.50778552
C	-5.00246818	1.02175662	0.87127942
O	-3.10238398	0.88934589	-1.79889552
O	1.71729717	-0.45765279	2.19291041
H	1.32173194	-0.42633672	-3.34537814
H	-1.02644914	0.19023226	-3.07377588
H	2.42811924	1.35988505	1.50560279
H	3.66868029	-1.31892045	0.89999120
H	4.15177969	1.27726335	-0.59334695
H	3.43225989	-1.53597077	-1.51434962
H	3.56581229	-0.11742237	-2.54054484
H	4.29849472	0.02442928	2.99546278
H	4.90774663	1.29027359	1.92499476
H	5.65861763	-0.31191522	1.94240702
H	5.82024472	-1.29324635	-0.58478198
H	5.94437118	-0.03371259	-1.81930006
H	6.38686083	0.32632591	-0.15091266
H	-2.02456867	2.29506836	2.33870642
H	0.24319339	1.40859905	2.19416533
H	-4.08212179	2.93559930	1.27890769

H	-4.37175800	2.61958335	-0.42821970
H	-6.02472770	1.38939360	0.98414135
H	-4.99331817	0.26135099	0.08793346
H	-4.70150105	0.55095284	1.81094106
H	-2.94136520	0.02771192	-2.19948918
H	2.28798844	-0.44265304	2.96629505
C	-3.05130739	-2.36076083	-0.84441129
C	-2.93057586	-1.97769684	0.49044635
C	-1.69937859	-2.02191912	1.14178899
C	-0.58209378	-2.46013967	0.42406830
C	-0.69609082	-2.84643610	-0.90487848
C	-1.93261084	-2.79722355	-1.54711801
H	-4.02275028	-2.32801308	-1.32748988
H	-3.80514323	-1.63680547	1.03359425
H	0.38705440	-2.46442597	0.91313557
H	0.18451196	-3.16775730	-1.44970154
H	-2.02084551	-3.10101806	-2.58434314
C	-1.57000682	-1.62403803	2.58879855
H	-1.68936588	-2.49639089	3.23877799
H	-0.58757479	-1.18653909	2.77434758
H	-2.33323765	-0.89189311	2.86018920
C	0.92350564	3.63733623	-1.07884298
H	1.55239716	3.49344355	-0.19940879
H	-0.12308360	3.60432563	-0.77446966
H	1.14784840	4.60022878	-1.53755536
H	1.11265778	2.83283806	-1.79056406

Coal-OH&Tet&CH_4

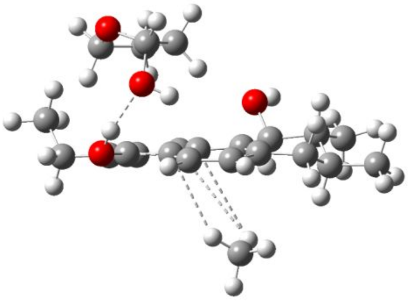

C	-3.81939897	-0.15640650	-1.17333945
C	-2.48594336	0.60248525	-1.14589142
C	-0.25614472	0.80971444	0.05089081
C	0.65798357	0.37516930	1.04581683
C	0.21511694	-0.54207871	2.03546525
C	-1.05712575	-1.03687798	1.99769062
C	-1.95707467	-0.68009761	0.95840323
C	-1.55652830	0.20544343	-0.01617399
C	-4.35829234	-0.35793734	0.24584071
C	-3.36323400	-1.22491293	1.01204410
C	-4.81489053	0.56154972	-2.08609592
C	-5.74943911	-0.98705586	0.27220372
C	1.99016030	0.88954620	1.06409990
C	2.35718429	1.93595703	0.24597874
C	1.38982820	2.43472204	-0.65987537
C	0.14722914	1.87481374	-0.79719177
C	3.74552935	2.52699186	0.27981346
C	4.45190910	2.47600756	-1.07960204
O	2.90038739	0.35044732	1.92766426
O	-1.77187444	0.38723474	-2.36345801
H	-1.40773922	-1.69556976	2.78608554
H	0.87988340	-0.79558308	2.85419458
H	-2.70464516	1.67920223	-1.04235935
H	-3.60165914	-1.14888122	-1.59136447
H	-4.40416512	0.62525211	0.73567635
H	-3.38866030	-2.24350222	0.60137092
H	-3.66750020	-1.30806105	2.06062700
H	-4.38274539	0.79947974	-3.06246486
H	-5.14235276	1.50108294	-1.62935257
H	-5.69821999	-0.04908729	-2.27623275
H	-5.76280432	-1.91252707	-0.31305090
H	-6.03753802	-1.23476013	1.29666036
H	-6.51003927	-0.31833128	-0.13349673
H	1.66408136	3.27010730	-1.29625155
H	-0.52714843	2.24581707	-1.55493204

H	3.67476828	3.56925204	0.60941629
H	4.33608152	1.99787040	1.02764886
H	5.45543352	2.90011447	-1.00818406
H	4.54316042	1.44658583	-1.43197525
H	3.90483991	3.03896533	-1.83861934
H	2.88469893	-0.61758040	1.82087769
H	-2.32925347	0.66563144	-3.09487626
C	2.57941605	-2.92018393	-0.13796792
O	3.83155880	-2.57078055	-0.61093274
C	3.73453255	-1.24504946	-1.13721404
C	2.33925527	-1.15601566	-1.77764825
C	1.56507515	-2.32238940	-1.12364345
H	2.54852236	-4.00874058	-0.05834893
H	3.85272485	-0.51965243	-0.32662332
H	4.55415900	-1.12130260	-1.84413330
H	1.86771746	-0.19168309	-1.59014172
H	2.39934319	-1.29577048	-2.85701055
H	0.65171147	-1.99203919	-0.62250193
H	1.29075219	-3.07658851	-1.86214801
O	2.42876309	-2.35937433	1.17154006
H	1.49630754	-2.41556899	1.40968090
C	-2.40827918	2.33746603	2.48021879
H	-2.65689514	2.64237134	1.46301634
H	-1.37984723	1.97676859	2.50132944
H	-2.51830066	3.18714826	3.15340386
H	-3.07656409	1.53419551	2.79263598

Coal-COOH&Met&CH_4

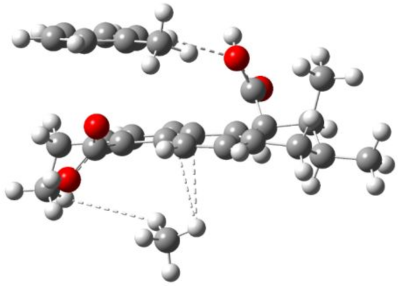

C	-2.26749831	0.47313614	1.17319868
C	-1.82298878	1.05691376	0.00508576
C	-0.44324204	1.02247375	-0.37401973
C	0.49395938	0.35091663	0.45666629
C	0.01106813	-0.23629596	1.65609824
C	-1.31068098	-0.16974802	1.99530861
C	0.04374946	1.71774202	-1.51459271
C	1.88950713	0.34373004	0.11511671
C	1.38126768	1.76427950	-1.77105846
H	-0.64636883	2.23046019	-2.17032135
H	0.69131695	-0.72558916	2.34281097
H	-1.65112499	-0.62942171	2.91788320
H	1.74567435	2.33673532	-2.61818742
C	6.15978183	1.14053596	-0.38985692
H	6.45598736	0.53698362	-1.25092687
H	6.37677291	2.18361973	-0.63204268
H	6.78693254	0.85409564	0.45800089
C	4.53204334	-1.52226183	-0.61263543
H	3.76384184	-1.47897698	-1.38817578
H	5.51056400	-1.42319700	-1.08468706
H	4.49041647	-2.51704112	-0.16461553
C	2.50176265	-1.84014662	1.31103875
C	4.32830968	-0.43506753	0.44999290
H	4.97576301	-0.66742305	1.30150848
C	4.67888333	0.96818217	-0.06246384
H	4.42530989	1.67403305	0.73919339
C	3.79315299	1.29266586	-1.26058335
H	4.08984974	0.67787155	-2.11953115
H	3.94518008	2.33266815	-1.56586991
C	2.32766985	1.09277753	-0.95522242
C	2.87478839	-0.40251899	1.00279461
H	2.91216026	0.08896198	1.98081502
C	-2.78277658	1.74467072	-0.91127564
O	-2.87295304	1.56319994	-2.09729572
O	-3.55887745	2.64720329	-0.27726739
H	-4.15261378	3.02344499	-0.94221512

C	-3.69846315	0.48672005	1.65506036
H	-4.39050449	0.60537911	0.82111432
H	-3.90948689	-0.49156511	2.09554449
C	-3.92558673	1.58569444	2.69969536
H	-4.95248081	1.56203706	3.07030168
H	-3.25404691	1.45217756	3.55149829
H	-3.73295463	2.56746202	2.26556464
O	2.82771689	-2.41435645	2.31555387
O	1.82817354	-2.45545003	0.32235631
H	1.70126916	-3.37447385	0.59823006
C	-4.25946583	-2.02955356	-0.93553089
C	-3.35249715	-1.56054324	-1.87787161
C	-1.97933222	-1.77165463	-1.71866057
C	-1.54031283	-2.45280595	-0.58558964
C	-2.44546099	-2.92781018	0.36064927
C	-3.80874708	-2.72155843	0.18750310
H	-5.32073240	-1.85545977	-1.07491838
H	-3.70398637	-1.00502492	-2.74079077
H	-0.47428115	-2.57917864	-0.43216760
H	-2.08262628	-3.44832925	1.24048077
H	-4.51611916	-3.08878738	0.92241867
C	-1.00188710	-1.27099623	-2.74906403
H	0.00549462	-1.20147890	-2.33128201
H	-0.97006851	-1.94729944	-3.60848778
H	-1.29836621	-0.28259277	-3.10560068
C	-0.08673108	3.56801924	1.67290890
H	0.79242150	3.34370549	1.06727225
H	-0.90480026	3.87369593	1.02007693
H	-0.38442756	2.67247151	2.21986122
H	0.14191714	4.37028219	2.37431439

Coal-COOH&Tet&CH_4

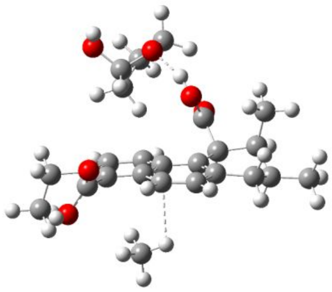

C	2.82664958	0.84643976	0.29643524
C	2.81905806	-0.32088197	-0.43685348
C	1.65803714	-1.14908229	-0.52532134
C	0.47306810	-0.75807691	0.15142621
C	0.50959973	0.43987148	0.91213144
C	1.63889449	1.20840874	0.97684841
C	1.66980679	-2.39881334	-1.20328543
C	-0.69826767	-1.58575376	0.09693074
C	0.57788713	-3.21310429	-1.15120151
H	2.55374680	-2.71235469	-1.74249801
H	-0.35721084	0.74901760	1.48379592
H	1.63581161	2.11974965	1.56776462
H	0.61045334	-4.18641626	-1.63073744
C	-3.94496836	-4.44630481	0.65748382
H	-4.43205810	-4.50989544	-0.31829470
H	-3.59453999	-5.44899957	0.91398795
H	-4.69724316	-4.15353528	1.39415629
C	-3.84396395	-1.74633743	-0.92455526
H	-3.09708299	-1.76777294	-1.72115676
H	-4.61206548	-2.49001565	-1.14292546
H	-4.32258775	-0.76578531	-0.95592146
C	-2.35856169	0.32594007	0.30966075
C	-3.21508522	-2.00221819	0.45040905
H	-3.96120904	-1.76069938	1.21441613
C	-2.77839824	-3.46202733	0.64139266
H	-2.27402697	-3.51944194	1.61487516
C	-1.75402970	-3.82086851	-0.43088078
H	-2.24797656	-3.89014134	-1.40819918
H	-1.33706660	-4.81334825	-0.23134143
C	-0.61918873	-2.82681555	-0.49272413
C	-1.98974580	-1.08989138	0.72926813
H	-1.86301877	-1.03967584	1.81583743
C	4.05088445	-0.75522117	-1.16638010
O	4.12172873	-0.98888331	-2.34192817
O	5.11704290	-0.88363484	-0.34852910
H	5.86563431	-1.15291823	-0.89970327

C	4.02997206	1.74614757	0.44809081
H	4.73173675	1.60120175	-0.37433108
H	3.68899334	2.78407258	0.39164728
C	4.73907009	1.50981120	1.78681507
H	5.58989317	2.18459553	1.89996883
H	4.05519604	1.68164529	2.62150088
H	5.09682177	0.48112996	1.84592477
O	-2.94106300	1.09489634	1.04062214
O	-2.01500885	0.62327785	-0.93625253
H	-2.21200857	1.57720405	-1.10578018
C	-0.82548425	3.62311366	-0.64139481
O	-2.10501741	3.28290418	-1.15494408
C	-3.12862856	3.97496981	-0.40309627
C	-2.41707960	4.62919027	0.79038755
C	-1.06383093	3.90701898	0.82900109
H	-0.17310462	2.76916914	-0.84671235
H	-3.60742691	4.70225660	-1.05924354
H	-3.85136644	3.22298543	-0.08644298
H	-2.26763243	5.69412193	0.61199572
H	-2.98163530	4.50016438	1.71269731
H	-0.26049269	4.50460645	1.25743561
H	-1.15251192	2.96056468	1.36476583
O	-0.34184773	4.79260936	-1.24292085
H	-0.28431673	4.64226609	-2.19118996
C	2.69381575	-2.31564021	2.36856485
H	3.58395886	-2.36738741	1.74100419
H	1.88640357	-2.87734146	1.89714344
H	2.91197610	-2.73524999	3.35045422
H	2.39221505	-1.27237518	2.47181083

Coal-C=O&Met&CH_4

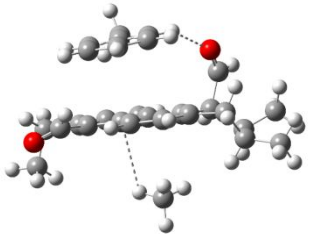

C	1.62293821	0.36334000	0.87670900
C	1.28092131	-0.55250149	-0.09082021
C	0.62626375	0.89585004	1.72198631
C	-0.08727282	-0.90801098	-0.28922301
C	-1.09194385	-0.35180397	0.55431096
C	-0.68682406	0.54302311	1.58011013
C	-2.46589101	-0.72879212	0.36965843
C	-2.83288779	-1.64064781	-0.60918446
C	-1.81518855	-2.17181514	-1.43993250
C	-0.50296326	-1.81900558	-1.29573223
H	0.91276408	1.59881545	2.49700457
H	-1.41079537	0.96421089	2.26376149
H	-2.09989934	-2.86918566	-2.22110418
H	0.22126233	-2.24489604	-1.97821240
C	5.26370135	-0.48342268	1.67125390
H	5.83950216	0.16321109	1.00450420
H	5.25663671	-0.02027891	2.66029786
H	5.78852446	-1.43821123	1.75647753
C	4.94731067	-1.32132708	-1.05368273
H	5.13803449	-0.28025300	-1.33354162
H	5.83780091	-1.69734776	-0.55066951
H	4.82580868	-1.90028452	-1.97249063
C	3.82932958	-0.66915720	1.18031569
H	3.29691689	-1.25476156	1.93756466
C	3.08002760	0.66706368	1.08279564
H	3.47370614	1.26471336	0.25114591
H	3.22175438	1.24860129	1.99705510
C	3.71417760	-1.45702413	-0.15182922
H	3.63162514	-2.51456138	0.11408153
C	-4.24284147	-2.12160155	-0.85265269
H	-4.96724276	-1.33708631	-0.63806682
H	-4.33367615	-2.37821160	-1.91182994
C	-4.56677858	-3.35449745	0.00073510
H	-4.51995174	-3.09752314	1.05912037
H	-5.57078162	-3.72123147	-0.22154950
H	-3.85594641	-4.16079764	-0.19697220
C	-3.50408757	-0.06268725	1.20232237

O	-4.56601857	-0.55189208	1.50460419
H	-3.26815111	0.96597772	1.52321283
C	2.68092260	-0.17162433	-2.11548285
O	2.39678296	0.99415321	-2.15535084
H	3.18430946	-0.64302414	-2.98148196
C	2.39331260	-1.11189804	-0.94479184
H	2.08874924	-2.04882832	-1.41742417
C	-2.28716555	3.62837480	0.58784046
C	-2.31081867	2.77241451	-0.50753213
C	-1.14333389	2.47746294	-1.21606863
C	0.05178343	3.06624705	-0.80405950
C	0.07866655	3.93010446	0.28806779
C	-1.08751785	4.21146061	0.99077792
H	-3.20428622	3.84469893	1.12517198
H	-3.24777809	2.31805738	-0.81881519
H	0.96894757	2.82268745	-1.32949888
H	1.01799229	4.37662392	0.59567135
H	-1.06613188	4.88137994	1.84249658
C	-1.18162124	1.54669101	-2.39983606
H	-1.57647874	2.05970378	-3.28169385
H	-0.18006668	1.18442746	-2.63637968
H	-1.82436767	0.68556318	-2.19804787
C	0.52027027	-3.13779604	2.02674586
H	1.31183766	-3.30045574	1.29344108
H	-0.44757464	-3.27729607	1.54415781
H	0.62997246	-3.84639846	2.84728027
H	0.58593199	-2.11664423	2.40449740

Coal-C=O&Tet&CH_4

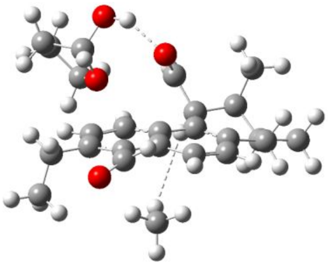

C	-1.30258898	-2.63363387	-0.29507509
C	-0.98316825	-1.31055258	-0.07911007
C	-0.28613436	-3.53822594	-0.69241433
C	0.39050693	-0.90010478	-0.13601771
C	1.39888201	-1.80860281	-0.55295567
C	1.01160770	-3.14552414	-0.84409623
C	2.77443946	-1.39648367	-0.56952871
C	3.14702255	-0.14156263	-0.10996895
C	2.12617831	0.72822694	0.34275714
C	0.80467251	0.37928301	0.31575701
H	-0.55571926	-4.57612161	-0.86088196
H	1.74990773	-3.88502500	-1.12394016
H	2.40726514	1.70841186	0.71659737
H	0.08549186	1.10207551	0.67978832
C	-4.93595041	-2.79923311	1.03847281
H	-5.45192396	-2.97095612	0.09102052
H	-4.89847485	-3.75442747	1.56753550
H	-5.53885789	-2.10873825	1.63344551
C	-4.02307780	-0.83151660	-1.23879327
H	-3.28987018	-1.25413439	-1.93039302
H	-4.95215707	-1.39598447	-1.32984346
H	-4.22983546	0.18679983	-1.57361620
C	-3.52603597	-2.25563498	0.82377403
H	-3.03582343	-2.18351992	1.80348759
C	-2.68621254	-3.18558892	-0.04577693
H	-3.20120678	-3.37039724	-0.99680200
H	-2.58858793	-4.16107680	0.44090548
C	-3.50274455	-0.85019479	0.20600580
H	-4.14069500	-0.19784273	0.81258800
C	4.56969161	0.35370227	-0.02618781
H	5.17095693	-0.03891606	-0.84511085
H	4.55434975	1.44337123	-0.11595932
C	5.21393597	-0.04078088	1.30910568
H	5.27032657	-1.12668633	1.38943408
H	6.22553173	0.36340885	1.38158930
H	4.62976262	0.34341873	2.14925579

C	3.78179743	-2.31781334	-1.15462272
O	4.95370271	-2.34722141	-0.86606505
H	3.40243003	-2.99418825	-1.94130384
C	-2.03793078	0.96503387	-0.52685264
O	-1.47113641	1.10649767	-1.58086023
H	-2.65828695	1.78287555	-0.11361543
C	-2.04925773	-0.29961190	0.30294638
H	-1.89112340	0.01971731	1.34282612
C	-0.09665788	3.73030263	-0.64564813
O	-0.62065948	3.26092145	0.58981763
C	-1.06048746	4.37802201	1.37526205
C	-0.58014322	5.64295129	0.64487447
C	0.48777630	5.09120610	-0.30667016
H	0.62734671	2.97941843	-0.97252059
H	-2.14798380	4.34682651	1.47515479
H	-0.61349511	4.27668576	2.36696756
H	-1.39412936	6.08246321	0.06831063
H	-0.19382841	6.39403018	1.33315826
H	0.63818359	5.69828084	-1.19727855
H	1.44135816	4.95686619	0.20892592
O	-1.10129565	3.90531888	-1.59969190
H	-1.36478156	3.02504528	-1.90441211
C	1.00450930	-2.09899570	2.83010074
H	1.91415275	-1.68390763	2.39340883
H	1.23347585	-2.56211312	3.78953164
H	0.28122615	-1.29557420	2.97420416
H	0.58634475	-2.84222328	2.14991239

Coal-NH_2&Tet&CH_4

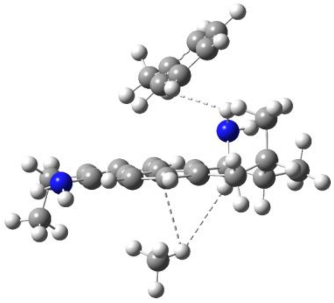

C	-0.82972473	1.06546076	1.39599764
C	-0.32547081	1.09029197	0.11296657
C	-0.08233712	0.44164328	2.42807529
C	0.96116278	0.51258210	-0.16296110
C	1.71047093	-0.07797414	0.89151506
C	1.14135010	-0.11435151	2.19154618
C	3.02387953	-0.59032955	0.63957251
C	3.54985337	-0.55649184	-0.64008070
C	2.78142980	0.02400643	-1.67300909
C	1.53695351	0.55136900	-1.45908740
H	-0.50859475	0.40400620	3.42589665
H	1.65736117	-0.61016185	3.00477422
H	3.20653032	0.04920962	-2.67208164
H	0.97137943	0.95544120	-2.28575074
C	-3.75810864	0.67056545	0.16697390
H	-3.04553207	-0.15477213	0.09544935
H	-4.49586017	0.37834657	0.91885724
H	-4.27864248	0.76457661	-0.78885256
C	-3.07999635	3.23164691	-1.62376178
H	-3.68501633	2.46460064	-2.11276923
H	-3.76473083	3.98989487	-1.23697064
H	-2.45471666	3.70447683	-2.38725424
N	-1.44906152	0.76756888	-2.03663732
H	-2.00677161	0.02108765	-1.63527018
H	-1.99334828	1.19379502	-2.77871425
N	3.74296837	-1.16118952	1.69927310
H	3.60286730	-0.71155117	2.59191716
H	4.72834748	-1.27406383	1.51584424
C	-2.14651279	1.70224370	1.78629561
H	-2.64692825	1.05616088	2.51568487
H	-1.92812835	2.64510850	2.30303159
C	-1.08524031	1.76593013	-1.02125024
H	-0.37919190	2.44703046	-1.51558335
C	-2.22923481	2.65784050	-0.49361569
H	-1.73209758	3.50516792	-0.00067059

C	-3.06504717	1.96832837	0.59520167
H	-3.83874130	2.67897245	0.90833911
C	4.94439435	-1.05199893	-0.93643105
H	5.14756848	-1.98159082	-0.39504657
H	5.00872843	-1.30653827	-1.99699067
C	6.01106243	0.00254404	-0.60753291
H	5.96139613	0.30819403	0.44104451
H	7.01731599	-0.37177956	-0.80772472
H	5.85030722	0.89797126	-1.21156971
C	-2.27672545	-2.48965578	1.46296547
C	-1.17835076	-2.37303798	0.61443368
C	-1.31549082	-2.52877898	-0.76495157
C	-2.59250422	-2.78595919	-1.27636191
C	-3.69213819	-2.90801953	-0.43326175
C	-3.53649797	-2.76690955	0.94365739
H	-2.14230470	-2.36502171	2.53199598
H	-0.19813816	-2.15857831	1.02602442
H	-2.71888262	-2.90643307	-2.34833610
H	-4.67078742	-3.11702117	-0.85030178
H	-4.39166423	-2.86447637	1.60221376
C	-0.12424193	-2.42731093	-1.68186135
H	0.80405972	-2.34760124	-1.11245580
H	-0.06196999	-3.30528818	-2.32981877
H	-0.19922198	-1.53721313	-2.31208447
C	2.50854590	3.22390216	0.75429122
H	1.83127320	3.51415527	-0.04945201
H	3.06562083	4.09533424	1.09828321
H	1.92655653	2.80433341	1.57597029
H	3.19572556	2.46435031	0.37796696

Coal-NH_2&Met&CH_4

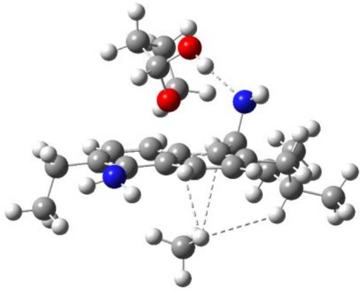

C	-0.10379835	2.47973962	-0.92946411
C	0.08766334	1.24315514	-0.34861403
C	-1.36336443	2.81585996	-1.47164420
C	-0.98942255	0.31235679	-0.25011734
C	-2.26990690	0.68603722	-0.75241851
C	-2.41375904	1.94066648	-1.39539614
C	-3.38802683	-0.19276481	-0.57614044
C	-3.21373555	-1.43097112	0.01530886
C	-1.92547590	-1.79154779	0.47009550
C	-0.84378163	-0.96054254	0.36098205
H	-1.49295426	3.78031533	-1.95154227
H	-3.35697673	2.21605705	-1.85082397
H	-1.80196368	-2.76433329	0.93767655
H	0.11384954	-1.28578825	0.74383552
C	2.58103207	4.63453371	0.75661946
H	3.44506751	4.54837688	0.09270876
H	2.09522015	5.59081124	0.54842863
H	2.94811216	4.66651195	1.78517812
C	3.66420392	1.98021583	0.89812199
H	4.07755767	2.24935290	-0.07803015
H	4.11923744	2.63955773	1.63782328
H	3.98044001	0.95888357	1.13149466
N	2.23369016	0.48930829	-1.10544039
H	2.10656457	1.15055196	-1.86445548
H	3.23008690	0.42247927	-0.92095338
N	-4.64256784	0.20634740	-1.05564865
H	-4.83185292	1.19376416	-0.96744980
H	-5.41487187	-0.33346940	-0.69618192
C	1.01779981	3.48692729	-0.87476934
H	1.81469225	3.26644878	-1.59674702
H	0.63673985	4.47964230	-1.12928451
C	1.49583623	0.91606437	0.10430869
H	1.50305702	0.04501348	0.75975887
C	2.13691298	2.08462234	0.92742679
H	1.83192636	1.89883117	1.96269139

C	1.59026120	3.49045074	0.54942117
H	0.73246792	3.67928889	1.20440365
C	-4.37386938	-2.36894902	0.24416047
H	-5.03876817	-2.37959070	-0.62556728
H	-3.98903745	-3.38746511	0.33505047
C	-5.16082880	-2.01480321	1.51432851
H	-5.53969669	-0.98988427	1.47768101
H	-6.00863002	-2.68798210	1.65864336
H	-4.51144479	-2.08677297	2.38927598
C	1.79653543	-3.01587998	-0.67189895
O	2.07641330	-2.47063814	0.61070301
C	3.31162421	-3.01111196	1.09517373
C	3.71149222	-4.13753097	0.12646612
C	2.40537531	-4.40787015	-0.62963292
H	0.70832584	-2.99420161	-0.77847525
H	4.06476697	-2.22024261	1.13974029
H	3.13333430	-3.37958301	2.10826068
H	4.47347457	-3.78867105	-0.57033414
H	4.09214584	-5.01424233	0.65007580
H	2.55484365	-4.81150199	-1.62954822
H	1.75160431	-5.07312709	-0.06129829
O	2.42033338	-2.28830905	-1.68095764
H	2.16265269	-1.34827054	-1.58285695
C	-1.92325677	1.85451418	2.44750390
H	-2.32164483	2.35985787	3.32725976
H	-0.88446157	1.57233371	2.62309290
H	-2.50160884	0.95301424	2.23937424
H	-1.97114896	2.51698885	1.58182752

Methane and small molecular organic matter are on the same side of coal molecules.

Coal-CH_3&Met&CH_4

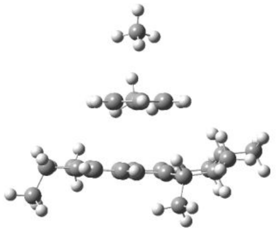

C	-3.49920500	-1.39599700	-0.52098300
C	-1.99914100	-1.67790600	-0.79964600
C	0.37476000	-1.20086800	0.01226000
C	1.29727100	-0.84203500	1.03548100
C	0.77909300	-0.49322400	2.31071400
C	-0.56903300	-0.45658800	2.52690700
C	-1.49329700	-0.80156200	1.51190600
C	-1.03600700	-1.21036500	0.27849900
C	-3.72380400	-0.27725700	0.49958400
C	-2.96884800	-0.63868400	1.77680500
C	-4.22560100	-1.13933000	-1.84287300
C	-5.20464400	-0.04204700	0.79006200
C	2.70562000	-0.80301700	0.77407100
C	3.17741100	-1.10621000	-0.48755100
C	2.25076400	-1.45693300	-1.50073500
C	0.90520200	-1.50167200	-1.27045500
C	4.64823900	-1.11512300	-0.84060400
C	5.34305000	-2.40258800	-0.38135300
H	-0.95183700	-0.14995300	3.49592700
H	1.45213800	-0.22487400	3.11500900
H	-1.73866600	-1.14240600	-1.72198500
H	-3.92575600	-2.30395400	-0.07032600
H	-3.28692100	0.64733900	0.10009600
H	-3.39676000	-1.56085700	2.19375800
H	-3.11069300	0.13862000	2.53461600
H	-3.95757300	-1.89039700	-2.58994900
H	-3.94705700	-0.15819800	-2.24212400
H	-5.31023900	-1.16335600	-1.72587100
H	-5.68956000	-0.97743400	1.08918100
H	-5.32924400	0.67468600	1.60561900
H	-5.73514500	0.34990100	-0.07925900
H	2.62895200	-1.67662300	-2.49456100
H	0.24254500	-1.74100000	-2.09122600

H	4.74629400	-1.02330800	-1.92536800
H	5.15478000	-0.24513600	-0.41587500
H	6.39948900	-2.40311200	-0.65857400
H	5.27175800	-2.52029000	0.70231700
H	4.86722400	-3.27305300	-0.83835000
C	-1.83151100	-3.19036300	-1.03869900
H	-0.81871200	-3.46178800	-1.33471300
H	-2.06068900	-3.72747900	-0.11436600
H	-2.52196700	-3.53814200	-1.81186700
C	3.63554900	-0.40634700	1.89562500
H	3.53346900	-1.08324200	2.74825800
H	4.67856300	-0.42077300	1.58887800
H	3.40810400	0.60239200	2.25375200
C	-1.09569700	1.91757300	-1.32913300
C	-0.84739300	2.27268200	-0.00551000
C	0.45604400	2.42992400	0.46039100
C	1.51119400	2.20261200	-0.42693400
C	1.27061400	1.84696500	-1.74725300
C	-0.03758400	1.70816500	-2.20584100
H	-2.11741600	1.80302000	-1.67561600
H	-1.67730600	2.42953100	0.67760900
H	2.53364300	2.30000600	-0.07363300
H	2.10351800	1.66306000	-2.41613900
H	-0.22816300	1.42780500	-3.23550800
C	0.72432100	2.88075000	1.87178600
H	-0.09176900	2.59552300	2.53754500
H	1.64562500	2.43863700	2.25644200
H	0.83290200	3.96954600	1.90982500
C	0.31101500	5.50550800	-0.93833100
H	0.14451600	4.66753800	-1.61527400
H	0.22885400	6.44754800	-1.48009400
H	-0.43414800	5.47453400	-0.14341800
H	1.30696600	5.41930000	-0.50387400

Coal-CH_3&Tet&CH_4

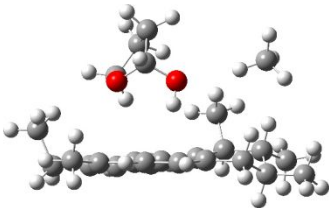

C	3.48954300	-0.73719000	-0.88345500
C	1.97913800	-0.90447600	-1.18137900
C	-0.30974000	-1.27739500	-0.11197800
C	-1.20388100	-1.12026000	0.98179700
C	-0.65874000	-0.76710900	2.24659600
C	0.69223300	-0.62722200	2.40569100
C	1.58432100	-0.73024000	1.30693100
C	1.08966400	-0.99268500	0.04778300
C	3.88880300	-1.29046900	0.48728000
C	3.06305800	-0.56879400	1.54847900
C	4.30909700	-1.34599000	-2.02282600
C	5.38174700	-1.13218500	0.76784700
C	-2.61292500	-1.31677400	0.81298200
C	-3.09898400	-1.76025800	-0.40069300
C	-2.18987800	-1.98635200	-1.46547600
C	-0.85171600	-1.73789200	-1.34256900
C	-4.57123900	-1.97149400	-0.67318500
C	-5.28072400	-0.65820300	-1.02286600
H	1.10146000	-0.40832400	3.38732200
H	-1.31190200	-0.63494500	3.09887800
H	1.86358100	-1.83177100	-1.75707000
H	3.69683500	0.34150400	-0.84855800
H	3.62938800	-2.35806500	0.52000900
H	3.33543100	0.49553000	1.54938300
H	3.30275900	-0.95340700	2.54497500
H	3.92081000	-1.03715200	-2.99623900
H	4.26564100	-2.43933800	-1.97779100
H	5.35715000	-1.04587200	-1.97668300
H	5.68767600	-0.08994600	0.62579800
H	5.61309800	-1.41175300	1.79867100
H	5.99006400	-1.75506500	0.11018900
H	-2.57870300	-2.35922500	-2.40805700
H	-0.20150600	-1.91897000	-2.18813500
H	-4.67479400	-2.66930500	-1.50811000
H	-5.05940900	-2.44453300	0.18139900
H	-6.34023900	-0.82328900	-1.23043100
H	-5.19697800	0.05940400	-0.20301400
H	-4.82431100	-0.20515300	-1.90650900

C	1.52060800	0.28619200	-2.04743900
H	0.51034500	0.15675900	-2.43721100
H	1.52413000	1.19285000	-1.43633900
H	2.19829200	0.43133300	-2.89291300
C	-3.52267000	-0.99866400	1.97272300
H	-3.38982800	0.04737300	2.26376100
H	-4.57101600	-1.14595900	1.72451100
H	-3.29628200	-1.62324700	2.84118400
C	-1.01103100	2.82646200	0.85981600
O	-2.09686900	1.95694300	0.67899400
C	-2.30857400	1.78939400	-0.73110500
C	-1.56792400	2.95022600	-1.42804700
C	-1.13974800	3.84826800	-0.25727100
H	-1.09556200	3.22855800	1.87293600
H	-1.92433000	0.81546500	-1.04406500
H	-3.38694600	1.81273300	-0.89628600
H	-0.68849800	2.57577900	-1.95214600
H	-2.20126800	3.47191800	-2.14506300
H	-0.20500500	4.37897000	-0.43324500
H	-1.92143300	4.56493500	0.00125100
O	0.21812800	2.16378000	0.68180400
H	0.21470500	1.36976100	1.23176600
C	3.56555200	3.05771500	-0.16114400
H	3.64707800	2.88342300	-1.23452200
H	4.37437400	2.53389600	0.34994500
H	3.64920600	4.12610100	0.03819400
H	2.59997600	2.69561800	0.19487100

Coal-OH&Met&CH$_4$

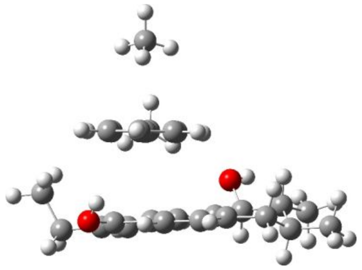

C	3.87910400	0.16399700	-0.82282300
C	2.51373700	-0.45551700	-1.15443700
C	0.28753000	-1.19453600	-0.17569200
C	-0.59984800	-1.30704700	0.92890900
C	-0.12648100	-1.00176900	2.23084300
C	1.16258300	-0.59817300	2.41788100
C	2.04518900	-0.41973200	1.32173500
C	1.60945800	-0.68027500	0.04189300
C	4.43002300	-0.38524200	0.49600800
C	3.46354200	0.00795000	1.60895000
C	4.84389000	-0.03098600	-1.99399600
C	5.84004800	0.11363600	0.80414800
C	-1.93364200	-1.75099700	0.71025600
C	-2.35012700	-2.21600400	-0.51480400
C	-1.43130100	-2.15384700	-1.58632400
C	-0.17094400	-1.63325200	-1.44465400
C	-3.75299800	-2.73114500	-0.71306000
C	-4.76980000	-1.59997000	-0.89527600
O	-2.83314000	-1.76100100	1.74514400
O	1.80628200	0.39883300	-2.06101100
H	1.53216200	-0.40654100	3.42011800
H	-0.77577200	-1.13518900	3.08847200
H	2.68305300	-1.42774900	-1.64526100
H	3.70126500	1.23929400	-0.68131300
H	4.44878100	-1.48238900	0.42971500
H	3.50581300	1.09737300	1.74691200
H	3.78096900	-0.43513100	2.55803100
H	4.39773300	0.25849200	-2.95020800
H	5.14055500	-1.08179000	-2.07236600
H	5.74689600	0.56887800	-1.87539900
H	5.87772500	1.20723800	0.76086900
H	6.14236300	-0.19197000	1.80863300
H	6.57717600	-0.27905300	0.10204900
H	-1.75527100	-2.51044300	-2.55961400
H	0.47516400	-1.55631800	-2.30735400
H	-3.76605800	-3.38460900	-1.58952700

H	-4.04192300	-3.33761700	0.14798400
H	-5.77400600	-1.99842000	-1.05584600
H	-4.78807800	-0.96601100	-0.00669300
H	-4.50576000	-0.97916000	-1.75560700
H	-2.70693600	-0.95784700	2.26259300
H	2.36089500	0.54428200	-2.83254400
C	-2.94552100	1.61483900	1.29775200
C	-2.84908300	1.40460100	-0.07666500
C	-1.64205100	1.58832600	-0.74820100
C	-0.52225300	1.98247700	-0.00983400
C	-0.61196000	2.19794400	1.35908500
C	-1.82584200	2.01591700	2.02068400
H	-3.89969200	1.47997100	1.79720700
H	-3.72569300	1.09857100	-0.63700400
H	0.42920500	2.09530300	-0.52038600
H	0.26985300	2.49226800	1.91711400
H	-1.89594600	2.18739300	3.08909200
C	-1.54728800	1.40597500	-2.23945300
H	-1.72856800	2.35838100	-2.74894800
H	-0.55407200	1.04860700	-2.51643500
H	-2.28829300	0.68590600	-2.59251700
C	-2.46292600	4.86602900	-0.74285200
H	-2.39199500	4.41205400	0.24564100
H	-3.22964900	4.34769500	-1.31858400
H	-2.72247000	5.92075100	-0.65344000
H	-1.50280200	4.76591500	-1.24888200

Coal-OH&Tet&CH_4

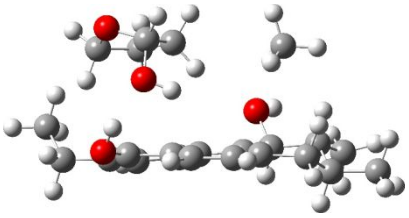

C	3.92863100	-0.54334600	-0.70931600
C	2.52329300	-1.16200700	-0.68689300
C	0.22422000	-1.03990200	0.38543000
C	-0.69928700	-0.41475600	1.26361800
C	-0.23508600	0.59740000	2.14483800
C	1.07338200	0.98843800	2.12041600
C	1.99670400	0.42041300	1.20263100
C	1.57465900	-0.55517200	0.32838700
C	4.39603600	-0.18237200	0.70359100
C	3.43701300	0.86381900	1.26501400
C	4.89984600	-1.47652900	-1.43455600
C	5.83279000	0.33385000	0.73759400
C	-2.06595900	-0.82935400	1.27462800
C	-2.47744100	-1.93776500	0.56604500
C	-1.51383200	-2.60942600	-0.22485200
C	-0.22534900	-2.16383100	-0.35681300
C	-3.90964100	-2.41426800	0.59649700
C	-4.52793400	-2.55656300	-0.79873900
O	-2.96820400	-0.13044000	2.02500400
O	1.90282700	-1.01400800	-1.96661000
H	1.43261500	1.72778900	2.82946500
H	-0.91829900	1.01310800	2.87737700
H	2.62257400	-2.23630500	-0.46011200
H	3.84404800	0.39387500	-1.27542800
H	4.32810400	-1.08335600	1.32978000
H	3.56524000	1.79933800	0.70389200
H	3.69207600	1.08915700	2.30526800
H	4.50132800	-1.82088000	-2.39357700
H	5.10582800	-2.36207000	-0.82480500
H	5.84856600	-0.98299700	-1.64820200
H	5.95818000	1.16254200	0.03263000
H	6.08363100	0.70218100	1.73518400
H	6.55427200	-0.44292100	0.47973700
H	-1.82378700	-3.49033100	-0.77739900
H	0.45091700	-2.67152400	-1.02908200
H	-3.94903800	-3.38216100	1.10820600
H	-4.49725400	-1.71896000	1.19611600

H	-5.57452900	-2.85863100	-0.72578500
H	-4.48484200	-1.61011800	-1.34212800
H	-4.00720400	-3.30599000	-1.39797200
H	-2.88560000	0.81344400	1.79893300
H	2.47589700	-1.40943500	-2.62908200
C	-2.41260700	2.77455800	-0.44981900
O	-3.68998700	2.45360300	-0.87352200
C	-3.69447700	1.06496500	-1.21351100
C	-2.31585800	0.79039500	-1.83598400
C	-1.44803700	1.96219200	-1.32609700
H	-2.29969100	3.85741300	-0.53321700
H	-3.85811500	0.46611200	-0.31221900
H	-4.52760000	0.90698900	-1.89754500
H	-1.91720400	-0.17730200	-1.53256300
H	-2.37491600	0.80502600	-2.92446700
H	-0.57599800	1.62183400	-0.76257100
H	-1.09241500	2.58087100	-2.15076400
O	-2.29797900	2.40675500	0.92989700
H	-1.36312800	2.43073700	1.16486600
C	1.99475500	2.69844800	-1.51815600
H	1.79021600	1.65618100	-1.76798900
H	1.47224400	3.35382400	-2.21483300
H	1.64945300	2.89756100	-0.50228700
H	3.06627000	2.89259500	-1.58129500

Coal-COOH&Met&CH_4

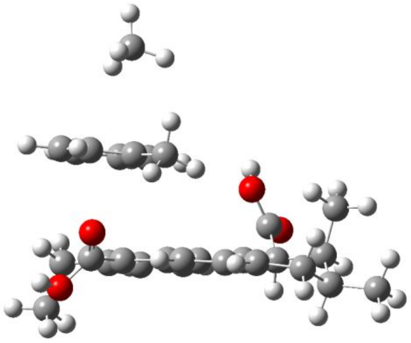

C	2.02754600	-1.31878700	1.21199200
C	1.58992900	-1.59552600	-0.06739200
C	0.23074300	-1.36981400	-0.46091900
C	-0.68870500	-0.82560300	0.47703700
C	-0.21769500	-0.57703000	1.79385000
C	1.08148200	-0.81844400	2.13921200
C	-0.25498000	-1.72694400	-1.74892800
C	-2.05681800	-0.59497900	0.10192900
C	-1.57372300	-1.56964200	-2.05417700
H	0.42078200	-2.13681600	-2.48640900
H	-0.88993800	-0.20783100	2.55895200
H	1.41479300	-0.60807900	3.15061000
H	-1.93978500	-1.88305500	-3.02674500
C	-6.34600000	-0.80180200	-0.73268000
H	-6.53450700	0.01916100	-1.42829900
H	-6.64273800	-1.72731100	-1.23181300
H	-6.99326700	-0.66565600	0.13724500
C	-4.47523000	1.65335500	-0.22965100
H	-3.67103600	1.72296300	-0.96575000
H	-5.43014100	1.77237700	-0.74360800
H	-4.36974200	2.49920200	0.45254000
C	-2.54130500	1.28389100	1.78189500
C	-4.43165200	0.32468400	0.53542600
H	-5.10374500	0.40647900	1.39562700
C	-4.87743100	-0.86745600	-0.32153800
H	-4.73702300	-1.76967300	0.28782000
C	-3.95555400	-0.97883300	-1.53098200
H	-4.14275000	-0.14498400	-2.21902500
H	-4.18458500	-1.89069400	-2.09125600
C	-2.49878600	-1.01381400	-1.13414700
C	-3.02129100	0.01049500	1.11218000
H	-3.16043500	-0.69786900	1.93577000
C	2.54326700	-2.10982200	-1.09706900
O	2.66550100	-1.68318300	-2.21572400

O	3.27689300	-3.15356600	-0.66052300
H	3.87191100	-3.39927200	-1.38272700
C	3.44321600	-1.50773600	1.70462700
H	4.15241600	-1.46529500	0.87803600
H	3.67880000	-0.66225700	2.35671800
C	3.60673600	-2.81866800	2.48193400
H	4.62435700	-2.91444600	2.86618600
H	2.91935400	-2.85288100	3.33075800
H	3.39592700	-3.67207000	1.83696700
O	-2.86962900	1.62211400	2.88773000
O	-1.76433200	2.05499900	0.99997900
H	-1.57091100	2.85969000	1.50192300
C	4.28087600	1.46030700	-0.26053400
C	3.37526800	1.26644200	-1.29654400
C	2.01338300	1.52476400	-1.11582900
C	1.58070300	1.95993600	0.13468400
C	2.48355700	2.15682500	1.17659800
C	3.83851100	1.91476800	0.98105500
H	5.33453200	1.25934300	-0.41991200
H	3.71985100	0.90123000	-2.25828700
H	0.52019100	2.12063000	0.29337200
H	2.12627100	2.49162700	2.14459000
H	4.54480700	2.06894600	1.78898400
C	1.04279300	1.35800300	-2.25391000
H	0.01889600	1.25977500	-1.88466000
H	1.08517900	2.22847000	-2.91677100
H	1.28816400	0.47047700	-2.84042300
C	2.86000700	4.74012400	-1.70792500
H	3.15727900	5.78468800	-1.79907200
H	1.77533400	4.67316700	-1.62413900
H	3.18749500	4.18798500	-2.58871300
H	3.31370700	4.29688200	-0.82136900

Coal-COOH&Tet&CH_4

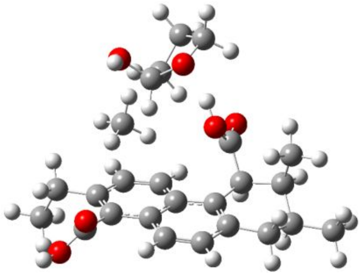

C	-2.93185800	-0.25908600	-0.72372500
C	-2.60693400	-1.39398100	-0.01156600
C	-1.25721800	-1.85057700	0.10500800
C	-0.21655300	-1.10172100	-0.50486300
C	-0.58019700	0.04233500	-1.26423200
C	-1.88409400	0.44000100	-1.36955000
C	-0.91642900	-3.05241600	0.78441700
C	1.15056500	-1.51344000	-0.35966900
C	0.37765500	-3.48081600	0.80698800
H	-1.68727900	-3.63467300	1.27113900
H	0.17765600	0.61591100	-1.78572500
H	-2.13133500	1.32609400	-1.94698700
H	0.62316700	-4.42227100	1.28835900
C	5.17275400	-3.16428800	-0.66851300
H	5.58524100	-3.09707600	0.34084900
H	5.18013900	-4.21871000	-0.95510800
H	5.84143200	-2.62646000	-1.34522100
C	4.10539500	-0.68398500	0.91215800
H	3.35527200	-0.97019000	1.65242400
H	5.05722100	-1.14597700	1.17909800
H	4.23688800	0.39717500	0.98583100
C	2.10760600	0.83184400	-0.39009500
C	3.68650200	-1.08680200	-0.50690900
H	4.36802900	-0.59898000	-1.21132600
C	3.75443900	-2.60402000	-0.73596500
H	3.36424100	-2.79233600	-1.74474000
C	2.82632200	-3.30076200	0.25483800
H	3.24604400	-3.23689500	1.26648100
H	2.76409600	-4.36832300	0.01964900
C	1.43150300	-2.72342900	0.23200100
C	2.25576700	-0.60468800	-0.87391100
H	2.20213800	-0.54586000	-1.96584100
C	-3.67408200	-2.14536600	0.71790500
O	-3.66412000	-2.40321100	1.89138400
O	-4.68633400	-2.52786200	-0.08704700
H	-5.33371000	-2.98355800	0.46955500

C	-4.33044800	0.29877600	-0.84451500
H	-4.95645400	-0.04080500	-0.01805400
H	-4.26548100	1.38806800	-0.76156900
C	-4.98289800	-0.07442800	-2.18030900
H	-5.97485000	0.37367800	-2.26478700
H	-4.37755200	0.27960400	-3.01804700
H	-5.08015000	-1.15748100	-2.26193400
O	2.45325800	1.78568700	-1.05009400
O	1.60166900	0.93497500	0.83142700
H	1.46829500	1.89384200	1.04659000
C	-0.29069000	3.48438800	0.29267600
O	0.87170900	3.46305600	1.11935400
C	1.78490000	4.51262300	0.72982800
C	1.06020700	5.30949900	-0.35437600
C	0.11956200	4.25569700	-0.94803800
H	-0.56211300	2.44031900	0.11526600
H	2.02785300	5.10056700	1.61463200
H	2.68512500	4.03290600	0.33869200
H	0.48097100	6.12217700	0.08607800
H	1.75360300	5.72270000	-1.08589700
H	-0.74611900	4.67354700	-1.45930100
H	0.66752600	3.58815900	-1.61617800
O	-1.33467200	4.16731300	0.92501900
H	-1.56212400	3.68449100	1.72632800
C	-1.73064400	1.00494500	2.24002900
H	-2.42064300	1.54252500	1.58586500
H	-1.36719100	1.66198100	3.03292200
H	-2.25417900	0.15824300	2.68479300
H	-0.88463800	0.63613300	1.65814700

Coal-C=O&Met&CH_4

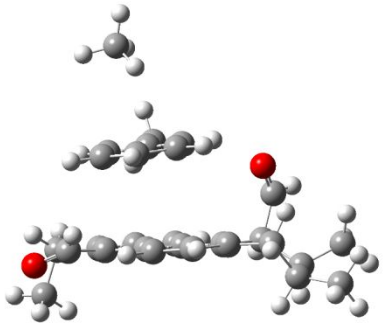

C	-1.76762800	0.01998400	-1.07316200
C	-1.48708800	-0.84910600	-0.04462000
C	-0.75094500	0.38156800	-1.98292200
C	-0.15200700	-1.31860800	0.15199200
C	0.87732100	-0.92515300	-0.75234900
C	0.52603900	-0.08415900	-1.84213300
C	2.22128800	-1.39117700	-0.54754500
C	2.53644700	-2.22398900	0.51611600
C	1.49587300	-2.59470500	1.40318000
C	0.21074000	-2.16182800	1.23559900
H	-0.99202700	1.04721500	-2.80490800
H	1.26536400	0.21120500	-2.57329800
H	1.74098700	-3.23079300	2.24753900
H	-0.53217100	-2.46347100	1.96285300
C	-5.52258200	-0.43766300	-1.64093200
H	-5.97938800	0.32754900	-1.00852200
H	-5.51245500	-0.06015300	-2.66572200
H	-6.16294100	-1.32298500	-1.62050200
C	-5.16550100	-1.08641800	1.12945100
H	-5.21550900	-0.01070200	1.32624200
H	-6.11974200	-1.38910300	0.69913700
H	-5.06616400	-1.60009900	2.08886800
C	-4.09671800	-0.76082900	-1.19827500
H	-3.68180200	-1.46748900	-1.92445300
C	-3.18856800	0.47474000	-1.25298900
H	-3.46732900	1.18666600	-0.46590900
H	-3.30491000	0.98763500	-2.21094600
C	-4.00622100	-1.44614100	0.19167600
H	-4.06484300	-2.52356200	0.01585400
C	3.91293400	-2.77447900	0.80185300
H	4.68691600	-2.07010700	0.50018700
H	3.99893500	-2.92030000	1.88234900
C	4.14209900	-4.11242600	0.08752600
H	4.10071800	-3.97026600	-0.99244200
H	5.12145500	-4.52055100	0.34489900

H	3.38073300	-4.84157500	0.37558500
C	3.29189900	-0.89977200	-1.45706000
O	4.30916000	-1.49874400	-1.71223400
H	3.12874800	0.10150000	-1.88927400
C	-2.73271600	-0.14652700	1.99385300
O	-2.33667000	0.98400600	1.91367900
H	-3.22363400	-0.49042800	2.92472000
C	-2.61423100	-1.20451800	0.89619600
H	-2.39735600	-2.13131700	1.43222100
C	2.45886100	2.91576000	-1.15750100
C	2.42642700	2.14549200	-0.00055300
C	1.25621800	2.02734800	0.75290000
C	0.11404900	2.69953200	0.31918500
C	0.14342900	3.47843100	-0.83463000
C	1.31335000	3.58854900	-1.57878500
H	3.37830100	2.99820400	-1.72707100
H	3.32292500	1.62685800	0.32933400
H	-0.80646100	2.59402000	0.88319900
H	-0.75396900	3.99581200	-1.15602300
H	1.33637300	4.19413300	-2.47739700
C	1.24135400	1.21238900	2.01851000
H	1.68805000	1.77994600	2.84128300
H	0.21928000	0.95413700	2.29910500
H	1.81462800	0.28949200	1.89731700
C	2.22162200	4.96951300	2.10506700
H	1.32534400	4.61630500	2.61477800
H	2.03150100	5.00002100	1.03214200
H	2.48212500	5.96368500	2.46769000
H	3.04394500	4.28157200	2.30171200

Coal-C=O&Tet&CH_4

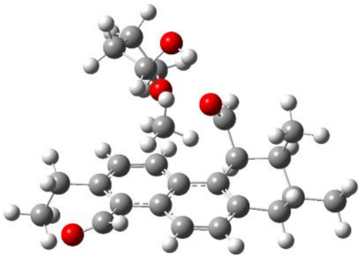

C	-1.78198700	0.08283900	-1.05921600
C	-1.52708900	-0.81442600	-0.04830900
C	-0.75415500	0.43435700	-1.96038300
C	-0.20600000	-1.32554000	0.13929200
C	0.83545600	-0.94172100	-0.75520500
C	0.50943100	-0.06880500	-1.82753000
C	2.16568400	-1.44897600	-0.55879300
C	2.45539800	-2.31444100	0.48590900
C	1.40328700	-2.67520300	1.36341100
C	0.13128100	-2.20209000	1.20457500
H	-0.97555500	1.12268400	-2.76912000
H	1.25795200	0.22121300	-2.55145600
H	1.62891600	-3.33693400	2.19336500
H	-0.62084400	-2.49860700	1.92436800
C	-5.54883700	-0.25419400	-1.63365100
H	-5.98225400	0.51446600	-0.98908200
H	-5.52779900	0.13868700	-2.65248500
H	-6.21540200	-1.12018800	-1.62633800
C	-5.20868700	-0.95418800	1.12729400
H	-5.22122200	0.11923100	1.34175500
H	-6.17364000	-1.21685400	0.69456600
H	-5.12500400	-1.48663000	2.07796200
C	-4.13326500	-0.62687300	-1.19703600
H	-3.73922500	-1.33318900	-1.93511700
C	-3.18903600	0.58193300	-1.23061100
H	-3.44655500	1.28786600	-0.43104100
H	-3.29007400	1.11452100	-2.17953900
C	-4.06407500	-1.33789000	0.18096100
H	-4.16005200	-2.40954700	-0.01279500
C	3.81508400	-2.91068200	0.75998300
H	4.60940900	-2.22113900	0.47760900
H	3.89440400	-3.08611800	1.83656000
C	4.00793100	-4.23593500	0.01209000
H	3.97341100	-4.06520000	-1.06398200
H	4.97460100	-4.67827600	0.26073900
H	3.22540300	-4.95021200	0.27999000

C	3.25159900	-0.96527200	-1.45425200
O	4.25298200	-1.58519900	-1.72183100
H	3.11683700	0.05038600	-1.86207700
C	-2.74734200	-0.11831200	2.00657100
O	-2.33416300	1.00702800	1.94159400
H	-3.23063500	-0.47182100	2.93797400
C	-2.66353600	-1.15618300	0.88655500
H	-2.47448700	-2.10098500	1.40090200
C	2.51478600	2.86217400	-1.08467300
C	2.46569400	2.07642700	0.06128600
C	1.29307000	1.97249200	0.81319500
C	0.16728700	2.67835200	0.39083600
C	0.21348700	3.47280300	-0.75223400
C	1.38505500	3.56654800	-1.49675300
H	3.43600600	2.93252400	-1.65306300
H	3.35049100	1.53317700	0.38301900
H	-0.75569200	2.58528700	0.95270000
H	-0.67243500	4.01429600	-1.06565500
H	1.42269800	4.18344100	-2.38713400
C	1.25619100	1.12847200	2.05969300
H	1.73493800	1.65292300	2.89246100
H	0.22691700	0.90622100	2.34484300
H	1.78874800	0.18575400	1.90916100
C	2.73388800	5.00200000	1.89155600
H	2.49849800	4.28741700	2.68014900
H	1.98113100	4.92758700	1.10693200
H	2.74433000	6.01019900	2.30519000
H	3.71203800	4.76718700	1.47316700

Coal-NH_2&Met&CH_4

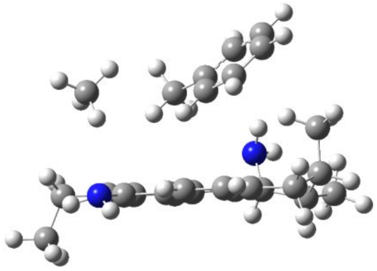

C	1.18261800	-1.12415100	1.52157800
C	0.67651700	-1.38202600	0.26506600
C	0.32485000	-0.61565200	2.53068400
C	-0.71656900	-1.15166100	-0.00745400
C	-1.56429400	-0.65827700	1.02322100
C	-0.99984800	-0.38832900	2.29800300
C	-2.95255700	-0.42620800	0.76090600
C	-3.46151300	-0.61462000	-0.51159200
C	-2.60536800	-1.12266600	-1.51335400
C	-1.28539300	-1.40225700	-1.28346400
H	0.74760700	-0.39292600	3.50550600
H	-1.60426100	0.04059200	3.08803300
H	-3.02004300	-1.28595300	-2.50378900
H	-0.65816200	-1.74804700	-2.09182200
C	3.88207500	-0.09010100	0.16388900
H	2.97927800	0.51863500	0.06908100
H	4.53741700	0.43485100	0.86387000
H	4.38612000	-0.11703600	-0.80489100
C	3.86088900	-2.87199900	-1.41968900
H	4.23958700	-2.01492100	-1.98146600
H	4.72450800	-3.39823900	-1.00658500
H	3.36582300	-3.54542500	-2.12599800
N	1.64425900	-0.94884500	-1.94011400
H	1.99211600	-0.05576600	-1.60751000
H	2.27441000	-1.27379100	-2.66526900
N	-3.76334500	0.06597400	1.79500800
H	-3.53684800	-0.29133500	2.71139100
H	-4.75310500	-0.02192000	1.62031300
C	2.62444000	-1.37782300	1.90734200
H	2.95626600	-0.57489700	2.57430200
H	2.66348400	-2.30526300	2.49214500
C	1.56447000	-1.92794300	-0.84668100
H	1.04992600	-2.80172500	-1.26831600
C	2.91058700	-2.45147000	-0.30126700
H	2.66165600	-3.35765300	0.26834900
C	3.55844900	-1.48744700	0.70411800

H	4.49616000	-1.94831600	1.03580100
C	-4.91169700	-0.34396700	-0.82993800
H	-5.24617900	0.57030300	-0.32786900
H	-5.00284400	-0.14399500	-1.90049300
C	-5.82364400	-1.52265200	-0.46079000
H	-5.74313300	-1.77593400	0.59967800
H	-6.86989800	-1.29936700	-0.68018500
H	-5.53567100	-2.41126600	-1.02608800
C	1.61755400	2.69843500	1.27015400
C	0.59333300	2.23545800	0.44705400
C	0.70286800	2.30009200	-0.94211300
C	1.87781800	2.82931200	-1.48826800
C	2.90098900	3.29828900	-0.67117800
C	2.77177600	3.23923900	0.71461700
H	1.50783100	2.63505300	2.34730200
H	-0.30595000	1.81609400	0.88560700
H	1.98188600	2.88360300	-2.56803400
H	3.79950900	3.71150900	-1.11534500
H	3.56800800	3.60470400	1.35258200
C	-0.41385500	1.81950900	-1.83162900
H	-1.26087000	1.47043200	-1.23897900
H	-0.75909200	2.62526000	-2.48572700
H	-0.08099600	0.98471000	-2.45365800
C	-3.26105800	3.08859000	-0.15360600
H	-3.16435700	2.34673100	0.64017300
H	-4.04920300	3.79674000	0.10293300
H	-2.31742300	3.62136700	-0.27437500
H	-3.51057600	2.58523800	-1.08857800

Coal-NH_2&Tet&CH_4

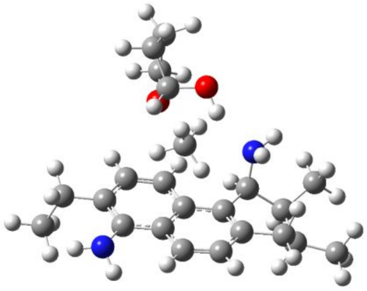

C	-2.57788200	1.15841500	0.53398200
C	-1.58139800	0.47978200	-0.13340600
C	-2.32767100	2.45136100	1.04694700
C	-0.27880300	1.04935800	-0.26504300
C	-0.05133500	2.37035200	0.21724900
C	-1.10510700	3.04555800	0.88269900
C	1.24621900	2.96214000	0.08096500
C	2.30289200	2.21576100	-0.41136900
C	2.06121900	0.88885800	-0.83248000
C	0.81688600	0.32061700	-0.79177600
H	-3.11756300	2.97071500	1.57933100
H	-0.93681800	4.02356500	1.31668100
H	2.89221300	0.29210500	-1.19713800
H	0.70749400	-0.70432100	-1.11815500
C	-5.78731800	-0.48550600	-0.76363700
H	-5.99162100	-1.27343100	-0.03412600
H	-6.43934800	0.36001700	-0.53212700
H	-6.06651200	-0.85935900	-1.75184100
C	-3.81040600	-2.56039900	-0.93830200
H	-4.04512200	-2.74960000	0.11323500
H	-4.71214900	-2.75975300	-1.51753500
H	-3.05756600	-3.28803900	-1.25529200
N	-1.49801500	-1.80165800	0.56191500
H	-1.88267100	-1.43828400	1.42890700
H	-1.84014500	-2.75041300	0.44752100
N	1.43950600	4.27447400	0.53278600
H	0.64916000	4.88650600	0.39296800
H	2.28526900	4.70832800	0.19547700
C	-3.93816800	0.51996500	0.64685600
H	-3.94344600	-0.28633800	1.39325500
H	-4.67295000	1.25834900	0.97886800
C	-1.86929300	-0.93991800	-0.58048000
H	-1.18817500	-1.21409500	-1.38745600
C	-3.31381300	-1.13075600	-1.17054300
H	-3.19622100	-1.01651300	-2.25286700
C	-4.32627500	-0.04202000	-0.72396400

H	-4.22031800	0.79340500	-1.42435100
C	3.68736100	2.80173300	-0.54679300
H	3.93026300	3.41800300	0.32505000
H	4.41336900	1.98531200	-0.54651600
C	3.85082600	3.61676000	-1.83772600
H	3.12084600	4.42865800	-1.89492600
H	4.85054000	4.05047300	-1.91009200
H	3.68943400	2.97689300	-2.70749400
C	2.30922800	-2.33612400	0.66261700
O	2.47701600	-2.32512900	-0.74554100
C	3.01155600	-3.58504800	-1.16554100
C	3.43593700	-4.33379100	0.11095900
C	3.44113300	-3.21949900	1.16427100
H	2.36122900	-1.29277100	0.98350500
H	2.25470700	-4.13391600	-1.73061000
H	3.85781500	-3.37793800	-1.82515800
H	2.69338700	-5.08651400	0.37594100
H	4.40372500	-4.82266300	0.00039700
H	3.25911400	-3.57267000	2.17770200
H	4.37691900	-2.65695400	1.13628100
O	1.10062200	-2.92197200	1.03853100
H	0.34549000	-2.34957300	0.79778700
C	0.58350200	0.26539300	2.83357700
H	1.33261100	0.78679300	2.23514100
H	0.50302400	-0.76789100	2.49427300
H	0.87378400	0.28283400	3.88424500
H	-0.37705500	0.76821200	2.70791400
